# The Neural Representation of Force across Grasp Types in Motor Cortex of Humans with Tetraplegia

**DOI:** 10.1523/ENEURO.0231-20.2020

**Published:** 2021-02-23

**Authors:** Anisha Rastogi, Francis R. Willett, Jessica Abreu, Douglas C. Crowder, Brian A. Murphy, William D. Memberg, Carlos E. Vargas-Irwin, Jonathan P. Miller, Jennifer Sweet, Benjamin L. Walter, Paymon G. Rezaii, Sergey D. Stavisky, Leigh R. Hochberg, Krishna V. Shenoy, Jaimie M. Henderson, Robert F. Kirsch, A. Bolu Ajiboye

**Affiliations:** 1 Department of Biomedical Engineering, Case Western Reserve University, Cleveland, OH 44106; 2 Department of Neurosurgery, Stanford University, Stanford, CA 94035; 3 Department of Electrical Engineering, Stanford University, Stanford, CA 94035; 4 Louis Stokes Cleveland Department of VA Medical Center, Cleveland, OH 44106; 5 Department of Neuroscience, Brown University, Providence, RI 02912; 6 Robert J. and Nancy D. Carney Institute for Brain Sciences, Brown University, Providence, RI 02912; 7 VA RR&D Center for Neurorestoration and Neurotechnology, Providence, RI 02912; 8 Department of Neurological Surgery, University Hospitals Cleveland Medical Center, Cleveland, OH 44106; 9 Department of Neurological Surgery, Case Western Reserve School of Medicine School of Medicine, Cleveland, OH 44106; 10 Department of Neurology, University Hospitals Cleveland Medical Center, Cleveland, OH 44106; 11 School of Engineering, Brown University, Providence, RI 02912; 12 Center for Neurotechnology and Neurorecovery, Department of Neurology, Massachusetts General Hospital, Boston, MA 02114; 13 Department of Neurology, Harvard Medical School, Boston, MA 02114; 14 Department of Bioengineering, Stanford University, Stanford, CA 94035; 15 Department of Neurobiology, Stanford University, Stanford, CA 94035; 16 Howard Hughes Medical Institute at Stanford University, Stanford, CA 94035; 17 Wu Tsai Neuroscience Institute, Stanford University, Stanford, CA 94035; 18 Bio-X Program, Stanford University, Stanford, CA 94035

**Keywords:** brain-computer interface, force, grasp, kinetic, motor cortex

## Abstract

Intracortical brain-computer interfaces (iBCIs) have the potential to restore hand grasping and object interaction to individuals with tetraplegia. Optimal grasping and object interaction require simultaneous production of both force and grasp outputs. However, since overlapping neural populations are modulated by both parameters, grasp type could affect how well forces are decoded from motor cortex in a closed-loop force iBCI. Therefore, this work quantified the neural representation and offline decoding performance of discrete hand grasps and force levels in two human participants with tetraplegia. Participants attempted to produce three discrete forces (light, medium, hard) using up to five hand grasp configurations. A two-way Welch ANOVA was implemented on multiunit neural features to assess their modulation to *force* and *grasp*. Demixed principal component analysis (dPCA) was used to assess for population-level tuning to force and grasp and to predict these parameters from neural activity. Three major findings emerged from this work: (1) force information was neurally represented and could be decoded across multiple hand grasps (and, in one participant, across attempted elbow extension as well); (2) grasp type affected force representation within multiunit neural features and offline force classification accuracy; and (3) grasp was classified more accurately and had greater population-level representation than force. These findings suggest that force and grasp have both independent and interacting representations within cortex, and that incorporating force control into real-time iBCI systems is feasible across multiple hand grasps if the decoder also accounts for grasp type.

## Significance Statement

Intracortical brain-computer interfaces (iBCIs) have emerged as a promising technology to potentially restore hand grasping and object interaction in people with tetraplegia. This study is among the first to quantify the degree to which hand grasp affects force-related, or kinetic, neural activity and decoding performance in individuals with tetraplegia. The study results enhance our overall understanding of how the brain encodes kinetic parameters across varying kinematic behaviors, and in particular, the degree to which these parameters have independent versus interacting neural representations. Such investigations are a critical step to incorporating force control into human-operated iBCI systems, which would move the technology toward restoring more functional and naturalistic tasks.

## Introduction

Intracortical brain-computer interfaces (iBCIs) have emerged as a promising technology to restore upper limb function to individuals with paralysis. Traditionally, iBCIs decode kinematic parameters from motor cortex to control the position and velocity of end effectors. These iBCIs evolved from the seminal work of Georgopoulos and colleagues, who proposed that motor cortex encodes high-level kinematics, including continuous movement directions and three-dimensional hand positions, in a global coordinate frame (Georgopoulos et al., 1982, 1986). Kinematic iBCIs have successfully achieved control of one-dimensional and two-dimensional computer cursors (Wolpaw et al., 2002; Leuthardt et al., 2004; Kübler et al., 2005; Hochberg et al., 2006; Kim et al., 2008, 2011; Schalk et al., 2008; Hermes et al., 2011; Simeral et al., 2011), prosthetic limbs (Hochberg et al., 2012; Collinger et al., 2013; Wodlinger et al., 2015), and paralyzed arm and hand muscles (Bouton et al., 2016; Ajiboye et al., 2017).

While kinematic iBCIs can restore basic reaching and grasping movements, restoring the ability to grasp and interact with objects requires both kinematic and kinetic (force-related) information (Chib et al., 2009; Flint et al., 2014; Casadio et al., 2015). Specifically, sufficient contact force is required to prevent object slippage; however, excessive force may cause mechanical damage to objects (Westling and Johansson, 1984). Therefore, introducing force calibration capabilities during grasp control would enable iBCI users to perform more functional tasks.

Early work by Evarts and others, which showed correlations between cortical activity and force output (Evarts, 1968; Humphrey, 1970; Fetz and Cheney, 1980; Evarts et al., 1983; Kalaska et al., 1989), and later work, which decoded muscle activations from neurons in primary motor cortex (M1; Morrow and Miller, 2003; Sergio and Kalaska, 2003; Pohlmeyer et al., 2007; Oby et al., 2010), suggest that cortex encodes low-level dynamics of movement along with kinematics (Kakei et al., 1999; Carmena et al., 2003; Branco et al., 2019). However, explorations of kinetic parameters as control signals for iBCIs have only just begun. The majority have characterized neural modulation to executed kinetic tasks in primates and able-bodied humans (Filimon et al., 2007; Moritz et al., 2008; Pohlmeyer et al., 2009; Ethier et al., 2012; Flint et al., 2012, 2014, 2017; Schwarz et al., 2018). Small subsets of M1 neurons have been used to command muscle activations through functional electrical stimulation (FES), to restore one-dimensional wrist control and whole-hand grasping in primates with temporary motor paralysis (Moritz et al., 2008; Pohlmeyer et al., 2009; Ethier et al., 2012). More recent intracortical studies demonstrated that force representation is preserved in individuals with chronic tetraplegia (Downey et al., 2018; Rastogi et al., 2020).

Intended forces are usually produced in the context of task-related factors, including grasp postures used to generate forces (Murphy et al., 2016). The representation and decoding of grasps, independent of forces, has been studied extensively in primates (Stark and Abeles, 2007; Stark et al., 2007; Vargas-Irwin et al., 2010; Carpaneto et al., 2011; Townsend et al., 2011; Hao et al., 2014; Schaffelhofer et al., 2015) and humans (Pistohl et al., 2012; Chestek et al., 2013; Bleichner et al., 2014, 2016; Klaes et al., 2015; Leo et al., 2016; Branco et al., 2017). Importantly, previous studies suggest that force and grasp are encoded by overlapping populations of neural activity (Sergio and Kalaska, 1998; Carmena et al., 2003; Sergio et al., 2005; Milekovic et al., 2015; Sburlea and Müller-Putz, 2018). While some studies suggest that force is encoded at a high level independent of motion and grasp (Chib et al., 2009; Hendrix et al., 2009; Pistohl et al., 2012; Intveld et al., 2018), others suggest that it is encoded at a low level intertwined with grasp (Hepp-Reymond et al., 1999; Degenhart et al., 2011). Thus, the degree to which intended hand grasps and forces interact within the neural space, and how such interactions affect force decoding performance, remain unclear. To our knowledge, these scientific questions have not been explored in individuals with tetraplegia, who constitute a target population for iBCI technologies.

To answer these questions, we characterized the extent to which three discrete, attempted forces were neurally represented and offline-decoded across up to five hand grasp configurations in two individuals with tetraplegia. Our results suggest that force has both grasp-independent and grasp-dependent (interacting) representation in motor cortex. Additionally, while this study demonstrates the feasibility of incorporating discrete force control into human-operated iBCIs, these systems will likely need to incorporate grasp and other task parameters to achieve optimal performance.

## Materials and Methods

### Study permissions and participants

Study procedures were approved by the United States Food and Drug Administration (Investigational Device Exemption #G090003) and the Institutional Review Boards of University Hospitals Case Medical Center (protocol #04-12-17), Massachusetts General Hospital (2011P001036), the Providence VA Medical Center (2011-009), Brown University (0809992560), and Stanford University (protocol #20 804). Human participants were enrolled in the BrainGate2 Pilot Clinical Trial (ClinicalTrials.gov number NCT00912041). Informed consent, including consent to publish, was obtained from the participants before their enrollment in the study.

This study includes data from two participants with chronic tetraplegia. Both participants had two, 96-channel microelectrode intracortical arrays (1.5-mm electrode length, Blackrock Microsystems) implanted in the hand and arm area (“hand knob”; Yousry et al., 1997) of dominant motor cortex. Participant T8 was a 53-year-old right-handed male with C4-level AIS-A spinal cord injury eight years before implant, and T5 was a 63-year-old right-handed male with C4-level AIS-C spinal cord injury. More surgical details can be found at (Ajiboye et al., 2017) for T8 and (Nuyujukian et al., 2018) for T5.

### Participant task

The goal of this study was to measure the degree to which various hand grasps affect decoding of grasp force from motor cortical spiking activity. To this end, participants T8 and T5 took part in several research sessions in which they attempted to produce three discrete squeeze forces (light, medium, hard) using one of four designated hand grasps (closed pinch, open pinch, ring pinch, power). Squeeze force, defined here as the amount of force needed to deform an object, is distinct from grip force, which is the amount of force needed to grasp an object of particular weight and friction (Westling and Johansson, 1984). In this study, participants were instructed to produce squeeze forces as opposed to grip forces. This was because participants could not receive somatosensory feedback about the object properties that usually inform grip force production, yet retained the capacity to emulate squeeze forces in response to audio and visual cues.

The four hand grasps used to emulate squeeze forces were chosen to study force representation within multiple grasp-related contexts. The open and closed pinch grasps were included to determine how forces were represented when emulated with grasps of similar function (thumb-index precision grasp) but different posture. The ring pinch grasp was included to determine the effects of using different fingers to produce similar forces. Finally, the power grasp was included to determine the influence of power versus precision grasping on force representation.

Participant T8 completed six sessions between trial days 735–956 relative to the date of his microelectrode array placement surgery; and T5 completed one session on trial day 390. During session 5, participant T8 emulated discrete forces using attempted elbow extension in addition to the four distal hand grasps. This enabled the study of force representation across the entire upper limb. Table 1 lists all relevant sessions and their associated task parameters.

**Table 1.** Session information

Session Number	Participant	Post-Implant Day	Number of Blocks per Grasp				
			Closed Pinch	Open Pinch	Ring Pinch	Power	Elbow
1	T8	Day 735	11	—	—	10	—
2	T8	Day 771	5	5	5	5	—
3	T8	Day 774	6	5	5	5	—
4	T8	Day 788	5	5	5	5	—
5	T8	Day 802	4	4	4	4	5
6	T8	Day 956	4	4	4	4	—
7	T5	Day 390	4	4	4	4	—

Session information for participants T8 and T5, including the number of blocks per grasp type.

**Figure 1.**
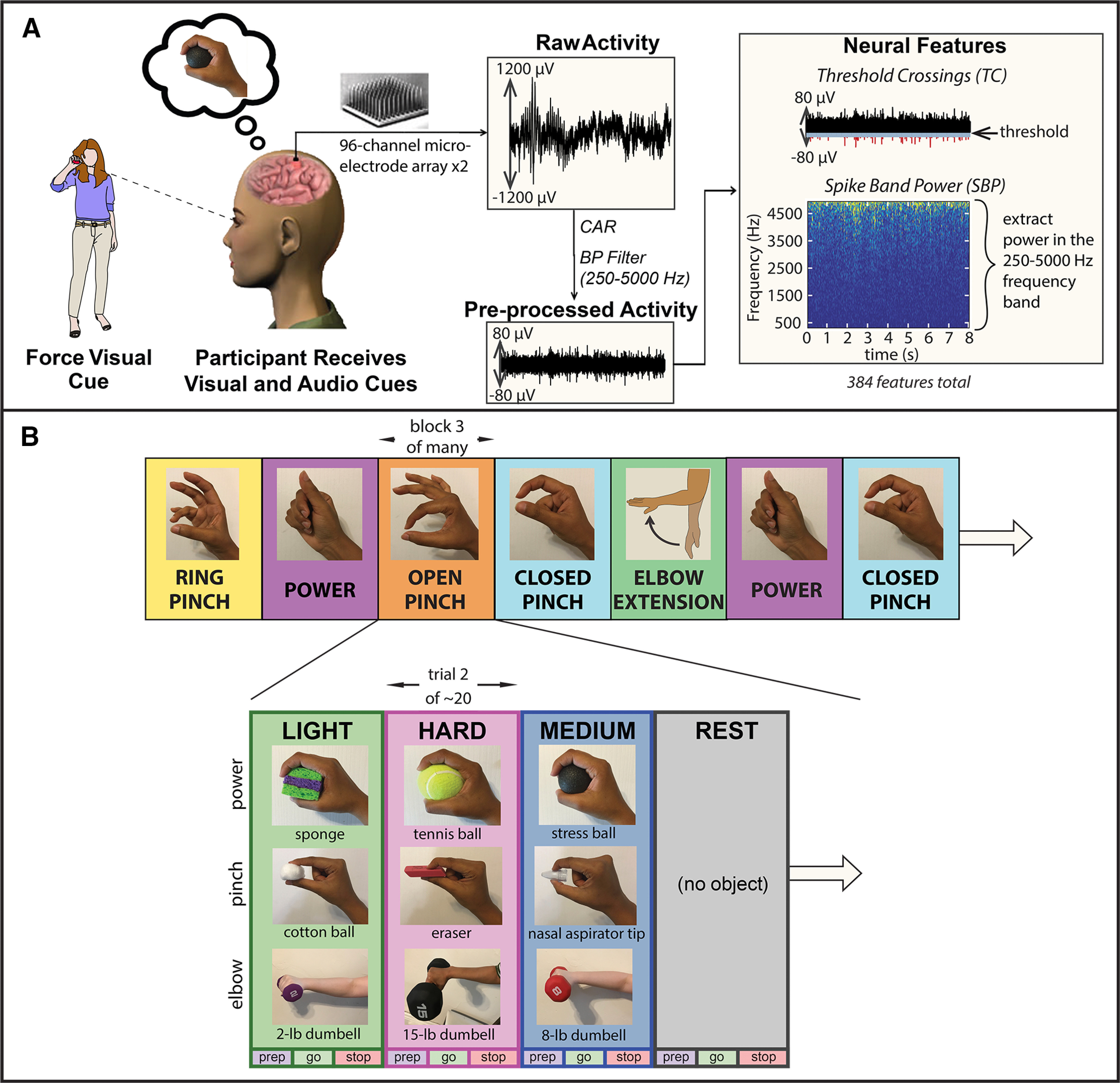
Data collection scheme for research sessions. ***A***, Experimental setup (adapted from Rastogi et al., 2020). Participants had two 96-channel microelectrode arrays placed chronically in motor cortex, which recorded neural activity while participants completed a force task. TC and SBP features were extracted from these recordings. Figure 1*A* is reprinted by permission from Springer Nature as indicated in the Terms and Conditions of a Creative Commons Attribution 4.0 International license (https://www.nature.com/srep/). ***B***, Research session architecture. Each session consisted of 12–21 blocks, each of which contained ∼20 trials (see Table 1). In each trial, participants attempted to generate one of three visually-cued forces with one of four grasps: power, closed pinch, open pinch, ring pinch. During session 5, participant T8 also attempted force production using elbow extension. Each trial contained a preparatory (prep) phase, a go phase where forces were actively embodied, and a stop phase where neural activity was allowed to return to baseline. Participants were prompted with both audio and visual cues, in which a researcher squeezed or lifted an object associated with each force level. During pinch blocks, the researcher squeezed the pinchable objects (cotton ball, eraser, nasal aspirator tip) using the particular pinch grip dictated by the block (ring pinch, open pinch, closed pinch). Here, only closed pinches of objects are shown.

Each research session consisted of multiple 4-min data collection blocks, which were each assigned to a particular hand grasp or elbow movement, as illustrated in Figure 1*B*. Blocks were presented in a pseudorandom order, in which hand grasps were assigned randomly to each set of two (session 1), four (sessions 2–4, 6–7), or five (session 5) blocks. This allowed for an equal number of blocks per hand grasp, distributed evenly across the entire research session.

All blocks consisted of ∼20 trials, which were presented in a pseudorandom order by repeatedly cycling through a complete, randomized set of force levels until the end of the block. During each trial, participants used kinesthetic imagery (Stevens, 2005; Mizuguchi et al., 2017) to internally emulate one of three discrete force levels, or rest, with the dominant hand. Participants received simultaneous audio and visual cues indicating which force to produce, when to produce it, and when to relax. Participants were visually cued by observing a researcher squeeze one of nine graspable objects corresponding to light, medium, and hard forces (no object was squeezed during “rest” trials), as shown in Figure 1*B*. The participants were asked to “follow along” and attempt the same movements that the researcher was demonstrating. The graspable objects were grouped into three sets of three, corresponding to forces embodied using a power grasp (sponge = light, stress ball = medium, tennis ball = hard), a pincer grasp (cotton ball = light, nasal aspirator tip = medium, eraser = hard), or elbow extension (5-lb dumbbell = light, 10-lb dumbbell = medium, 15-lb dumbbell = hard). These visual cues, which were included to make the concept of light, medium, and hard forces seem less abstract to participants after years of deafferentation, were deemed unlikely to be reflected within the go-phase neural response based on previous investigations (Rastogi et al., 2020). Objects were chosen to be of similar weight, size, and shape to minimize the effects of visual confounds within the neural data.

During the prep phase, which lasted a pseudo-randomly determined period between 2.7 and 3.3 s to reduce confounding effects from anticipatory activity, the researcher presented an object indicating the force level to be attempted. The researcher then squeezed the object (or lifted the object, in the case of elbow extension) during the go phase (3–5 s), and finally released the object at the beginning of the stop phase (5 s). When squeezing (or lifting) objects, the researcher used the grasp type dictated by the block. For example, to visually cue hard forces, the researcher used a ring pinch to squeeze the eraser during ring pinch blocks, but used an open pinch grasp to squeeze the eraser during open pinch blocks.

### Neural recordings

#### Preprocessing

In both participants, each intracortical microelectrode array was attached to a percutaneous pedestal connector on the head. A Blackrock shielded Patient Cable connected the pedestals to front-end amplifiers and a NeuroPort System (Blackrock Microsystems) that bandpass filtered (0.3 Hz to 7.5 kHz) and digitized (30 kHz) the neural signals from each channel on the microelectrode array. These digitized signals were preprocessed in Simulink using the xPC real-time operating system (The MathWorks Inc.). Each channel was bandpass (BP) filtered (250–5000 Hz), common average referenced (CAR), and down-sampled to 15 kHz in real time. CAR was implemented by selecting 60 channels from each microelectrode array that exhibited the lowest variance, and then averaging these channels together to yield an array-specific CAR. This reference signal was subtracted from the signals from all channels within each of the arrays.

#### Extraction of neural features

From each filtered, CAR channel, two neural features were extracted in real time using the xPC operating system from non-overlapping 20-ms time bins. These features, as illustrated in Figure 1*A*, included unsorted threshold crossing (TC) rate and spike band power (SBP) features. Each TC feature, which was equivalent to multiunit activity (Stark and Abeles, 2007) was defined as the number of times a channel’s recorded voltage time series crossed a predefined noise threshold [−4.5 × root mean square (RMS) voltage], divided by the width of the time bin (Christie et al., 2015) . The RMS voltage on each channel was calculated from 1 min of neural data recorded at the beginning of each research session. Additionally, each SBP feature was computed as the average signal power of the spike band (250–5000 Hz) within each time bin. Thus, SBP features were computed in the same manner as local field potentials (LFPs) and EEG signal power bands.

These calculations yielded 384 neural features per participant, which were used for offline analysis without spike sorting (Trautmann et al., 2019). TC features were labeled from 1 to 192 according to the recording electrodes from which they were extracted. Corresponding SBP features were labeled from 193 to 384. All features were normalized by subtracting the block-specific mean activity of the features within each recording block, to minimize non-stationarities in the data.

Unless otherwise stated, all subsequent offline analyses of neural data were performed using MATLAB software within a Windows 64-bit operating system.

### Characterization of individual neural feature tuning

The first goal of this study was to determine the degree to which force-related and grasp-related information are represented within individual TC and SBP neural features. Specifically, neural activity resulting from three discrete forces and two (session 1), four (sessions 2–4, 6–7), or five (session 5) grasps, resulted in 6, 12, or 15 conditions of interest per session, respectively. See Table 1 for a list of grasps included for each individual research session. To visualize individual feature responses to force and grasp, each feature’s peristimulus time histogram (PSTH) was computed for each of these conditions by averaging the neural activity over go-cue-aligned trials. These trials were temporally smoothed with a Gaussian kernel (100-ms SD) to aid in visualization.

To determine how many of these individual features were tuned to force and/or grasp, statistical analyses were implemented in MATLAB and with the WRS2 library in the R programming language (Wilcox, 2017), as in Rastogi et al. (2020). Briefly, features were preprocessed in MATLAB to compute each feature’s mean go-phase deviation from baseline during each trial. Baseline activity was computed by averaging neural activity across multiple rest trials.

In R, the distribution of go-phase neural deviations was found to be normal (analysis of Q-Q plots and Shapiro–Wilk tests, *p* < 0.05) but heteroskedastic (Levene’s test, *p* < 0.05), necessitating a two-way Welch ANOVA analysis to determine neural tuning to force, grasp, and their interaction (*p* < 0.05). Features exhibiting an interaction between force and grasp were further separated into individual grasp conditions (closed pinch, open pinch, ring pinch, power, elbow), within which one-way Welch-ANOVA tests were implemented to find interacting features that were tuned to force. All *p* values were corrected for multiple comparisons using the Benjamini–Hochberg procedure (Benjamini and Hochberg, 1995).

### Neural population analysis and decoding

The second goal of this study was to determine the degree to which force and grasp are represented within, and can be decoded from, the neural population. Here, the neural population was represented using both traditional and demixed principal component analysis (dPCA).

#### Visualizing force representation with traditional PCA

In order to visualize how consistently forces were represented across different grasps, neural activity collected during individual sessions were graphically represented within a low-dimensional space found using PCA. Notably, during session 5, participant T8 attempted to produce three discrete forces not only with several grasps, but also with an elbow extension movement. Therefore, two sets of PCA analyses were implemented on the data. The first, which was applied to all sessions, performed PCA on all force and grasp conditions within the session. In the second analysis specific to session 5 only, PCA was applied solely on power grasping and elbow extension trials to elucidate whether forces were represented in a consistent way across the entire upper limb. For both analyses, the PCA algorithm was applied to neural feature activity that was averaged over multiple trials and across the go phase of the task.

The results of each decomposition were plotted in a low-dimensional space defined by the first two principal components (PCs). The force axis within this space, given by Equation 1, was estimated by applying multiclass linear discriminant analysis (LDA; Juric, 2020) to the centered, force-labeled PCA data, and then using the largest LDA eigenvector as the multidimensional slope ***m*** of the force axis. Here, ***PC_score_*** is the principal component score, or representation of the neural data in PCA space, and *f* is the intended force level. A consistent force axis across multiple grasps within PCA space would suggest that forces are represented in an abstract (and thus grasp-independent) manner.

(1) $$PC_{\mathrm{score}}=mf.$$

#### dPCA

The remainder of population-level analysis was implemented using dPCA. dPCA is a dimensionality reduction technique that, similarly to traditional PCA, compresses neural population activity into a few components that capture the majority of variance in the source data (Kobak et al., 2016). Unlike traditional PCA, which yields PCs that each capture signal variance from multiple parameters of interest, dPCA performs an ANOVA-like decomposition of data into task-dependent dimensions of neural activity. That is, the resulting dPCs are tuned to individual task parameters; thus, they are much easier to interpret than traditional PCs. Additionally, because dPCA performs an ANOVA-like decomposition of data, it serves as a population-level analog to the two-way Welch ANOVA analysis implemented on individual neural features.

Briefly, the matrix ***X*** of neural data is decomposed into trial-averaged neural activity explained by time (*t*), various task parameters (*p_1_*, *p_2_*), their interaction (*p_1_p_2_*), and noise, according to Equation 2. Next, dPCA finds separate decoder (***D***) and encoder (***E***) matrices for each marginalization *M* by minimizing the loss function *L* exhibited in Equation 3.

(2) $$X=X_{t}+X_{p_{1}}+X_{p_{2}}+X_{p_{1}p_{2}}+X_{\mathrm{noise}}=\underset{M}{\sum }X_{M}+X_{\mathrm{noise}}$$

(3) $$L=\underset{M}{\sum }X_{M}-E_{M}D_{M}X^{2}.$$

The resulting dPCs, obtained by multiplying the neural data ***X*** by the rows of each decoder matrix ***D_M_***, are, in theory, de-mixed, in that the variance explained by each component is because of a single, specific task parameter *M*. These dimensions of neural activity not only reveal population-level trends in neural data, but they can also be used to decode task parameters of interest. Critically, dPCA can be used to decode task parameters from the data while still preserving its original geometry. Thus, a single technique can be used to analyze the underlying structure of neural data as it relates to the encoding of task parameters, and to simultaneously quantify how well these parameters can be decoded for use in an iBCI system (Kobak et al., 2016).

#### Single dPCA component implementation

In the present study, the task parameters of interest were force and grasp. Here, one goal was to use variance as a metric to quantify the degree to which force and grasp were represented within the neural population as a whole. Therefore, for each research session listed in Table 1, the neural data ***X*** was temporally smoothed using a Gaussian filter (100-ms SD) and decomposed into neural activity that varied with four marginalizations ***X_M_***, as per Equation 2: time (condition independent), force, grasp, and an interaction between force and grasp. The variance that each marginalization accounted for was computed as the sum of squares of the mean-centered neural data contained within the marginalization.

An additional goal was to isolate neural components that contained useful information about force and grasp, i.e., components that would enable discrimination between individual force levels and grasp types. First, dPCA was used to reduce each of the four, 384-dimensional, mean-centered marginalizations ***X_M_*** into 20 dPCs, as described by Equation 3. This yielded 80 dPCs across all four marginalizations. Second, the variances accounted for by each of the 80 components were computed as the sum of squares. Third, the top 20 out of 80 components with the highest variance were selected as representing the majority of variance in the neural dataset and were assembled into a decoder matrix ***D***. Finally, each of these top 20 components was assigned to one of the four marginalizations of interest according to the marginalization from which it was extracted. For example, dPCs that were extracted from the force marginalization ***X_force_*** were deemed as force-tuned dPCs; those extracted from the grasp marginalization ***X_grasp_*** were deemed as grasp tuned dPCs, and those extracted from the marginalization ***X_F/G_*** representing an interaction between force and grasp were deemed as interacting dPCs.

Each dPC’s information content was further quantified in two ways. First, to assess the degree to which dPCs were demixed, each dPC’s variance was subdivided into four sources of variance corresponding to each of the four marginalizations of interest, as per Equation 2. Second, the decoder axis associated with each dPC was used as a linear classifier to decode intended parameters of interest. Specifically, each force-tuned dPC was used to decode force at every time point of the behavioral task, while each grasp-tuned dPC was used to decode grasp, but not force. Likewise, components that exhibited an interaction between force and grasp were used to decode force-grasp pairs. Condition-independent dPCs, which were tuned to time, were not used to decode force or grasp from the neural activity.

Linear classification was implemented using 100 iterations of stratified Monte Carlo leave-group-out cross-validation (Kobak et al., 2016). During each iteration, one random group of *F x G* test “pseudo-trials,” each corresponding to one of the several force-grasp conditions, was set aside during each time point (*F* = number of intended forces, *G* = number of intended grasps). Next, dPCA was implemented on the remaining trials, and the decoder axes of the resulting dPCs were used to predict the intended forces or intended grasps indicated by the test set of pseudo-trials at each time point. This was accomplished by first computing mean dPC values for each force-grasp condition, separately for each time point; projecting the *F x G* pseudo-trials onto the decoder axes of the dPCs at each time point; and then classifying the pseudo-trials according to the closest class mean (Kobak et al., 2016). The proportion of *F x G* pseudo-trials correctly classified across 100 iterations at each time point constituted a time-dependent classification accuracy. Chance performance was computed by performing 100 shuffles of all available trials, randomly assigning force or grasp conditions to the shuffled data, and then performing the same cross-validated classification procedure within each of the 100 shuffles. Classification accuracies that exceeded the upper range of chance performance were deemed significant.

#### Force and grasp decoding using multiple dPCs

Two additional goals of this study were to determine whether intended forces could be accurately predicted from neural population data and whether these predictions depended on hand grasp configuration. To this end, dPCs that were tuned to force, grasp, and an interaction between force and grasp were used to construct multidimensional force and grasp decoders within each session. Specifically, the force decoder was constructed by combining the decoding axes of force-tuned and interacting components into a single, multidimensional decoder ***D_F_***; likewise, the grasp decoder ***D_G_*** was constructed by combining the decoding axes of grasp-tuned and interacting components.

Each of these decoders was used to perform 40 runs of linear force and grasp classification for each of S research sessions per participant, implemented using the aforementioned stratified Monte Carlo leave-group-out cross-validation procedure (S = 6 for T8; S = 1 for T5). As in the single component implementation (Kobak et al., 2016), each run was accomplished in multiple steps. First, the mean values of all dPCs included within the multidimensional decoder were computed for each force-grasp condition, separately for each time point. Second, at each time point, the *F x G* pseudo-trials were projected onto the multidimensional decoder axis and classified according to the closest class mean. The proportion of test trials correctly classified at each time point over 100 iterations constituted a time-dependent force or grasp classification accuracy.

The aforementioned computations yielded 40 × S time-dependent force and grasp classification accuracies per participant. Session-averaged, time-dependent force and grasp classification accuracies were computed by averaging the performance over 240 session-runs for participant T8 (40 runs × six sessions) and 40 session-runs for participant T5 (40 runs × one session). These averages were compared with chance performance, which was computed by performing 100 shuffles of all trials, randomly assigning force or grasp conditions to the shuffled data, and then performing force and grasp classification on each of the shuffled datasets using the multidimensional decoders ***D_F_*** and ***D_G_***. Time points when force or grasp classification exceeded the upper bound of chance were deemed to contain significant force-related or grasp-related information.

To visualize the degree to which individual forces and grasps could be discriminated, confusion matrices were computed over go-phase time windows during which the neural population contained significant force-related and grasp-related information. The time window began when session-averaged, time-dependent classification accuracy exceeded 90% of maximum achieved performance within the go phase, and ended at the end of the go phase. First, classification accuracies for each of the S × 40 session-runs were approximated by averaging classification performance across the prespecified go-phase time window. These time-averaged accuracies, which are henceforth referred to as mean force and grasp accuracies, were next averaged over all S × 40 session-runs to yield confusion matrix data. In this way, confusion matrices were computed to visualize force-related discriminability across all trials, force-related discriminability within individual grasp types, and grasp-related discriminability across all trials.

Classification performances for individual forces and individual grasps were statistically compared using parametric tests implemented on mean force and grasp accuracies. Specifically, for each participant, mean classification accuracies for light, medium, and hard forces were compared by implementing one-way ANOVA across mean force accuracies from all S × 40 session runs. The resulting *p* values were corrected for multiple comparisons using the Benjamini–Hochberg procedure (Benjamini and Hochberg, 1995). Likewise, mean classification accuracies for closed pinch, open pinch, ring pinch, power, and elbow “grasps” were compared by implementing one-way ANOVA across all mean grasp accuracies. These comparisons were implemented to determine whether offline force and grasp decoding yielded similar versus different classification results across multiple forces and multiple grasps.

Statistical analysis was also used to determine the degree to which grasp affected force decoding accuracy. This was achieved by implementing two-way ANOVA on mean force accuracies that were labeled with the grasps used to emulate these forces. The results of the two-way ANOVA showed a statistically significant interaction between force and grasp. Therefore, the presence of simple main effects was assessed within each force level and within each grasp type. Specifically, one-way ANOVA was implemented on mean accuracies within individual force levels to determine whether light forces, for example, were classified with similar degrees of accuracies across all grasp types. Similarly, one-way ANOVA was implemented on mean accuracies within individual grasps to see whether intended forces affected grasp classification accuracy; *p* values resulting from these analyses were corrected for multiple comparisons using the Benjamini–Hochberg procedure.

Finally, this study evaluated how well dPCA force decoders could generalize to novel grasp datasets in T8 session 5 and T5 session 7. Specifically, within each session, a multidimensional force decoder ***D_F_*** was trained on neural data generated during all but one grasp type, and then its performance was evaluated on the attempted forces emulated using the left-out “novel” grasp. To establish the generalizability of force decoding performance across many novel grasps, this analysis cycled through all available grasps attempted during session 5 (closed pinch, open pinch, ring pinch, power, elbow extension) and session 7 (closed pinch, open pinch, ring pinch, power). For each novel grasp, the trained decoder ***D_F_*** was used to perform 40 runs of stratified Monte Carlo leave-group-out cross-validated linear force classification on two sets of test data: the “initial grasp” dataset, which originated from the grasps on which the force decoder was trained; the novel grasp dataset, which originated from the leave-out test grasp. The resulting time-dependent, “initial grasp” and “novel grasp” decoding performances from the go-phase time window during above-90% maximum classification accuracy were averaged over 40 runs, and then compared using a standard *t* test. *P* values resulting from the statistical analysis were corrected for multiple comparisons across forces and test grasps using the Benjamini–Hochberg procedure.

### Comparison of force encoding models

The overarching goal of this study, which is to determine the extent to which force representation within motor cortex depends on grasp, arose from two conflicting hypotheses indicating that force representation is either grasp-independent (Chib et al., 2009; Hendrix et al., 2009; Pistohl et al., 2012; Intveld et al., 2018) or grasp-dependent (Hepp-Reymond et al., 1999; Degenhart et al., 2011). The grasp-independent and grasp-dependent force encoding hypotheses can be mathematically modeled as per Equations 4, 5, respectively:

(4) $$x_{\mathrm{ij}}=ag_{i}+bfs_{j}+d$$

(5) $$x_{\mathrm{ij}}=cs_{j}g_{i}+d$$

In these equations, ***x_ij_*** is an *N* × *T* × *TR* matrix of neural activity generated within *N* neural features over *T* time points, during *TR* trials of a particular grasp *i* and force *j*. The term ***g_i_*** is an *N* × *T* × *TR* matrix of baseline feature activity during the grasp *i,* ***f*** is an *N* × *T* × *TR* matrix of baseline activity feature activity during force generation, and *s_j_* is a discrete scalar force level. Finally, the coefficients *a*, *b*, *c*, and *d* are constants. In Equation 4, the overall neural activity ***x_ij_*** consists of an addition of independent force-related and grasp-related terms, as is thus referred to as the additive model of force encoding. In contrast, Equation 5 models the neural activity ***x_ij_*** as a multiplication of the force level *s_j_* with baseline grasp activity *g_i_*, and is hence referred to as the scalar model of force encoding.

An additional model, indicated by Equation 6, incorporates terms from both the additive and scalar models of force encoding, and is thus referred to as the combined model:

(6) $$x_{\mathrm{ij}}=ag_{i}+bfs_{j}+cs_{j}g_{i}+d$$

The additive (grasp-independent) and scalar (grasp-dependent) hypotheses of force encoding were graphically illustrated with a toy example of expected grasp-independent versus grasp-dependent (interacting) representations of force within the neural space. In the toy example, the model coefficients *a*, *b*, and *c* were set to one, and the model coefficient *d* was set to zero. The neural activity ***x_ij_*** was a vector of trial-averaged activity from 100 simulated neural features during a single time point, generated during a particular grasp *i* and force *j*. The variable ***g_i_*** was a 100 × 1 vector of normalized baseline feature activity during the grasp *i*, ***f*** was a 100 × 1 vector of normalized baseline neural feature activity during force generation, and *s_j_* was a discrete, scalar force level (1, 2, or 5). The vectors ***g_i_*** and ***f*** contained values drawn from the standard normal distribution.

Additionally, cross-validated ordinary least squares regression was used to quantify the degree to which the additive, scalar, and combined models explained the neural data ***x_ij_*** recorded from participants T8 and T5. Here, the neural data ***x_ij_*** consisted solely of force, grasp, and interacting components; condition-independent components of ***x_ij_*** were omitted. Thus, the matrix ***x_ij_*** was computed by compressing the 384-dimensional neural feature data using the dPCA decoder matrix ***D***, eliminating CI-tuned dPCs, and then transforming the data back to feature space using the encoder matrix ***E*** (see Eq. 3). Baseline grasp activity ***g_i_*** was estimated by isolating grasp-tuned components from the neural data, transforming these components back to feature space using the encoder matrix ***E_grasp_***, and then averaging the resulting activity over force conditions. Similarly, the baseline force activity ***f*** was estimated by isolating force-tuned dPCs, transforming these components back to feature space using the encoder matrix ***E_force_***, and then averaging the resulting data over all force-grasp conditions. All three neural activity variables consisted of 384 × *T* × *TR* matrices, where *T* was the number of go-phase time points and *TR* was the number of trials emulated with an individual force-grasp combination. As in the toy example, *s_j_* was a discrete scalar force level (1, 2, or 5).

Cross-validated regression analysis for each model was performed using 100 iterations of a stratified Monte Carlo leave-group-out scheme. Notably, the regression was performed on the data ***x*** generated during all combinations of forces and grasps, as opposed to ***x_ij_***, generated during a particular grasp *i* and force *j*. During each iteration of cross-validation, one random group of *F x G* pseudo-trials, each corresponding to one of the several force-grasp conditions, was set aside as a test dataset. Next, model coefficients were trained via ordinary least squares regression on the remaining data. Finally, the trained model was used to predict the neural activity generated during the emulated pseudo-trials, resulting in an *R*^2^ value for each iteration. The distributions of *R*^2^ values generated from each model were statistically compared by implementing a multiple comparisons test (Tukey method) on the results of a one-way ANOVA analysis.

### Data and code accessibility

This study made use of several computational algorithms implemented using publicly available source code packages. Code for the WRS2 R package, which was used to characterize single features, is available at https://CRAN.R-project.org/package=WRS2. Source code for the dPCA algorithm can be implemented either in MATLAB or Python and is available at https://github.com/machenslab/dPCA. The dPCA source code was modified to perform multidimensional decoding of force and grasp; these modified scripts can be made available on reasonable request by contacting the lead or senior authors. Finally, MATLAB code for the multiclass LDA algorithm used to compute low-dimensional force axes within PCA space is available on the MATLAB file exchange at https://www.mathworks.com/matlabcentral/fileexchange/31760-multiclass-lda.

The data presented in this study can be made available on reasonable request by contacting the lead or senior authors.

## Results

### Characterization of individual neural features

**Figure 2.**
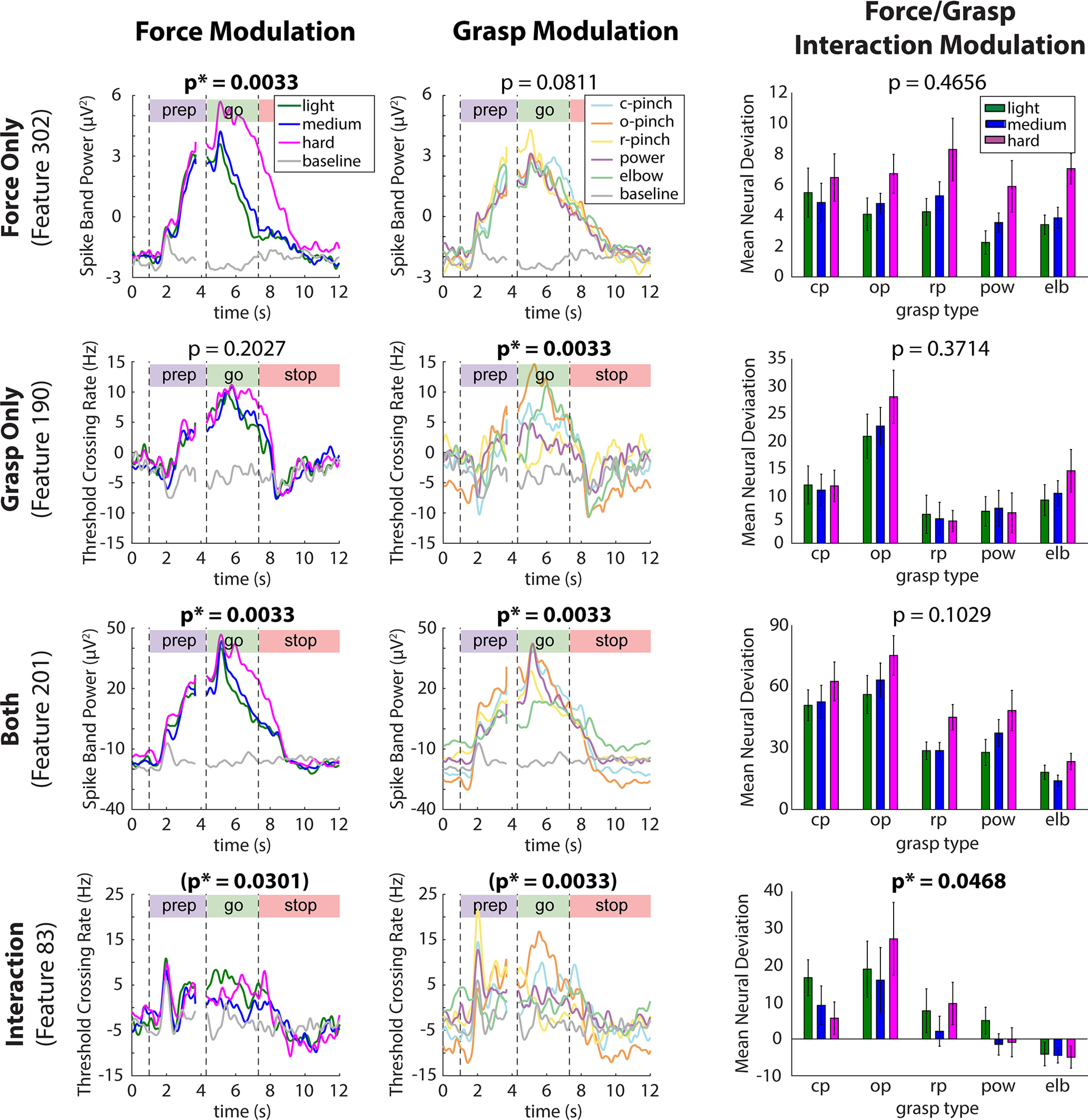
Exemplary TC and SBP features tuned to task parameters of interest in participant T8 (TC and SBP features in participant T5 are illustrated in Extended Data Fig. 2-1). Rows indicate average per-condition activity (PSTH) of four exemplary features tuned to force, grasp, both factors, and an interaction between force and grasp, recorded during session 5 from participant T8 (two-way Welch-ANOVA, corrected *p* < 0.05, Benjamini–Hochberg method). Bolded, starred p values indicate significant tuning to force (Rows 1 and 3), grasp (Rows 2 and 3), or a force-grasp interaction (Row 4). Neural activity was normalized by subtracting block-specific mean feature activity within each recording block, and then smoothed with a 100-ms Gaussian kernel to aid in visualization. Column 1 contains PSTHs averaged within individual force levels (across multiple grasps), such that observable differences between data traces are because of force alone. Similarly, column 2 shows PSTHs averaged within individual grasps (across multiple forces). Column 3 shows a graphical representation of the simple main effects as normalized mean neural deviations from baseline activity during force trials within each of the five grasps. (cp, c-pinch = closed pinch; op, o-pinch = open pinch; rp, r-pinch = ring pinch, pow = power, elb = elbow extension). Mean neural deviations were computed over the go phase of each trial and subsequently averaged within each force-grasp pair. Error bars indicate 95% confidence intervals.

Figure 2 shows the activity of four exemplary features from session 5 chosen to illustrate tuning to force, grasp, both force and grasp independently, and an interaction between force and grasp, as evaluated with two-way Welch-ANOVA (corrected *p* < 0.05, Benjamini–Hochberg procedure). These features demonstrate neural modulation to forces that T8 attempted to produce using all five grasp conditions: closed pinch, open pinch, ring pinch, power grasp, and elbow extension. Extended Data Figure 2-1 shows the activity of four additional features from participant T5. TC features are labeled from 1 to 192 according to the recording electrodes from which they were extracted. Corresponding SBP features are labeled from 193 to 384.

10.1523/ENEURO.0231-20.2020.f2-1 Extended Data Figure 2-1 Exemplary TC and SBP features tuned to task parameters of interest in participant T5, presented as in Figure 2. Note the presence of sharp activity peaks during the prep and stop phases of the trial, which were due to the presence of visual cues (Rastogi et al, 2020). Download Figure 2-1, TIF file.

For each feature, column 1 shows neural activity that was averaged across grasp types (within force levels), resulting in trial-averaged feature traces whose differences in modulation were due to force alone. Similarly, column 2 shows neural activity averaged within individual hand grasps. Here, SBP feature 302 exhibits modulation to force only (row 1), as indicated by statistically significant go-phase differentiation in activity across multiple force levels, but not across multiple grasp levels. This force-only tuning is what might be expected for a “high-level” coding of force that is independent of grasp type. Similarly, TC feature 190 is statistically tuned to grasp only, in that it exhibits go-phase differentiation across multiple grasps, but not across multiple forces. SBP feature 201, in which multiple forces and multiple grasps are statistically discriminable, is tuned to *both* force and grasp.

Figure 2, column 3, displays a graphical representation of the simple main effects of the two-way Welch-ANOVA analysis, as shown by mean go-phase neural deviations from baseline feature activity during the production of each individual force level using each individual grasp type. Here, SBP features 302 and 201, which were both tuned to force independent of grasp, showed similar patterns in modulation to light, medium, and hard forces within individual grasp types. In contrast, TC feature 83 was tuned to an interaction between force and grasp; accordingly, its modulation to light, medium, and hard forces varied according which grasp type the participant used to emulate these forces. This type of interaction is what might be expected for a more “motoric” encoding of force and grasp type. If each grasp requires a different set of muscles and joints to be active, then a motoric encoding of joint or muscle motion would end up representing force differently depending on the grasp.

**Figure 3.**
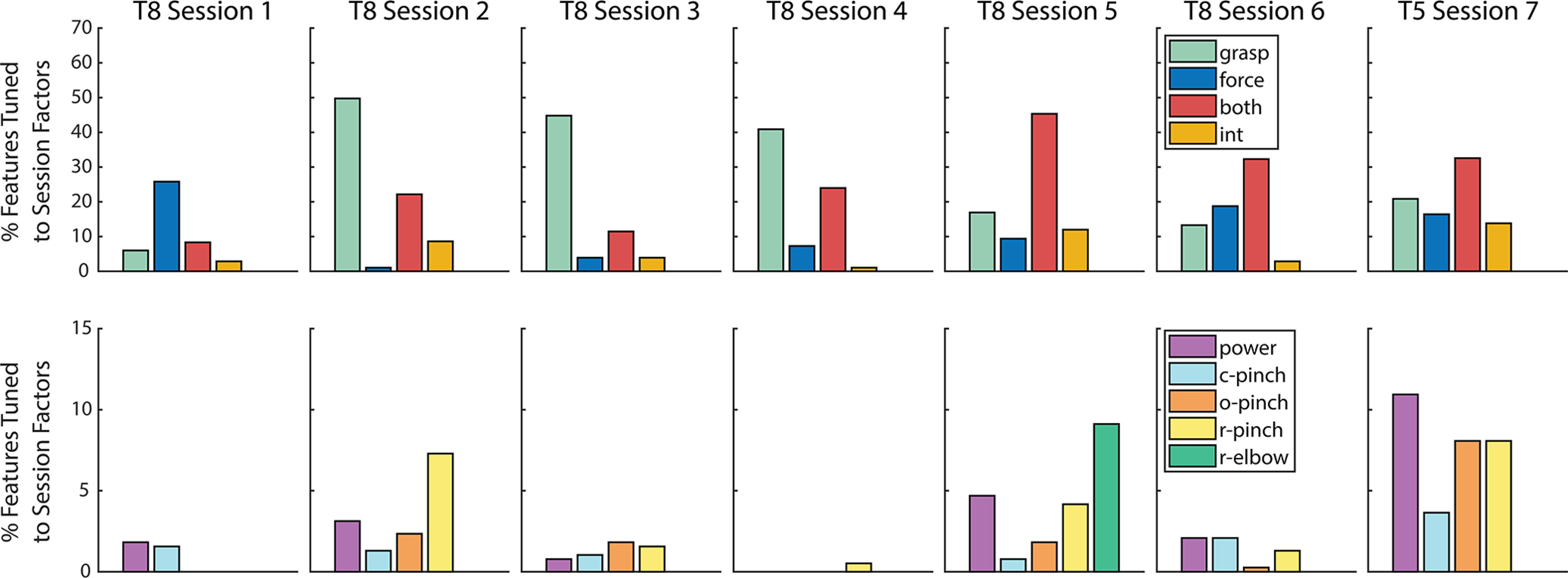
Summary of neural feature population tuning to force and grasp. Row 1, Fraction of neural features significantly tuned to force, grasp, both force, and grasp and an interaction between force and grasp in participants T8 and T5 (two-way Welch-ANOVA, corrected *p* < 0.05). Row 2, Fraction of neural features significantly tuned to an interaction between force and grasp, subdivided into force-tuned features within each individual grasp (c-pinch = closed pinch, o-pinch = open pinch, r-pinch = ring pinch). Note that the number of grasp types differed between sessions (see Table 1).

Figure 3 summarizes the tuning properties of all 384 TC and SBP neural features in participants T8 and T5, as evaluated with robust two-way Welch-ANOVA. Specifically, Figure 3*A* shows the fraction of neural features tuned to force, grasp, both force and grasp, and an interaction between force and grasp. Features belonging to the former three groups (i.e., those that exhibited no interactions between force and grasp tuning) were deemed as independently tuned to force and/or grasp. As shown in row 1, the proportion of features belonging to each of these groups varied considerably across experimental sessions. However, during all sessions in both participants, a substantial proportion of features (ranging from 15.4% to 54.7% of the feature population across sessions) were tuned to force, independent of grasp. In other words, a substantial portion of the measured neural population represented force and grasp independently.

A smaller subset of features exhibited an interaction between force and grasp in both T8 (5.2 ± 4.2%) and T5 (13.8%). Figure 3, row 2, further separates these interacting features into those that exhibited force tuning within each individual grasp type, as evaluated by one-way Welch-ANOVA (corrected *p* < 0.05). Here, the proportion of interacting features tuned to force appeared to depend on grasp type, particularly during sessions 2, 4, 5, 6, and 7, in a session-specific manner. In other words, within a small contingent of the neural feature population, force representation showed some dependence on intended grasp. Taken together, Figure 3 suggests that force and grasp are represented both independently and dependently within motor cortex at the level of individual neural features.

### Neural population analysis and decoding

#### Simulated force encoding models

**Figure 4.**
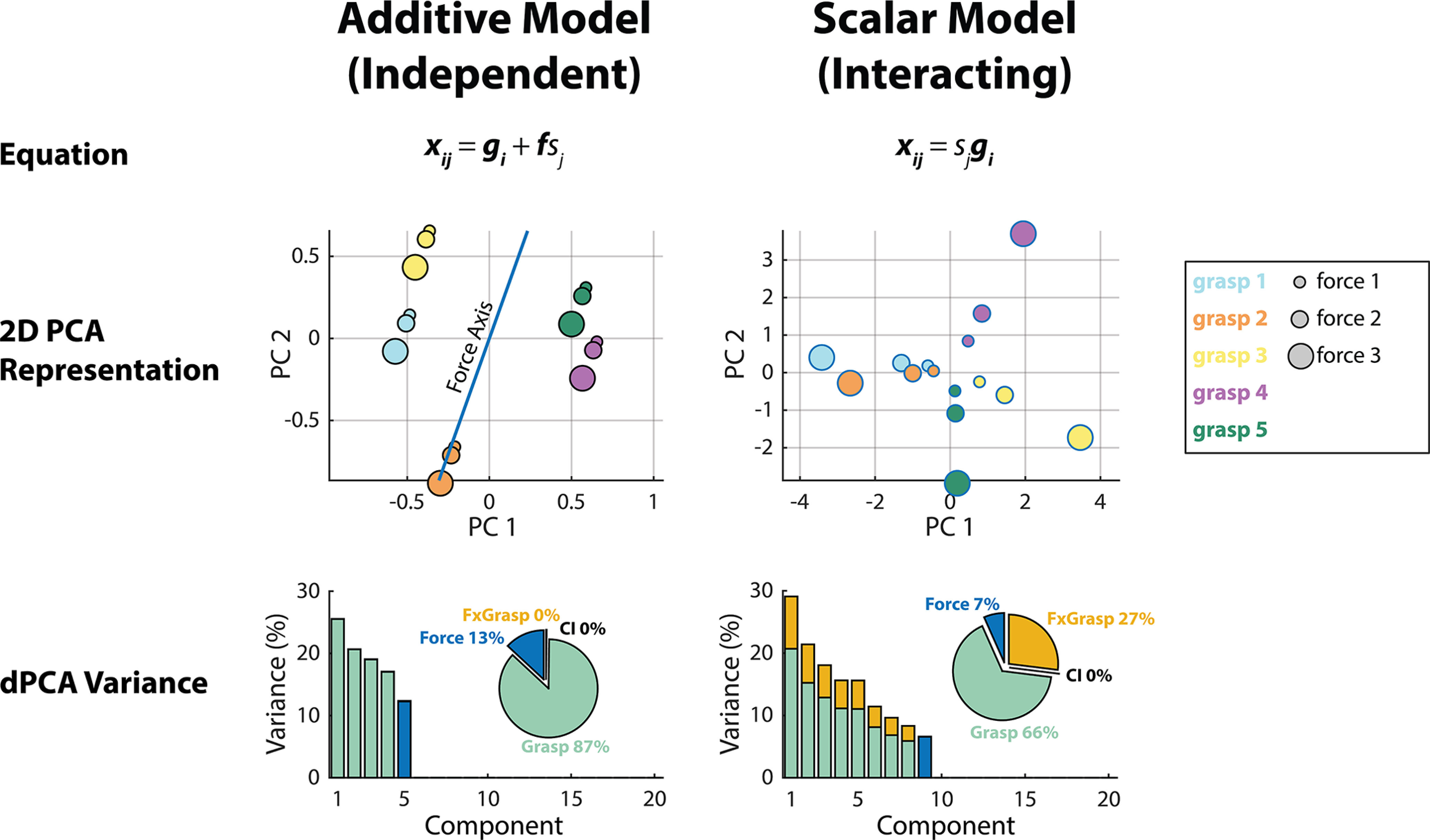
Simulated models of independent and interacting (grasp-dependent) neural representations of force. Row 1, Equations corresponding to the independent and interacting models of force representation. Here, ***x_ij_*** represents neural feature activity generated during a particular grasp *i* and force *j*, *g_i_* represents baseline feature activity during grasp *i, f* represents force-related neural feature activity, and *s_j_* is a discrete force level. Row 2, Simulated population neural activity projected into a two-dimensional PCA space. Estimated force axes within the low-dimensional spaces are shown as blue lines. Row 3, Summary of variances accounted for by the top 20 dPCs extracted from the simulated neural data from each model. Here, the variance of each individual component is separated by marginalization (force, grasp, and interaction between force and grasp). Pie charts indicate the percentage of total signal variance due to these marginalizations.

The goal of this study was to clarify the degree to which hand grasps affect neural force representation and decoding performance, in light of conflicting evidence of grasp-independent (Chib et al., 2009; Hendrix et al., 2009; Pistohl et al., 2012; Intveld et al., 2018) versus grasp-dependent (Hepp-Reymond et al., 1999; Degenhart et al., 2011) force representation in the literature. Before visualizing population-level representation of force, we first illustrate these differing hypotheses with a toy example of expected grasp-independent versus grasp-dependent (interacting) representations of force within the neural space. Figure 4 simulates grasp-independent force encoding with an additive model (Eq. 4), and grasp-dependent force encoding with a scalar model (Eq. 5; reproduced in Fig. 4, row 1).

Within the additive model, the overall neural activity ***x_ij_*** generated during a grasp *i* and force *j* is represented as a summation of independent force-related and grasp-related contributions. Thus, the additive model simulates independent neural force representation, in which force is represented at a high level independent of grasp. In contrast, the scalar encoding model simulates the neural activity ***x_ij_*** as resulting from a multiplication of the force level *s_j_* and the baseline grasping activity ***g_i_***. Such an effect might be expected if force were encoded as low-level tuning to muscle activity. In this case, different force levels would result in the same pattern of muscle activity being activated to a lesser or greater degree, thus scaling the neural activity associated with that grasp, resulting in a coupling between force and grasp. Therefore, the scalar model simulates an interacting (grasp-dependent) neural force representation.

Figure 4, row 2, shows simulated neural activity resulting from the additive and scalar encoding models within two-dimensional PCA space. In the independent model, force is represented in a consistent way across multiple simulated grasps, as indicated by the force axis. In contrast, within the interacting model, force representation differs according to grasp. These differences are further highlighted in Figure 4, row 3, in which dPCA was applied to the simulated neural data (over 20 simulated trials) resulting from each model. While the additive model exhibited no interaction-related neural variance, the scalar model yielded a substantial proportion of force, grasp, and interaction-related variance. Note that within these toy models, the simulated neural activity did not vary over its time course and, thus, exhibited no condition-independent (time-related) variance.

#### Neural population analysis

**Figure 5.**
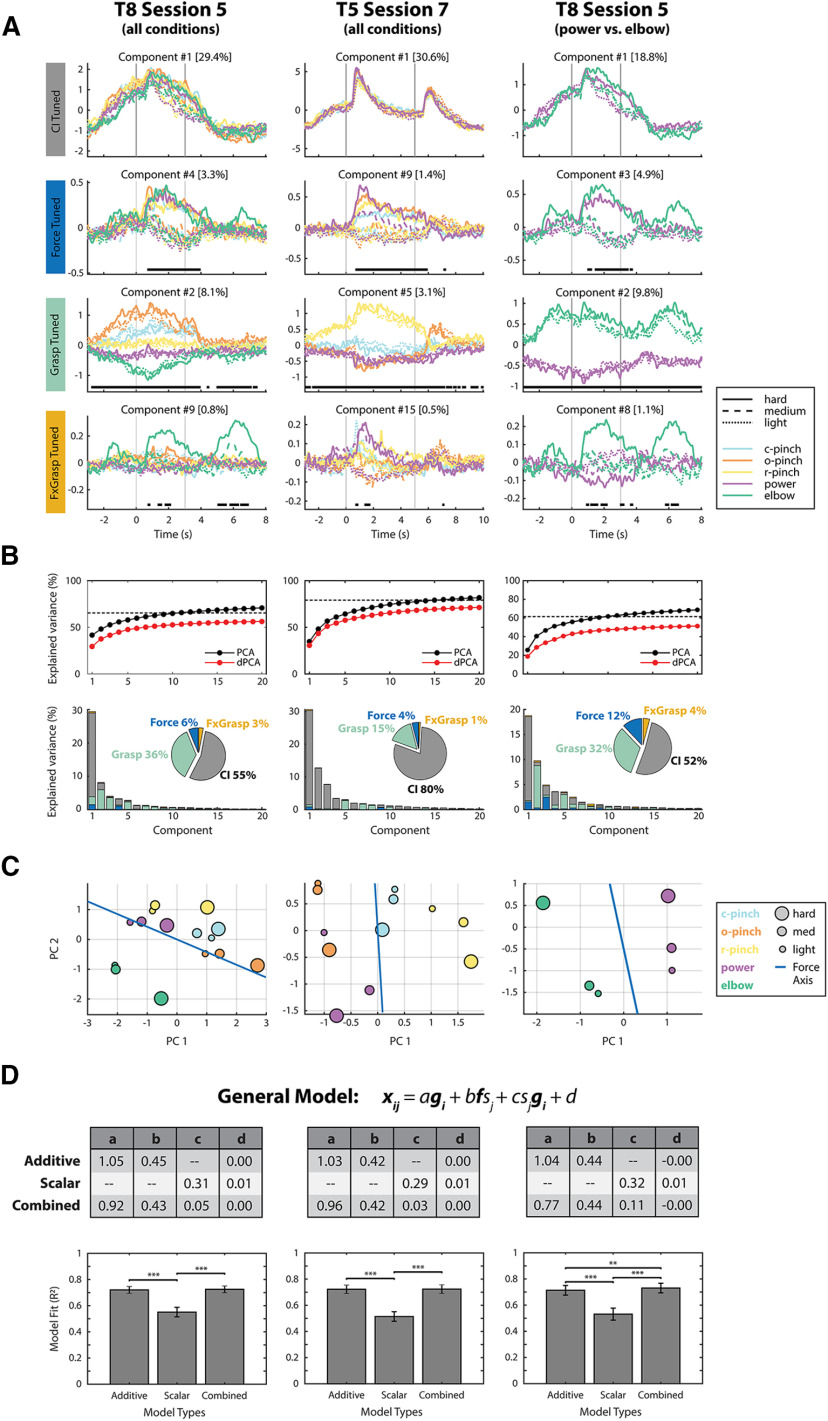
Neural population-level activity patterns. ***A***, Demixed principal components (dPCs) isolated from all force-grasp conditions from T8 session 5, all force-grasp conditions from T5 session 7, and power versus elbow conditions from T8 session 5 neural data. dPCs were tuned to four marginalizations of interest: Condition-Independent (CI) tuning (i.e., time), Force, Grasp, and an interaction between force and grasp (FxGrasp). dPCs that account for the highest amount of variance in the per-marginalization neural activity are shown here. These variances are included in brackets next to each component number. Vertical bars indicate the start and end of the go phase. Horizontal bars indicate time points at which the decoder axes of the pictured components classified forces (row 2), grasps (row 3), or force-grasp pairs (row 4) significantly above chance. ***B***, Summary of variances accounted for by the top 20 dPCs and PCs from each session. Here, the variance accounted for by the dPCs approaches the variance accounted for by traditional PCs. Horizontal dashed lines indicate total signal variance, excluding noise. Row 2 shows the variance of each individual component, separated by marginalization. ***C***, Go-phase activity within a two-dimensional PCA space. Estimated force axes within the low-dimensional PCA spaces are shown as blue lines. Here, c-pinch = closed pinch, o-pinch = open pinch, r-pinch = ring pinch. ***D***, Encoding model performances. The task-dependent components of neural feature activity were fit to the additive, scalar, and combined encoding models via cross-validated ordinary least squares regression. Tables contain the fit model coefficients for each session. Bar graphs indicate mean *R*^2^ values for each model over 100 iterations of Monte Carlo leave-group-out cross-validation. Error bars indicate SDs across iterations. Stars indicate statistically significant differences between model *R*^2^ values; **p < 0.01 and ***p < 0.001.

Figure 5 shows neural population-level activity patterns during sessions 5 and 7 from participants T8 and T5, respectively. Here, session 5 data were shown to illustrate the neural population response to forces emulated using all five grasp conditions. Additionally, session 7 data were shown as the representative dataset from participant T5. In the first two columns of Figure 5, dPCA and traditional PCA were applied to all force-grasp conditions in both participants. In the third column, these dimensionality reduction techniques were applied solely to force trials attempted using the power grasping and elbow extension, to further quantify force representation across the entire upper limb. Population-level activity patterns for additional sessions are shown in Extended Data Figures 5-1, 5-2.

10.1523/ENEURO.0231-20.2020.f5-1 Extended Data Figure 5-1 Neural population-level activity patterns for all sessions, presented as in Figure 5*A–C*. ***A***, dPCs isolated from all individual sessions of neural data. ***B***, Summary of variances accounted for by the top 20 dPCs from each exemplary session. Pie charts indicate the percentage of total signal variance accounted for by each marginalization. Total signal variance was computed with (left) and without (right) the condition-independent portion of the signal, as a basis of comparison to Figure 4 of the main text. ***C***, Go-phase activity within two-dimensional PCA space. This figure shows dPCs, variances, and PCA plots for all recorded sessions. Corresponding encoding model performances for all recorded sessions appear in Extended Data Figure 5-2. Download Figure 5-1, TIF file.

10.1523/ENEURO.0231-20.2020.f5-2 Extended Data Figure 5-2 Encoding model performances, presented as in Figure 5*D*. Download Figure 5-2, TIF file.

The 12 dPCs shown in Figure 5*A* explain the highest amount of variance within each of the four marginalizations of interest, for each participant. For example, participant T8’s component #4 (row 2, column 1) is the largest force-tuned component in the dataset and explains 3.3% of the neural data’s overall variance. Similarly, T8’s component #2 (row 3, column 1), which captures grasp-related activity, explains 8.1% of neural variance. Horizontal black bars on each panel indicate time points at which individual dPC decoding axes predict intended forces (row 2), grasps (row 3), and force-grasp pairs (row 4) more accurately than chance performance. In both participants, single components were able to offline-decode intended forces at above-chance levels solely during the active “go” phase of the trial, indicated by the vertical gray lines. However, grasp-tuned components were able to accurately predict intended grasps at nearly all time points during the trial, including the prep and stop phases. These trends were observed when dPCA was applied across all force-grasp conditions (columns 1 and 2) and across solely power and elbow trials in participant T8 (column 3).

Figure 5*B* summarizes the variance accounted for by the entire set of dPCs extracted from each dataset. Specifically, the first row shows the cumulative variance captured by the dPCs (red), as compared with components extracted with traditional PCA (black). Here, dPCs extracted from different marginalizations were not necessarily orthogonal and accounted for less cumulative variance than traditional PCs because the axes were optimized for demixing in addition to capturing maximum variance. However, the cumulative dPC variance approached total signal variance, as indicated by the dashed horizontal lines in each panel, and were thus deemed as a faithful representation of the neural population data.

Figure 5*B*, second row, further subdivides the variances of individual dPCs into per-marginalization variances. Here, most of the variance in each extracted component can be attributed to one primary marginalization, indicating that the extracted components are fairly well demixed. Pie charts indicate the percentage of total signal variance (excluding noise) from force, grasp, force/grasp interaction, and condition-independent signal components. In both participants, condition-independent components accounted for the highest amount of neural signal variance, followed by grasp, then force, then force-grasp interactions. In other words, more variance could be attributed to putative grasp representation than force representation at the level of the neural population. Additionally, force-grasp interactions only accounted for a small amount of neural variance, even when dPCA was applied solely across power grasping and elbow extension trials (column 3). Session 5 contained a larger amount of interaction variance than other sessions, possibly because of the presence of elbow extension trials that were attempted over a larger range of forces than those emulated with distal hand grasps. However, interaction variance was nonetheless smaller than force-related and grasp-related variance.

Figure 5*C* visualizes the trial-averaged, go-phase-averaged neural activity from each dataset within two-dimensional PCA space. Within these plots, each data point represents the average neural activity corresponding to an individual force-grasp condition. In all panels, light, medium, and hard forces, represented as different shapes within PCA space, are aligned to a consistent force axis (shown in blue) across multiple grasps, and also across power grasping and elbow extension movements.

Finally, Figure 5*D* quantifies how well the data can be explained by the additive (grasp-independent) and scalar (grasp-dependent) encoding models presented in Equations 4, 5 and illustrated in Figure 4. Fitted model coefficients obtained via cross-validated ordinary least squares regression are indicated within session-specific tables, while *R*^2^ values for the trained models are indicated as bar plots. Here, the additive model significantly outperformed the scalar model for all sessions (*p* < 0.001, one-way ANOVA, Tukey method). In agreement with this result, Figure 5*B*,*C* resemble the simulation results from the additive force encoding model (Fig. 4; Eq. 4), which would be expected for grasp-independent force representation. However, a small amount of interaction-related variance was also present in Figure 5*B*, and the force activity patterns in Figure 5*C* deviated to a small degree from the force axis, indicating that the additive model may not fully explain the neural activity. Therefore, the neural data were also fit to a combined model (Eq. 6), which incorporated terms from both the additive and scalar models. When fitted to neural data recorded from all force-grasp conditions (columns 1 and 2), the combined model performed similarly to the additive model (*p* > 0.05), likely because the scalar term within the combined model was assigned a low weighting coefficient *c*. However, when applied solely to the power and elbow extension trials of session 5, the combined slightly outperformed the additive model (*p* < 0.01), in agreement with the slightly larger force/grasp interaction-related variance present within this subset of the data.

#### Time-dependent decoding performance

**Figure 6.**
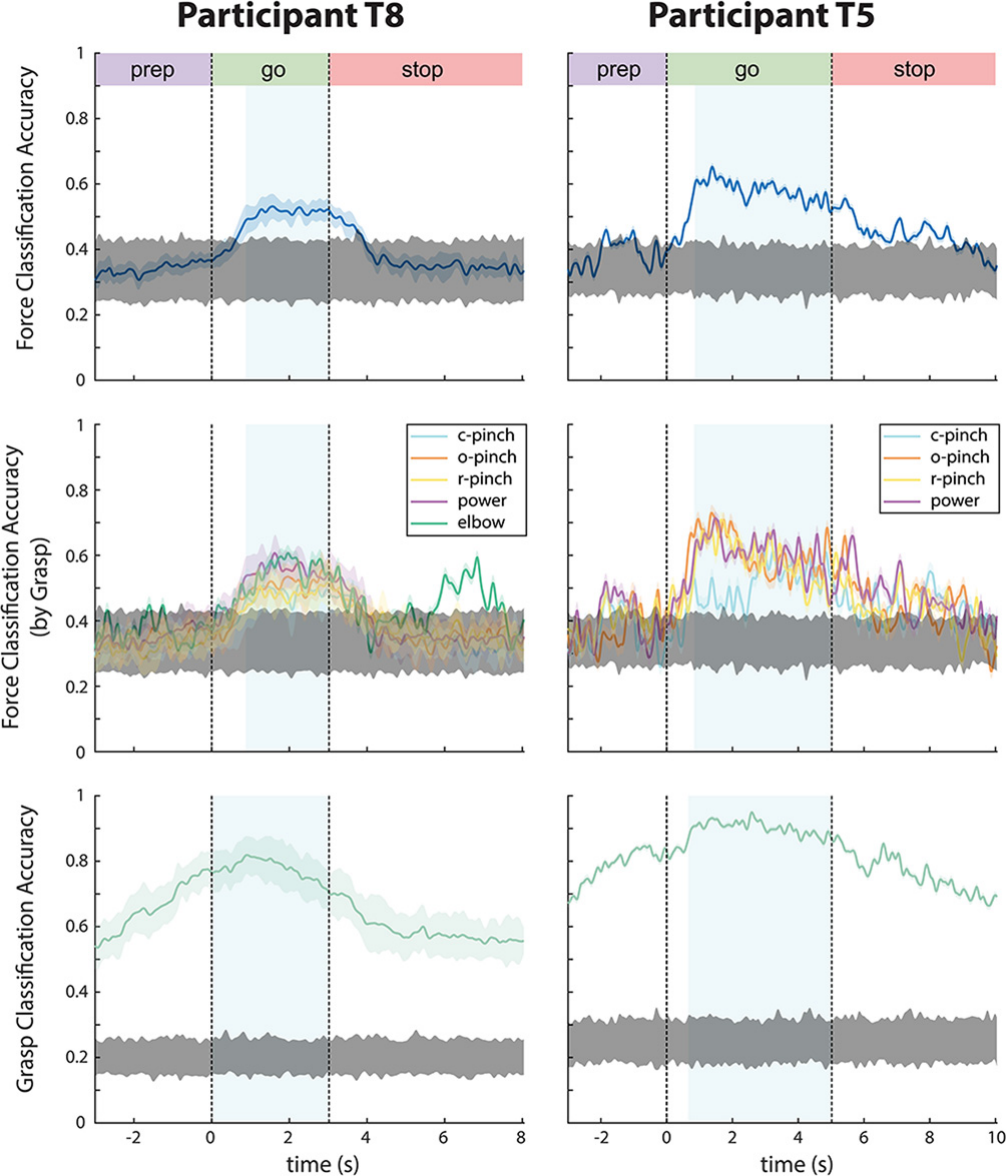
Time-dependent classification accuracies for force (rows 1–2) and grasp (row 3). Data traces were smoothed with a 100-ms boxcar filter to aid in visualization. Shaded areas surrounding each data trace indicate the SD across 240 session-runs for most trials in participant T8, 40 session-runs for elbow extension trials in participant T8, and 40 session-runs in participant T5. Gray shaded areas indicate the upper and lower bounds of chance performance over S × 100 shuffles of trial data, where S is the number of sessions per participant. Time points at which force or grasp is decoded above the upper bound of chance are deemed to contain significant force-related or grasp-related information. Blue shaded regions indicate the time points used to compute go-phase confusion matrices in Figure 7. Here, c-pinch = closed pinch, o-pinch = open pinch, r-pinch = ring pinch. Time-dependent classification accuracies for individual force levels and grasp types are shown in Extended Data Figure 6-1. Grasp classification accuracies, separated by number of attempted grasp types, are presented in Extended Data Figure 6-2. Force classification accuracies, separated by individual session, are presented in Extended Data Figure 6-3.

Figure 6 summarizes the degree to which intended forces and grasps could be predicted from the neural activity using the aforementioned dPCs. Here, offline force decoding accuracies were computed by using a force decoder ***D_F_***, created by assembling the decoding axes of multiple force-tuned and interacting components, to classify light, medium, and hard forces over multiple session-runs of a 100-fold, stratified, leave-group-out Monte Carlo cross-validation scheme, as described in the Methods. Similarly, grasp decoding accuracies in row three were computed using a grasp decoder ***D_G_***, created by assembling the decoding axes of grasp-tuned and interacting dPCs. Figure 6, row 1, shows time-dependent force decoding results, averaged over S × 40 session-runs in participants T8 (S = 6) and T5 (S = 1). Row 2 further subdivides the results of row 1 into force decoding accuracies achieved during individual hand grasps. Finally, row 3, shows time-dependent grasp decoding results for both participants.

10.1523/ENEURO.0231-20.2020.f6-1 Extended Data Figure 6-1 Time-dependent classification accuracies for individual force levels and grasp types. ***A***, Time-dependent classification accuracies for force (row 1) and grasp (row 2), separated by force class and grasp class, respectively. Data traces were smoothed with a 100-ms boxcar filter to aid in in visualization. Shaded areas surrounding each data trace indicate the SD across 240 session-runs during most trials in participant T8, 40 session-runs during elbow extension trials in participant T8, and 40-session runs in participant T5. Gray shaded regions indicate the upper and lower bounds of chance performance over S × 100 shuffles of trial data, where S is the number of sessions per participant. Blue shaded regions indicate the time points used to compute go-phase confusion matrices. ***B***, Time-dependent force classification accuracies during individual grasps in participants T8 (row 1) and T5 (row 2). Blue shaded regions indicate the time points used to compute go-phase confusion matrices. Decoding performances were averaged over S × 40 session runs, where S is the number of sessions per participant. Download Figure 6-1, TIF file.

10.1523/ENEURO.0231-20.2020.f6-2 Extended Data Figure 6-2 Time-dependent grasp classification accuracies by number of grasps attempted per session in participant T8. Data traces were smoothed with a 100-ms boxcar filter to aid in in visualization. Shaded areas surrounding each data trace indicate the SD across 40 runs during each session in participant T8. Gray shaded regions indicate the upper and lower bounds of chance performance over 100 shuffles of trial data per session. Intended grasp is classified above chance performance at all trial time points, regardless of the number of grasps to be decoded. Download Figure 6-2, TIF file.

10.1523/ENEURO.0231-20.2020.f6-3 Extended Data Figure 6-3 Time-dependent force classification accuracies by force level, per session, in participant T8. Data traces were smoothed with a 100-ms boxcar filter to aid in in visualization. Shaded areas surrounding each data trace indicate the SD across 40 runs during each session in participant T8. Gray shaded regions indicate the upper and lower bounds of chance performance over 100 shuffles of trial data per session. Intended grasp is classified above chance performance at all trial time points, regardless of the number of grasps to be decoded. Download Figure 6-3, TIF file.

Here, intended forces were decoded at levels exceeding the upper bound of chance solely during the go phase across all sessions (Extended Data Fig. 6-3), regardless of the grasp used to emulate the force. The exception to this trend occurred during elbow extension trials, in which intended forces were decoded above chance during the stop phase. In contrast, intended grasps were decoded above chance during all trial phases, regardless of the number of grasps from which the decoder discriminated (Extended Data Fig. 6-2), although go-phase grasp decoding accuracies tended to exceed those achieved during other trial phases. In summary, both intended forces and grasps were decoded above chance during time periods when participants intended to produce these forces and grasps, and in some cases, during preparatory and stop periods. Session-averaged, time dependent decoding accuracies for individual force levels and grasp types are displayed in Extended Data Figure 6-1.

#### Go-phase decoding performance

**Figure 7.**
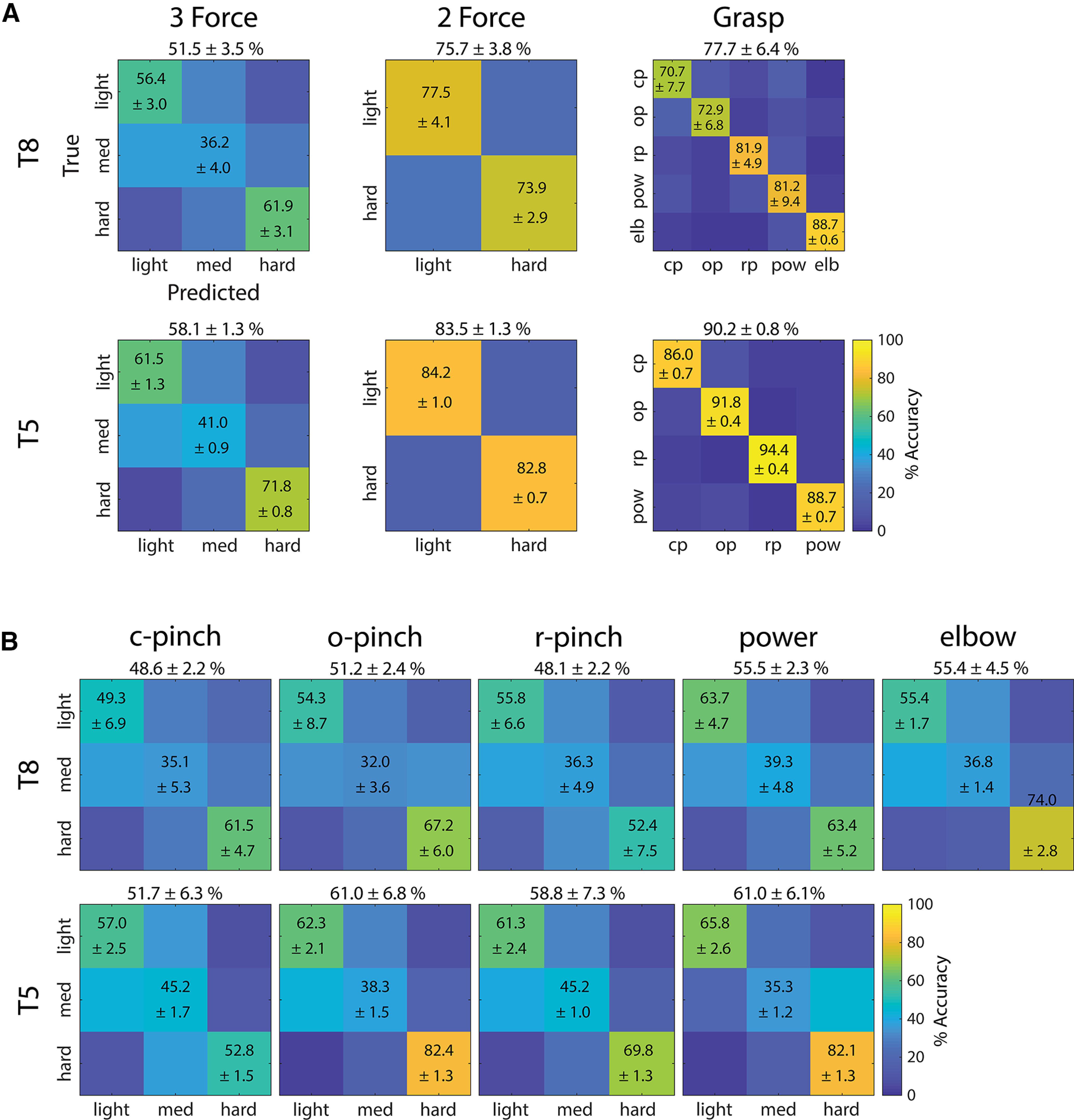
Go-phase confusion matrices. ***A***, Time-dependent classification accuracies (shown in Fig. 6) were averaged over go-phase time windows that commenced when performance exceeded 90% of maximum and ended with the end of the go phase. These yielded mean trial accuracies, which were then averaged over all session-runs in each participant. Overall force and grasp classification accuracies are indicated above each confusion matrix. SDs across multiple session-runs are indicated next to mean accuracies (cp = closed pinch, op = open pinch, rp = ring pinch, pow = power, elb = elbow extension). Statistical comparisons between the achieved classification accuracies are shown in Extended Data Figure 7-1. ***B***, Confusion matrices, now separated by the grasps (c-pinch = closed pinch, o-pinch = open pinch, r-pinch = ring pinch, power, elbow) that participants T8 (row 1) and T5 (row 2) used to attempt producing forces. Statistical comparisons between the achieved force accuracies are shown in Extended Data Figures 7-2, 7-3.

Figure 7 summarizes go-phase force and grasp decoding accuracies as confusion matrices. Here, time-dependent classification accuracies for each force level and each grasp type were averaged over go-phase time windows (see Fig. 6) that commenced when overall classification performance exceeded 90% of their maximum, and ended with the end of the go phase. This time period was selected to exclude the rise time in classification accuracy at the beginning of the go phase, so that the resulting mean trial accuracies reflected stable values. The mean trial accuracies were then averaged over all session-runs in each participant to yield confusion matrices of true versus predicted forces and grasps. Figure 7*B* further subdivides overall three-force classification accuracies into force classification accuracies achieved during each individual grasp type (columns) in both participants (rows). The confusion matrices in Figure 7 represent cumulative data across multiple sessions in participant T8, and one session in participant T5. Extended Data Figures 7-1, 7-2, 7-3 statistically compare decoding accuracies between individual force levels and grasp types within each individual session.

10.1523/ENEURO.0231-20.2020.f7-1 Extended Data Figure 7-1 Statistics for go-phase force and grasp classifications accuracies. ***A***, Force classification accuracy histograms (row 1) and corrected p values (row 2). Hard and light forces are classified significantly more accurately than medium forces across all sessions (*p* < 0.05). ***B***, Grasp classification accuracy histograms (row 1) and corrected *p* values (row 2). Decoding performance differed significantly between grasps across all sessions. Download Figure 7-1, TIF file.

10.1523/ENEURO.0231-20.2020.f7-2 Extended Data Figure 7-2 Statistics for go-phase force classification accuracies within individual grasp types. A one-way ANOVA was implemented on force classification accuracies achieved during different grasp types. ***A***, Force classification accuracy histograms. ***B***, *p* values between force pairs, corrected for multiple comparisons across grasps and sessions using the Benjamini–Hochberg procedure. Within each grasp, hard and light forces were classified more accurately than medium forces across all sessions (*p* < 0.05). Download Figure 7-2, TIF file.

In Figures 6*A*, 7*A* and Extended Data Figure 6-1, overall, three-force classification accuracies exceeded the upper limit of chance in both participants. However, the decoding accuracies of individual force levels were statistically different. For almost all sessions, hard forces were classified more accurately than light forces (with the exception of session 4, during which light and hard force classification accuracy was statistically similar), and both light and hard forces were always classified more accurately than medium forces. More specifically, hard and light forces were decoded above chance across all sessions, while medium force classification accuracies often failed to exceed chance in both participants.

In contrast, both overall and individual grasp decoding accuracies always exceeded the upper limit of chance. According to Figure 7*A* and Extended Data Figure 7-1*B*, certain grasps were decoded more accurately than others. Specifically, in participant T8, the power and ring pincer grasps were often classified more accurately than the open and closed pincer grasps across multiple sessions (corrected *p* ≪ 0.05, one-way ANOVA). Elbow extension, which required the participants to attempt force production in the upper limb in addition to the hand, was classified more accurately than any of the grasping forces during session 5 (corrected *p* ≪ 0.05). In participant T5, grasp classification accuracies, in order from greatest to least, were ring pincer > open pincer > power > closed pincer. Regardless, grasp decoding performance always exceeded force decoding performance in both participants, as seen in Figures 6, 7.

In Figure 7 and Extended Data Figure 7-3, overall and individual force classification accuracies varied depending on the hand grasps used to attempt these forces. Specifically, classification accuracies for forces attempted with different grasps were, with few exceptions, statistically different (corrected *p* ≪ 0.05, one-way ANOVA). For example, in Figure 7*B* and Extended Data Figure 7-3, hard forces attempted using the open pincer grasp were always classified more accurately than hard forces attempted using the ring pincer grasp in both participants. In other words, grasp type affected how accurately forces were decoded.

10.1523/ENEURO.0231-20.2020.f7-3 Extended Data Figure 7-3 Statistics for go-phase force classification accuracies within individual force levels. A one-way ANOVA was implemented on the force classification accuracies achieved during different grasp types. ***A***, Force classification accuracy histograms, color-coded by the grasp type used to produce each force level. ***B***, *p* values between pairs of grasps used to produce each individual force level, corrected for multiple comparisons across forces and sessions using the Benjamini–Hochberg procedure. The decoding performance for each discrete force level was significantly different across grasps (*p* < 0.05), indicating that grasp type affected force decoding performance. Download Figure 7-3, TIF file.

**Figure 8.**
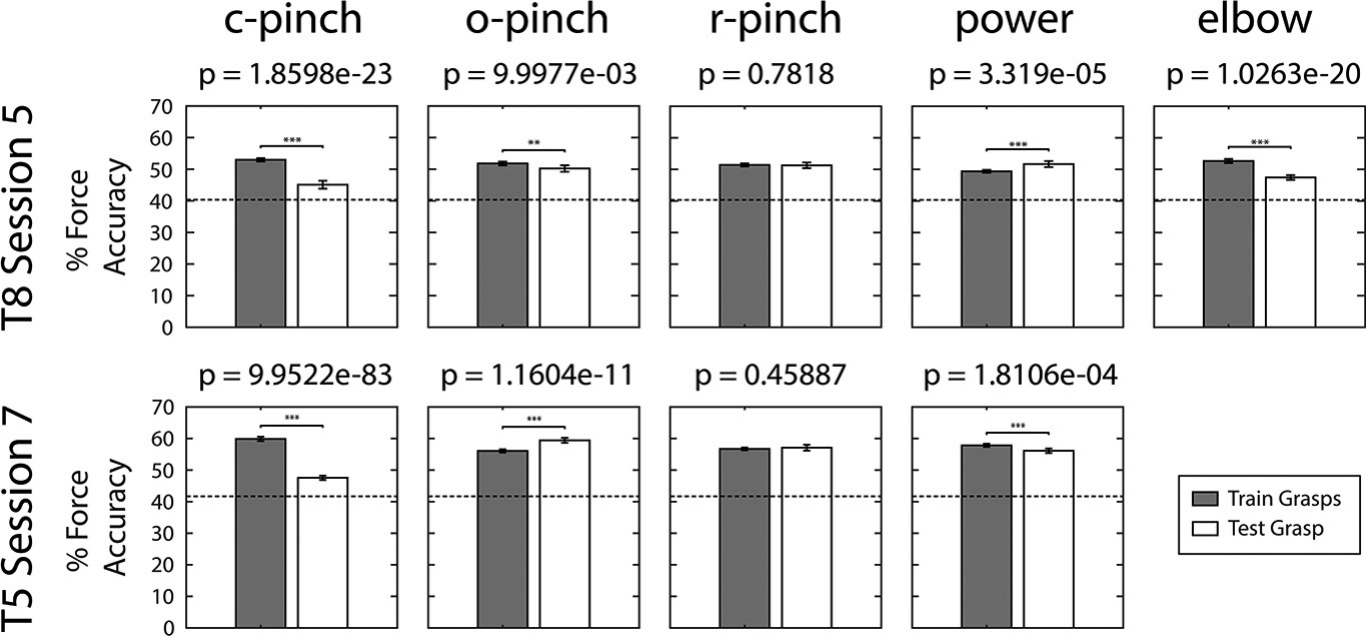
Go-phase force classification accuracy for novel (test) grasps. Within each session (rows), dPCA force decoders were trained on neural data generated during all grasps, excluding a single leave-out grasp type (columns). The force decoder was then evaluated over the set of training grasps (gray bars), as well as the novel leave-out grasp type (white bars). Stars indicate statistically significant differences in performance between training and novel grasps; **p < 0.01, ***p < 0.001. Error bars indicate the 95% confidence intervals. The horizontal dotted line in each panel indicates upper bound of the empirical chance distribution for force classification. Here, c-pinch = closed pinch, o-pinch = open pinch, r-pinch = ring pinch.

Finally, Figure 8 summarizes how well force decoders trained on one set of grasps generalized to novel grasp types in T8 session 5 (row 1) and T5 session 7 (row 2). A force decoder was used to discriminate forces among a set of grasps used for training (“left-in,” gray bars) or a leave-out novel grasp (white bars). Here, the force decoding performance between the leave-in and leave-out grasps was significantly different in seven out of nine comparisons, suggesting that grasp affects how well forces are decoded from neural activity. However, for all sets of grasps, force decoding performance always exceeded chance. This was even true when, during T8 session 5, the force decoder was trained on four hand grasps and evaluated on elbow extension data. This is consistent with the previous population-level analyses that show that components of force representation in motor cortex are conserved across grasps and even arm movements.

## Discussion

The current study sought to determine how human motor cortex encodes hand grasps and discrete forces, how much these representations interacted, and how well forces and grasps could be decoded. Three major findings emerged from this work. First, force information was present in, and could be decoded from, intracortical neural activity in a consistent way across multiple hand grasps. This suggests that force is, to some extent, represented at a high level in individuals with tetraplegia, independent of motion and grasp. However, as a second finding, grasp affected force representation and classification accuracy, suggesting a simultaneous, low-level, motoric representation of force in individuals with tetraplegia. Finally, hand grasps were classified more accurately and explained more neural variance than forces. These three findings and their implications for future online force decoding efforts are discussed here.

### Force and grasp representation in motor cortex

#### Force information persists across multiple hand grasps in individuals with tetraplegia.

##### Overall force representation

Force was represented in a consistent way across multiple hand grasps within the neural activity. In particular, a substantial contingent of neural features was tuned to force independent of grasp (Fig. 3), force-tuned components explained more population-level variance than components tuned to force-grasp interactions (Fig. 5), and intended forces were accurately predicted from population-level activity across multiple grasps (Figs. 6-8). The study results suggest that in individuals with tetraplegia, to a large extent, force is represented at a high level within motor cortex, distinct from grasp, in accordance with the grasp-independent force encoding model described by Equation 4 (Figs. 4, 5*D*). This conclusion agrees with previous motor control studies (Mason et al., 2004; Chib et al., 2009; Casadio et al., 2015), which suggest that at the macroscopic level, force and motion may be represented independently. In particular, Chib and colleagues showed that descending commands pertaining to force and motion could be independently disrupted via transcranial magnetic stimulation (TMS), and that these commands obeyed simple linear superposition laws when force and motion tasks were combined.

Furthermore, intracortical non-human primate studies (Mason et al., 2006; Hendrix et al., 2009; Intveld et al., 2018) suggest that forces are encoded largely independently of the grasps used to produce them. However, in these studies and within the present work, hand grasps likely recruited overlapping sets of muscle activations. Thus, the relatively low degree of interactions observed here and in the literature could actually be because of overlapping muscle activations rather than truly grasp-independent force representation. For this reason, participant T8 emulated forces using elbow extension in addition to the other hand grasps during session 5. The elbow extension task, which recruited both proximal and distal muscle activations, was chosen to overlap less with the other hand grasps, which recruited distal muscle activations only. In Figure 5, column 3, dPCA was implemented solely on force trials emulated using elbow extension and power grasping. The resulting dPCA composition yielded a slightly larger interaction variance (4%) that was nonetheless smaller than variance due to force (∼12%) or grasp (∼35%). Furthermore, discrete force data, when represented within two-dimensional PCA space, aligned closely with a force axis that was conserved over both power grasping and elbow extension movements, providing further evidence that force may be encoded independently of movements and grasps.

##### Representation of discrete forces

While overall force accuracies exceeded chance performance (Fig. 6), hard and light forces were classified more accurately than medium forces across all hand grasps, sessions, and participants. Medium forces often failed to exceed chance classification performance (Fig. 7*A*; Extended Data Figs. 6-1*B*, 6-3). Notably, classification performance depended on participants’ ability to kinesthetically attempt various force levels and grasps without feedback, despite having tetraplegia for several years before study enrollment. Anecdotally, participant T8 reported that light and hard forces were easier to attempt than medium forces, because they fell at the extremes of the force spectrum and could thus be reproduced consistently. Although his confidence with reproducing all forces improved with training, it is conceivable that without sensory feedback, medium forces were simply more difficult to emulate, and thus yielded neural activity patterns that were less consistent and more difficult to discriminate.

Additionally, prior studies suggest that neural activity increases monotonically with increasing force magnitude (Evarts, 1969; Thach, 1978; Cheney and Fetz, 1980; Wannier et al., 1991; Ashe, 1997; Cramer et al., 2002). Therefore, by virtue of being intermediate to light and hard forces, medium forces may be represented intermediate to light and hard forces in the neural space, and may thus be more easily confused with forces at the extremes of the range evaluated (Murphy et al., 2016; Downey et al., 2018). To this point, population-level activity during medium and light forces exhibited similarities (Fig. 5; Extended Data Fig. 5-1); accordingly, medium forces were most often confused with light forces during offline classification (Fig. 7).

#### Hand posture affects force representation and force classification accuracy

##### Single-feature versus population interactions between force and grasp

As previously stated, force information was neurally represented, and could be decoded, across multiple hand grasps (Figs. 3, 5–8). However, hand grasp also influenced how force information was represented within (Fig. 5) and decoded from (Fig. 7; Extended Data Fig. 7-3) motor cortex. Furthermore, despite small force-grasp interaction population-level variance (Fig. 5*B*; Extended Data Fig. 5-1*B*), as many as 12.0% and 13.8% of neural features exhibited tuning to these interaction effects in participants T8 and T5, respectively (Fig. 3), providing further evidence that the force and grasp representation are not entirely independent.

When considering the relatively large number of interacting features and the small population-level interaction variance, one might initially conclude that a discrepancy exists between feature-level and population-level representation of forces and grasps. However, the amount of variance explained by a parameter of interest may not always correspond directly to the percentage of features tuned to this parameter. Here, the interaction effects within individual features likely reached statistical significance with small effect size. In other words, while real interaction effects were present within the feature data (Fig. 3), the overall effect was small, as exhibited within the population activity (Fig. 5). From this perspective, the seemingly incongruous feature-level and population-level results actually complement one another and inform our understanding of how forces are represented in motor cortex in individuals with tetraplegia.

##### Force and grasp have both abstract (independent) and motoric (interacting) representations in cortex

Thus far, studies of force versus grasp representation have largely fallen into two opposing groups. The first proposes that motor parameters are represented independently (Carmena et al., 2003; Mason et al., 2006; Hendrix et al., 2009; Intveld et al., 2018). Such representation implies that the motor cortex encodes an action separately from its intensity, then combines these two events downstream to compute the EMG patterns necessary to realize actions in physical space.

In contrast, the second group suggests that force, grasp, and other motor parameters interact (Hepp-Reymond et al., 1999; Degenhart et al., 2011). They propose that motor parameters cannot be fully de-coupled (Kalaska, 2009; Branco et al., 2019) and that it may be more effective to use the entire motor output to develop a comprehensive mechanical model, rather than trying to extract single parameters such as force and grasp (Ebner et al., 2009).

The current study presents evidence supporting both independent and interacting representations of force and grasp in individuals with tetraplegia. These seemingly contradictory results actually agree with a previous non-human primate study that recorded from motor areas during six combinations of forces and grasps (Intveld et al., 2018). Intveld and colleagues found that, while force-grasp interactions explained only 0–3% of population variance, roughly 10–20% of recorded neurons exhibited such interactions, which is highly consistent with the present study results (Figs. 3, 5). Thus, in individuals with tetraplegia, the neural space could consist of two contingents: one that encodes force at a high level independent of grasp and motion, and another that encodes force as low-level tuning to muscle activity, resulting in interactions between force and grasp. The second contingent, however small, significantly impacts how accurately forces and grasps are decoded (Fig. 7*B*; Extended Data Fig. 7-3) and should not be discounted.

#### Hand grasp is represented to a greater degree than force at the level of the neural population

##### Go-phase grasp representation

In the present datasets, grasps were decoded more accurately (Figs. 6, 7; Extended Data Fig. 6-1*B*) and explained more signal variance (Fig. 5*B*; Extended Data Fig. 5-1*B*) than forces. This suggests that within the sampled region of motor cortex, grasp is represented to a greater degree than force, which agrees with prior literature (Hendrix et al., 2009; Milekovic et al., 2015; Intveld et al., 2018).

In the current work, force may be represented to a lesser degree than grasp for several reasons. First, force information may have stronger representation in caudal M1, particularly on the banks of the central sulcus (Kalaska and Hyde, 1985; Sergio et al., 2005; Hendrix et al., 2009) or within the depth of the sulcus (Rathelot and Strick, 2009), which cannot be accessed using planar microelectrode arrays. Second, force-tuned neurons in motor cortex respond more to the direction of applied force than its magnitude (Kalaska and Hyde, 1985; Kalaska et al., 1989; Taira et al., 1996). Finally, intracortical non-human primate studies (Georgopoulos et al., 1983, 1992) and human fMRI studies (Branco et al., 2019) suggest that motor cortical neurons respond more to the dynamics of force than to static force tasks. The present work, which recorded from rostral motor cortex while study participants emulated static, non-directional forces, may therefore have detected weaker force representation than would have been possible from more caudally-placed recording arrays during a dynamic, functional force task.

Additionally, both study participants were deafferented and received no sensory feedback regarding the forces and grasps they attempted. In individuals with tetraplegia, discrepancies may exist between the representation of kinematic parameters such as grasp, which remain relatively intact because of their reliance on visual feedback, and kinetic parameters such as force (Rastogi et al., 2020). Specifically, since force representation relies heavily on somatosensory feedback (Tan et al., 2014; Tabot et al., 2015; Schiefer et al., 2018), whose neural pathways are altered during tetraplegia (Solstrand Dahlberg et al., 2018), the current study may have yielded weaker force-related representation than if this feedback were present. Therefore, further investigations of force representation are needed in individuals with tetraplegia during naturalistic, dynamic tasks that incorporate sensory feedback, either from intact sensation or from intracortical microstimulation (Flesher et al., 2016), to determine the full extent of motor cortical force representation and to maximize decoding performance.

##### Grasp representation during prep and stop phases

Unlike forces, which were represented primarily during the go phase of the trial, grasps were represented throughout the entire task (Figs. 5, 6), in agreement with previous literature (Milekovic et al., 2015). However, this ubiquitous grasp representation may be partially explained by the behavioral task. Research sessions consisted of multiple data collection blocks, each of which was assigned to a particular hand grasp, and cycled through three attempted force levels within each block (Fig. 1*B*). Thus, while attempted force varied from trial to trial, attempted hand grasps were constant over each block and known by participants in advance. When individuals have prior knowledge of one task parameter, but not another other, information about the known parameter can appear within the baseline activity (Vargas-Irwin et al., 2018). Therefore, grasp-related information may have been represented within the neural space during non-active phases of the trial, simply by virtue of being known in advance.

Additionally, the placement of the recording arrays could have influenced grasp representation in this study. In each participant, two microelectrode arrays were placed within the hand knob of motor cortex (Yousry et al., 1997). These arrays may have recorded from “visuomotor neurons,” which modulate both to grasp execution and to the presence of graspable objects before active grasp (Carpaneto et al., 2011), or from neurons that are involved with motor planning of grasp (Schaffelhofer et al., 2015). These neurons have typically been attributed to area F5, a homolog of premotor cortex in non-human primates. Recent literature indicates that human precentral gyrus is actually part of the premotor cortex (Willett et al., 2020). Thus, the arrays in this study likely recorded from premotor neurons, which modulate to grasp during both visuomotor planning and grasp execution, as was observed here.

### Implications for force decoding

#### Hand grasp affects force decoding performance

Our decoding results demonstrate that, in individuals with tetraplegia, forces can be decoded offline from neural activity across multiple hand grasps (Figs. 6-8). These results agree with the largely independent force and grasp representation within single features (Fig. 3) and the neural population (Fig. 5). From a functional standpoint, this supports the feasibility of incorporating force control into real-time iBCI applications. On the other hand, grasp affects how accurately discrete forces are predicted from neural data (Fig. 7*B*; Extended Data Fig. 7-3). Therefore, future robust force decoders may need to account for additional motor parameters, including hand grasp, to maximize performance.

#### Decoding motor parameters with dynamic neural representation

The present study decoded intended forces from population activity at multiple time points, with the hope that force representation and decoding performance would be preserved throughout the go phase of the task. We found that feature-level (Fig. 2; Extended Data Fig. 2-1) and population-level (Fig. 5; Extended Data Fig. 5-1) force activity exhibited both tonic and dynamic characteristics in individuals with tetraplegia.

When participants attempted to produce static forces, the resulting neural activity varied with time to some degree. These dynamics are consistent with previous results in humans (Murphy et al., 2016; Downey et al., 2018; Rastogi et al., 2020). In particular, Downey and colleagues found that force decoding during a virtual, open-loop, grasp-and-transport task was above chance during the grasp phase of the task, but no greater than chance during static attempted force production during the transport phase. These results support the idea that deafferented motor cortex encodes changes in force, rather than (or in addition to) discrete force levels themselves, as in the able-bodied case (Smith et al., 1975; Georgopoulos et al., 1983, 1992; Wannier et al., 1991; Picard and Smith, 1992; Boudreau and Smith, 2001; Paek et al., 2015).

However, the presence of tonic elements agrees with intracortical studies (Smith et al., 1975; Wannier et al., 1991), which demonstrated both tonic and dynamic neural responses to executed forces; and fMRI studies (Branco et al., 2019), which demonstrated a monotonic relationship between the BOLD response and static force magnitudes. Moreover, despite the presence of dynamic response elements, offline force classification performance remained relatively stable throughout the go phase (Fig. 6; Extended Data Figs. 6-1, 6-3), suggesting that the tonic elements could allow for adequate real-time force decoding using linear techniques alone. This may be especially true when decoding forces during dynamic functional tasks, which elicit stronger, more consistent neural responses within motor cortex (Georgopoulos et al., 1983, 1992; Branco et al., 2019).

Nonetheless, real-time force decoding would likely benefit from an exploration of a wider range of encoding models. For example, the exploration of a force derivative model, and its implementation within an online iBCI decoder, would be of potential utility.

#### Decoding of discrete versus continuous forces

The present work continues previous efforts to characterize discrete force representation in individuals with paralysis (Cramer et al., 2005; Downey et al., 2018; Rastogi et al., 2020) by accurately classifying these forces across multiple hand grasps – especially when performing light versus hard force classification (Fig. 7). This supports the feasibility of enabling discrete (“state”) control of force magnitudes across multiple grasps within iBCI systems, which would allow the end iBCI user to perform functional grasping tasks requiring varied yet precise force outputs. Perhaps because discrete force control alone would enhance iBCI functionality, relatively few studies have attempted to predict forces along a continuous range of magnitudes. Thus far, continuous force control has been achieved in non-human primates (Carmena et al., 2003) and able-bodied humans (Pistohl et al., 2012; Chen et al., 2014; Flint et al., 2014), but not in individuals with tetraplegia. If successfully implemented, continuous force control could restore more nuanced grasping and object interaction capabilities to individuals with motor disabilities.

However, during the present work (Fig. 7; Extended Data Fig. 6-1) and additional discrete force studies (Murphy et al., 2016; Downey et al., 2018), intermediate force levels were often confused with their neighbors, and thus more difficult to decode. Therefore, implementing continuous force control may pose challenges in individuals with tetraplegia. Possibly, enhancing force-related representation in these individuals via aforementioned techniques, including the introduction of dynamic force tasks, closed loop sensory feedback, and derivative force encoding models, may boost overall performance to a sufficient degree to enable continuous force decoding capabilities. Regardless, more investigations are needed to determine the extent to which continuous force control is possible in iBCI systems for individuals with tetraplegia.

In conclusion, this study found that, while force information was neurally represented and could be decoded across multiple hand grasps in a consistent way, grasp type had a significant impact on force classification accuracy. From a neuroscientific standpoint, these results suggest that force has both grasp-independent and grasp-dependent (interacting) representations within motor cortex in individuals with tetraplegia. From a functional standpoint, they imply that to incorporate force as a control signal in human iBCIs, closed-loop force decoders should ideally account for interactions between force and other motor parameters to maximize performance.

## Synthesis

Reviewing Editor: Michael Michaelides, NIDA-NIH Decisions are customarily a result of the Reviewing Editor and the peer reviewers coming together and discussing their recommendations until a consensus is reached. When revisions are invited, a fact-based synthesis statement explaining their decision and outlining what is needed to prepare a revision will be listed below. The following reviewer(s) agreed to reveal their identity: Thomas Brochier.

Reviewer 1

Review of manuscript eN-NWR-0231-20: “The neural representation of force across grasp types in motor cortex of humans with tetraplegia"

This paper explores how force information is encoded in human motor cortex and how it can be decoded and incorporated into real-time iBCI applications. To do so, the authors analyzed high-density multi-electrode recordings from Utah-arrays chronically implanted in the motor cortex of 2 tetraplegic patients during performance of a squeeze imagery task. The methods are clearly described and the analyses are sounds. On overall, they are closely related to the methods used in the recently published study of Rastogi et al., (2020) looking at very similar datasets. With respect to this companion paper, the current study focuses more closely on the interactions between the neural coding of kinematic (grasp) and kinetic (force) information across a broader set of grasp types. However, the two papers share the same objective of investigating “the feasibility of incorporating discrete force control into human operated iBCIs”.

The paper’s conclusion would have been stronger with a better experimental design. As rightly pointed out by the authors, “restoring the ability to grasp and interact with objects requires both kinematic and kinetic information”. In relation to kinetic information, the seminal work of Westling and Johansson (1984) showed that force control involves a tight coordination between grip and load forces that vary in relation to 2 main object properties: the object weight and the object friction. The way the experimental task presented here relates to force control during object manipulation is rather confusing. Indeed, the subjects don’t have to emulate grasp forces related to the object physical properties but have to emulate squeeze forces based on visual and auditory cue.

The distinction between grip force and squeeze force is critical and not really straightforward in the examples given in figure 1. For instance the stress ball is likely to be heavier than the tennis ball and would require higher grip/load forces to be held in the hand. All in all, I’m not convinced that the object pictures are helping at all the subject to produce the right level of force. I would have rather opted for the picture of a similar object such as the sponge with 3 different level of squeezing (that would be clearly visible on the pictures) or 3 objects of similar shapes but with increasing weights, e.g. a tennis ball, a stress ball and a metal ball that would emulate 3 force levels to hold and lift the object. The lack of coherence between force and object representation may enforce interaction effects between force and grasp representations. More generally, it is not clear how the representation of squeeze force upon instruction may translate into grip force control for object manipulation. In everyday life, force control is driven by the object physical properties and not by verbal instructions. If these instructions are essential, it would be more straightforward to directly program the artificial actuator (i.e. a robotic arm) to produce discrete force levels.

My second main concern relates to the high variability of the observed effects across sessions. For instance, force effects are dominating in session 1, grasp effects are dominating in sessions 2 to 4 and session 5 combines grasp and force effects (Figure 3). As a result, the proportion of features tuned to force varies considerably across sessions (from 15.4 to 54.7%). The neural population analysis has been applied to sessions 5 and 7 containing the highest percentage of features tuned to force (red and blue columns in figure 3). It would have been informative to also run the neural population analysis in one of the session 2 to 4 in which grasp representation is dominant (similar to figure 6-2 but for force-classification accuracies). Would time-dependent classification accuracies for force be still significant in these sessions? Figure 6-1 and 7-1 already suggests that the time course of time-dependant classification accuracies for force would look rather poor in these sessions. In terms of interpretations, it questions how variable force representation can be exploited for force coding into real-time iBCI applications?

Minor

Figure 1A is the exact copy of figure 1A of Rastogi et al (2020) published in scientific report. An authorization for reproduction may be required.

In figure 1, there is also mismatch in the figure legend for the 8 and 15-lb dumbbell which are inverted with respect to the pictures.

During kinesthetic imagery, is the pinch performed by the experimenter always a closed pinch or is it adjusted to the other pinches during open pinch or ring pinch blocks, respectively?

Intro: Some relevant studies in the domain are not mentioned. The exploration of kinetic and kinematic information as an iBCIs control signal for grasping has been directly addressed in some earlier studies using intracotrical recordings with Utah arrays (e.g. Milekovic et al., Neuroimage 114:338-55, 2015 in macaque monkeys). Although Milekovic et al are using a slightly different task, they report time-dependent decoding performances using high and low frequency LFP modulations very similar to the performances described in the current paper. During the GO phase, the decoding accuracy is higher (>0.8) for grasp than for force (around 0.6). Additionally, the decoding accuracy is high during all trial phases for grasp but only during the GO phase for force.

Methods: The authors should explain better why they selected specifically these four different hand postures for hand grasp. The open pinch and closed pinch are two precision grip between the thumb and index that only differs by the posture of the unused fingers (D3-D5). The ring pinch doesn’t seem very natural. It is actually rather surprising that some features show such a larger difference between cp and op.

Could the authors specify the type of amplifier used during the experiment? Is it a Cereplex-E from Blackrock Microsystems?

In figure 1A, it is indicated that SBP is extracted between 500 and 5000 Hz, the text indicates 250 to 5000 Hz. The link between the SBP illustration in figure 1A and the SPB features is not straightforward. In figure 1A, SPB modulations are frequency resolved in small frequency bands between 500 and 5000 Hz. However, as far as I understand from the methods, the SPB features are computed from a single RMS measure within a broad frequency band (500-5000 Hz).

Additionally, I didn’t really get that the 384 neural features corresponded to the 192 TC + 192 SBP features before reading the result section. It would help to move from the result section to the method section the sentence: “TC features are labelled from 1-192 according to the recording electrodes from which they were extracted. Corresponding SBP features are labelled from 193-384.”

Comparing force representation between power grasping and arm movements involved different force ranges

Discussion:

"the hand grasps used to produce forces likely recruited overlapping sets of muscle activations. For this reason, participant P1 emulated forces using elbow extension in addition to the other hand grasps during Session 5.”

Actually, the “Elbow” task is far from purely proximal since as illustrated in figure 1B, the subject has to use a power grip to hold and lift the dumbbell which likely requires overlapping sets of muscle activations with the other grasps, and in particular with the power grasp. EMG recordings in control subjects would have been necessary to disentangle this issue.

Related to this, it is also possible that interaction effects would have been stronger if recordings were done in the depth of the central sulcus, as suggested later in the discussion. This could be due to the fact that the most direct projections to the spinal cord, i.e. the cortico-motoneuronal cells, are located in the bank of the central sulcus in macaque monkeys (Rathelot and Strick, 2009). The authors could maybe emphasize that recordings in the depth of the central sulcus are not possible with Utah arrays.

Reviewer 2

BCI is an exciting approach that aims to provide and restore motor control in individuals suffering from an irreversible loss of descending control over muscles. Several studies have explored implementing iBCI in individuals with tetraplegia. This paper describes an effort to resolve the coding of grasp type and force levels in such individuals after chronic arrays (n=2) were implanted in the “hand knob” area of the motor cortex. Subjects were instructed to make different types of grasps at three different force levels. The authors used an extracted neural features to test the relationships between cortical activity and task parameters. They found that grasp type and force levels were both coded by motor cortical activity but not at equal magnitude. A small set of features expressed interactions in coding between force levels and grasp types. Projecting the data into a lower dimensional space (using dPCA or PCA) revealed that an additive model which assumes independent coding of grasp type and force level captured the data more adequately than a scalar model. In the second part of the study the authors attempted to decode the force level and grasp type from the obtained neural features. Here they showed that grasp type was more accurately decoded than force levels. However, decoding performances improved when considering only extreme force levels (while omitting intermediate levels). Finally, in some cases, decoding force level improved when considering specific grasp types, consistent with some level of interaction between the tuning to these two parameters. The authors conclude that force levels and grasp types are both represented in motor cortical cells but to a different extent. Understanding the interactions between these parameters would be useful for improving decoding accuracy. The authors further conclude that continuous force control may not be obtained using decoded signals and could possible require some other means such as closed-loop control, etc.

The paper presents an interesting perspective on an important question and uses a remarkable dataset. Nevertheless, several points need to be addressed.

1. The authors frequently use the results to make statements about the motor cortical coding of force in general. I am not sure this approach is valid. Normally, motor cortical cells receive sensory feedback even before movement starts. This input may change the cortical coding of force and grasp. In addition, the motor cortical circuitry of individuals with long term tetraplegia may undergo substantial changes which could further complicate the comparison to the intact motor cortical circuitry. The validity of this deduction (from deprived to intact motor cortex) needs to be addressed. Moreover, since in my opinion the main value of the study is in its decoding effort and its future relevance to iBCI.

2. Motor cortical activity was shown to respond to visual cues when they are relevant to the movement. Since the grasp type and force magnitude were signaled by different cues, could the response reflect in part a cue-driven and not motor-related activity? More generally, could these neurons be part of a mirror system?

3. I am not sure of the added value of the demixing analysis in this paper, beyond the ANOVA and PCA analyses. A clearer explanation of this value would be helpful.

4. I am not convinced that the results support the additive over the scalar model. It seems to me that the data do not fit either model. Is there a way to quantify the similarity to one model vs. the other? Additionally, I think that it would help to plot the pie charts (Figure 6B) without the CI fraction to compare these percentages better with those shown in Figure 5.

5. The decoding was done on one session but the session-to-session variability in tuning fraction was very large (as evident from Figure 3). To what extent did the quality of decoding vary across sessions? Given this large variability, can we rely on these signals for long-term decoding as needed for BCI? To what extent is this variability of coding force and grasp types compared to variations in other tested parameters (such as direction of movement) obtained in other studies?

Minor points

1. The Method section describing the extraction of neural features was not very clear to me. I think a better explanation of the steps used to extract these features and their similarity to more commonly used neural signals would be helpful.

2. I don’t quite follow the rationale for using session 5 in P1 which contains another condition which was not tested in P2. Why not use session 6 from P1 which has a similar structure as the one used in P2? Wouldn’t this added condition interact with the results obtained for the grasp/force conditions?

3. The CI component behaves differently in P1 vs. P2. In particular, for P2 the CI explains 80% of the variance (as opposed to 50% in P1). Is there an intuitive explanation for this difference? For example, a different location of arrays, number of task-related channels in the arrays, etc.

4. In my mind, one of the most interesting results is the earlier coding of grasp type vs. force levels (Fig. 6). I was therefore a bit disappointed to learn that this was probably an outcome of the task design. Was there any attempt to reverse the arrangement of trials in blocks, where successive trials all had the same force level but a different grasp type?

## Author Response

Dear Dr. Michaelides,

Thank you for giving us the opportunity to submit a revised version of our manuscript entitled, “The neural representation of force across grasp types in motor cortex of humans with tetraplegia” to eNeuro. We appreciate the time and effort that you and the reviewers spent to providing your valuable feedback on our manuscript. We have incorporated changes to reflect most of the suggestions provided by the reviewers. Here is a point-by-point response to the reviewers’ comments and concerns.

Synthesis of Reviews

Comment: Computational Neuroscience Model Code Accessibility Comments for Author (Required): The authors are asked to include relevant information regarding code accessibility, indicating whether and how the code can be accessed, including any accession numbers or restrictions, as well as the type of computer and operating system used to run the code. This should be added to the methods section which should also be updated to include all relevant information (e.g. first paragraph of methods section).

Response: A new “Data and Code Accessibility” section has been added to the end of the Methods section to address these issues. Additionally, new language regarding the computer and OS used to run the code has been added to the Methods. All of these changes are highlighted in yellow.

Comments from Reviewer 1

Comment: This paper explores how force information is encoded in human motor cortex and how it can be decoded and incorporated into real-time iBCI applications. To do so, the authors analyzed high-density multi-electrode recordings from Utah-arrays chronically implanted in the motor cortex of 2 tetraplegic patients during performance of a squeeze imagery task. The methods are clearly described and the analyses are sounds. On overall, they are closely related to the methods used in the recently published study of Rastogi et al., (2020) looking at very similar datasets. With respect to this companion paper, the current study focuses more closely on the interactions between the neural coding of kinematic (grasp) and kinetic (force) information across a broader set of grasp types. However, the two papers share the same objective of investigating “the feasibility of incorporating discrete force control into human operated iBCIs”.

Response: Thank you for this accurate and favorable summary of our manuscript.

Comment: The paper’s conclusion would have been stronger with a better experimental design. As rightly pointed out by the authors, “restoring the ability to grasp and interact with objects requires both kinematic and kinetic information”. In relation to kinetic information, the seminal work of Westling and Johansson (1984) showed that force control involves a tight coordination between grip and load forces that vary in relation to 2 main object properties: the object weight and the object friction. The way the experimental task presented here relates to force control during object manipulation is rather confusing. Indeed, the subjects don’t have to emulate grasp forces related to the object physical properties but have to emulate squeeze forces based on visual and auditory cue. The distinction between grip force and squeeze force is critical and not really straightforward in the examples given in figure 1. For instance the stress ball is likely to be heavier than the tennis ball and would require higher grip/load forces to be held in the hand. All in all, I’m not convinced that the object pictures are helping at all the subject to produce the right level of force. I would have rather opted for the picture of a similar object such as the sponge with 3 different level of squeezing (that would be clearly visible on the pictures) or 3 objects of similar shapes but with increasing weights, e.g. a tennis ball, a stress ball and a metal ball that would emulate 3 force levels to hold and lift the object. The lack of coherence between force and object representation may enforce interaction effects between force and grasp representations. More generally, it is not clear how the representation of squeeze force upon instruction may translate into grip force control for object manipulation. In everyday life, force control is driven by the object physical properties and not by verbal instructions. If these instructions are essential, it would be more straightforward to directly program the artificial actuator (i.e. a robotic arm) to produce discrete force levels.

Response: The goal of this work was to characterize the representation of force in general, in order to further elucidate the feasibility of extracting kinetic control signals for use in a closed-loop iBCI. Our understanding of your comment is that there is a distinction between “grip force,” which is the amount of force needed to grasp an object with a particular weight and friction as per Westling and Johansson, 1984 - and “squeeze force,” which is an additional amount of force used to crush the object. In this study, we felt that squeeze forces would be more intuitive for our participants to emulate than grip forces. In able-bodied individuals, grip forces are informed by intact tactile and proprioceptive feedback and often produced without much cognitive effort. Since our participants were deafferented and lacked tactile and proprioceptive feedback, we believed they would have difficulty emulating grip forces in response to object properties such as weight and friction, even with visual feedback such as that suggested in this comment (i.e., a tennis ball versus a stress ball versus a metal ball). Squeeze forces, in contrast, can conceivably be generated without proprioceptive or tactile feedback and are easier to represent visually, so we thought that the participants would be able to emulate squeeze forces more reliably than grip forces. Additionally, fMRI studies have shown that many neural mechanisms underlying squeeze/crush force production are similar between able-bodied persons performing and imagining, vs persons with chronic spinal cord injury simply imagining these movements (Cramer, 2005). We believe that our study of squeeze forces still informed our understanding of how forces in general are represented in motor cortex, and that our main study results hold regardless of the type of force emulated. However, we do agree that the manuscript would be clearer if we made the distinction between grip and squeeze forces. Therefore, within the updated Methods section, we have added language that distinguishes squeeze forces from grip forces. Additionally, we have provided a brief explanation of why participants were instructed to attempt squeeze forces as opposed to grip forces. These additions are highlighted in yellow.

Here, we provided participants with visual cues to help them contextualize the concept of light, medium, and hard squeezing forces, i.e., to make these forces seem less abstract after years of deafferentation. We have added a sentence (highlighted in yellow) to the updated Methods section to underscore this point. We elected not to present a single object with three different levels of squeezing, as suggested in this comment, because we believed this would introduce a confound between hand aperture (a kinematic parameter) versus force level (a kinetic parameter). Instead, we presented different objects for particular forces and grasps and instructed our participants to think about the amount of force needed to crush the object squeezed by the researcher. We felt that this approach would help participants de-couple the concept of squeeze force from hand aperture.

We recognize that the current study design presents limitations. To begin with, participants may have emulated both grip and squeeze forces during the instructed task. However, we do not feel that this dual emulation would have significantly impacted the main results of the study for two reasons. First, we made an effort to choose objects of similar weights and sizes to avoid grip-force-related confounds, and have updated the Methods to reflect this. (For example, the stress ball and tennis ball used in this study were of comparable weights.) Second, participants were specifically instructed to emulate the amount of force needed to crush the various objects, and were not asked to think about supporting the object against gravity. Third, these verbal instructions were reinforced by the visual cues; participants were provided with visual feedback of how much force was needed to deform the various object, which corresponded to squeeze forces, and did not receive any feedback about the objects’ weight or friction. (The exception was during Session 5, when participants were asked to think about lifting dumbbells of various weights.) For these reasons, we believe that the instructed squeeze forces are represented to an overwhelmingly greater degree than grip force in the current dataset, and that there was coherence between the intended force levels and the chosen objects.

A second limitation, which you point out, is that instructed squeeze forces are not typically used to achieve force control in everyday life; instead, able-bodied individuals volitionally modulate output grip force in response to object properties. Ideally, to acquire the most complete picture of how neural activity is modulated by object-driven force generation, we would record intracortical activity, EMG activity, and contact force levels from an able-bodied individual with intact tactile and proprioceptive feedback, while they grasped objects of varying weights and friction levels. This study is, of course, impossible to conduct in humans for ethical reasons. In the present work, participants could not generate forces, which precluded us from recording output forces and EMG activity. Furthermore, due to their lack of somatosensory feedback, we felt that instructed squeeze forces would be easier to emulate than object-informed grip forces, as described earlier. In other words, the experiment was designed to ensure that our deafferented participants were emulating forces in a definitive, predictable manner, so that we could be sure that the observed neural activity was in response to force. We designed the experiment with the idea that our setup could be translated to a closed-loop setting - i.e., if a participant saw a sponge, a stress ball, or a tennis ball in real time and wanted to squeeze these objects, they could use a force decoder trained on the open-loop data to volitionally output the appropriate amount of force without receiving verbal instructions. This setup, while somewhat contrived, could be a preliminary step in helping participants self-initiate various force levels in response to objects. The control of actual grip forces (as opposed to squeeze forces) could conceivably be implemented using a similar closed-loop control scheme, albeit over a smaller range of forces. However, such a control scheme would require the restoration of somatosensory feedback about object properties in individuals with tetraplegia, which is the subject of future investigations.

Comment: My second main concern relates to the high variability of the observed effects across sessions. For instance, force effects are dominating in session 1, grasp effects are dominating in sessions 2 to 4 and session 5 combines grasp and force effects (Figure 3). As a result, the proportion of features tuned to force varies considerably across sessions (from 15.4 to 54.7%). The neural population analysis has been applied to sessions 5 and 7 containing the highest percentage of features tuned to force (red and blue columns in figure 3). It would have been informative to also run the neural population analysis in one of the session 2 to 4 in which grasp representation is dominant (similar to figure 6-2 but for force-classification accuracies). Would time-dependent classification accuracies for force be still significant in these sessions? Figure 6-1 and 7-1 already suggests that the time course of time-dependant classification accuracies for force would look rather poor in these sessions. In terms of interpretations, it questions how variable force representation can be exploited for force coding into real-time iBCI applications?

Response: Agreed, at the single feature level, there is indeed high variability in the number of force-tuned features across sessions. This is in contrast to what we observe at the population level (Figure 7-1), where force actually accounts for similar amounts of variance across multiple sessions, including Sessions 2 (2%), 4 (3%), 5 (6%), and 7 (4%), as presented in Figure 5-1.

To address your questions about how these feature- and population-level representations of force would translate to decoding performance, we have created an additional Figure 6-3, in which we present time-varying force classification accuracies for individual sessions. Light, medium, and hard force classification accuracies are relatively consistent across sessions, suggesting that population-level force representation (as opposed to single-feature representation) is what truly matters from a decoding perspective. More importantly, since the population-level representation appears relatively stable across multiple datasets, this would suggest that force coding for real-time iBCI applications would have consistent performance across multiple days.

As an aside, under the “Single-Feature Versus Population Interactions between Force and Grasp” heading of the Discussion (highlighted in blue), we outline the apparent discrepancy between the neural representation at the single-feature level (Figure 3) versus at the population level (Figure 5) and, in particular, state that “...the amount of [population-level] variance explained by a parameter of interest does not always correspond directly the percentage of features tuned to this parameter.” In other words, the number of features tuned to force may vary greatly across sessions due to a number of factors - for example, electrode micromotion, variable noise thresholds, and even the statistical thresholds used to determine feature tuning properties - but across the population as a whole, the degree to which forces are represented remains consistent across multiple sessions.

Minor Comments (General):

a. Figure 1A is the exact copy of figure 1A of Rastogi et al (2020) published in scientific report. An authorization for reproduction may be required.

b. In figure 1, there is also mismatch in the figure legend for the 8 and 15-lb dumbbell which are inverted with respect to the pictures.

c. During kinesthetic imagery, is the pinch performed by the experimenter always a closed pinch or is it adjusted to the other pinches during open pinch or ring pinch blocks, respectively?

Response:

a. The article Rastogi et al (2020) is licensed under a Creative Commons Attribution 4.0 International license, which allows adaptation and reproduction of the article’s contents as long as appropriate credit is attributed to the journal and authors, a link is provided to the Creative Commons license, and any changes are indicated. Accordingly, the legend of Figure 1 has been updated as follows: “Figure 1A is reprinted by permission from Springer Nature (https://www.nature.com/srep/) as indicated in the Terms and Conditions of the Creative Commons Attribution 4.0 International license (http://creativecommons.org/licenses/by/4.0/).”

b. Figure 1B has now been corrected so that the 8- and 15-lb dumbbell are correctly attributed to medium and hard forces, respectively.

c. The experimenter adjusted the pinch to the other pinches during open pinch and ring pinch blocks. The legend of Figure 1B has been accordingly updated as follows: “During pinch blocks, the researcher squeezed the pinchable objects (cotton ball, eraser, nasal aspirator tip) using the particular pinch grip dictated by the block (r-pinch, o-pinch, c-pinch). Here, only closed pinches of objects are shown.” Additionally, the Participant Task section of the Materials and Methods section has been updated to clarify this point. All changes are highlighted in yellow.

Minor Comment (Introduction): Some relevant studies in the domain are not mentioned. The exploration of kinetic and kinematic information as an iBCIs control signal for grasping has been directly addressed in some earlier studies using intracotrical recordings with Utah arrays (e.g. Milekovic et al., Neuroimage 114:338-55, 2015 in macaque monkeys). Although Milekovic et al are using a slightly different task, they report time-dependent decoding performances using high and low frequency LFP modulations very similar to the performances described in the current paper. During the GO phase, the decoding accuracy is higher (>0.8) for grasp than for force (around 0.6). Additionally, the decoding accuracy is high during all trial phases for grasp but only during the GO phase for force.

Response: Thank you for pointing out this important study. We have now cited the Milekovic paper in the updated Introduction and Discussion sections. All additions are highlighted in yellow.

Minor Comments (Materials and Methods):

a. The authors should explain better why they selected specifically these four different hand postures for hand grasp. The open pinch and closed pinch are two precision grip between the thumb and index that only differs by the posture of the unused fingers (D3-D5). The ring pinch doesn’t seem very natural. It is actually rather surprising that some features show such a larger difference between cp and op.

b. Could the authors specify the type of amplifier used during the experiment? Is it a Cereplex-E from Blackrock Microsystems?

c. In figure 1A, it is indicated that SBP is extracted between 500 and 5000 Hz, the text indicates 250 to 5000 Hz. The link between the SBP illustration in figure 1A and the SPB features is not straightforward. In figure 1A, SPB modulations are frequency resolved in small frequency bands between 500 and 5000 Hz. However, as far as I understand from the methods, the SPB features are computed from a single RMS measure within a broad frequency band (500-5000 Hz).

d. Additionally, I didn’t really get that the 384 neural features corresponded to the 192 TC + 192 SBP features before reading the result section. It would help to move from the result section to the method section the sentence: “TC features are labelled from 1-192 according to the recording electrodes from which they were extracted. Corresponding SBP features are labelled from 193-384.”

e. Comparing force representation between power grasping and arm movements involved different force ranges

Response:

a. The explanation we provide in our updated Methods section (highlighted in yellow) is that we wished to study force representation in the context of individuated finger postures. To put our choice of hand grasps into additional context, we originally sought to study how force representation was affected by only two grasp types - power versus closed pinch - as presented in Session 1 of the current dataset (similar to Rastogi et al, 2020). Participant T8 then expressed that the open pinch grasp was more intuitive for him to emulate than the closed pinch grasp, which led to pilot sessions in which we qualitatively assessed the differences in neural activity during closed versus open pinch. As presented in the main text, we found differences in representation between the open and closed pinch grasps. Due to this intriguing result, we became interested how individuated finger postures were neurally represented, and how this representation interacted with force representation. We specifically wished to incorporate a grasp that involved different digits than those encompassed within the open and closed pinches, which is why we added the third (ring pinch) precision grasp to the task. While the ring pinch may not be as functional in practice, it allowed us to span a wider range of active digits than are often included in studies that use functional precision grasps (usually a combination of the thumb, forefinger, and/or middle finger)

b. No we did not use a Cereplex-E. The Cereplex-E is a digital headstage that digitizes the raw analog signal at the recording site. Rather, our signals, once recorded using the microelectrode array, was transmitted through the BlackRock Patient Cables that provide a noise-immune link between the array and the front-end amplifiers for amplification and digitization. These digitized signals are then sent to the Neuroport system. We provide these details, highlighted in yellow, within the updated Methods section.

c. Thank you for pointing out these issues. Figure 1A should have indicated that SBP features were extracted between 250 and 5000 Hz, rather than between 500 and 5000 Hz. We have updated the figure to correct this typo. Additionally, the SBP modulations indicated in Figure 1A were shown merely for illustration purposes; the frequency “bands” indicated in this panel were actually y-axis tick mark values and were not meant to indicate that our data were resolved into small frequency bands. We have updated this panel so that the tick marks stand out more clearly. Additionally, we have added a bracket to the side of the panel to indicate that the entire broad frequency band (250-5000 Hz) is used to compute SBP features, as described in the Methods. We hope that these modifications help clarify how we extract SBP features.

d. This sentence has been reproduced within the “Feature Extraction” section of the updated Methods for clarification (highlighted in yellow).

e. This is a fair point of consideration. In this study, we had participants emulate the extremes of the force range for each end effector (i.e., the hand or the arm) in order to maximize differences between light, medium, and hard forces. We did this because forces at the extremes of the range were easiest for the participants to emulate, which we felt would yield more consistent patterns within the neural data. However, as you point out, such a design meant that the force ranges emulated by the arm versus the hand were different, which could have artificially introduced interaction effects within Session 5. Indeed, interaction variance is largest in Session 5, possibly due to the difference in force ranges between elbow extension and grasping trials, which we point out in the updated Results section where we present Figure 5B (highlighted in yellow).

Minor Comments (Discussion):

a. "The hand grasps used to produce forces likely recruited overlapping sets of muscle activations. For this reason, participant P1 emulated forces using elbow extension in addition to the other hand grasps during Session 5.”

Actually, the “Elbow” task is far from purely proximal since as illustrated in figure 1B, the subject has to use a power grip to hold and lift the dumbbell which likely requires overlapping sets of muscle activations with the other grasps, and in particular with the power grasp. EMG recordings in control subjects would have been necessary to disentangle this issue.

b. Related to this, it is also possible that interaction effects would have been stronger if recordings were done in the depth of the central sulcus, as suggested later in the discussion. This could be due to the fact that the most direct projections to the spinal cord, i.e. the cortico-motoneuronal cells, are located in the bank of the central sulcus in macaque monkeys (Rathelot and Strick, 2009). The authors could maybe emphasize that recordings in the depth of the central sulcus are not possible with Utah arrays.

Response:

a. Based on your comment, we agree that some overlap likely existed between the elbow extension and hand grasp trials, since the experimenter modeled a power grasp to lift the various weights during elbow extension. We do think that the degree of overlap between the elbow task and the other hand grasps was likely less than the overlap between the power and pincer grasps, due to the proximal aspects of elbow extension. We have updated the indicated sentence in the Discussion to present the differences between elbow extension and the other grasps more conservatively.

b. We agree that a statement regarding the limitations of Utah arrays is warranted. Thus, we have modified our sentence regarding array placement in the Discussion to read as follows: “First, force information may have stronger representation in caudal M1, particularly on the banks of the central sulcus (Kalaska and Hyde, 1985; Sergio et al., 2005; Hendrix et al., 2009) or within the depth of the sulcus (Rathelot and Strick, 2009), which cannot be accessed using Utah recording arrays.” This modification, underlined for emphasis here, is highlighted in yellow within the Discussion.

Comments from Reviewer 2

Comment: BCI is an exciting approach that aims to provide and restore motor control in individuals suffering from an irreversible loss of descending control over muscles. Several studies have explored implementing iBCI in individuals with tetraplegia. This paper describes an effort to resolve the coding of grasp type and force levels in such individuals after chronic arrays (n=2) were implanted in the “hand knob” area of the motor cortex. Subjects were instructed to make different types of grasps at three different force levels. The authors used an extracted neural features to test the relationships between cortical activity and task parameters. They found that grasp type and force levels were both coded by motor cortical activity but not at equal magnitude. A small set of features expressed interactions in coding between force levels and grasp types. Projecting the data into a lower dimensional space (using dPCA or PCA) revealed that an additive model which assumes independent coding of grasp type and force level captured the data more adequately than a scalar model. In the second part of the study the authors attempted to decode the force level and grasp type from the obtained neural features. Here they showed that grasp type was more accurately decoded than force levels. However, decoding performances improved when considering only extreme force levels (while omitting intermediate levels). Finally, in some cases, decoding force level improved when considering specific grasp types, consistent with some level of interaction between the tuning to these two parameters. The authors conclude that force levels and grasp types are both represented in motor cortical cells but to a different extent. Understanding the interactions between these parameters would be useful for improving decoding accuracy. The authors further conclude that continuous force control may not be obtained using decoded signals and could possible require some other means such as closed-loop control, etc. The paper presents an interesting perspective on an important question and uses a remarkable dataset. Nevertheless, several points need to be addressed.

Response: Thank you for this accurate and detailed summary of our manuscript. Please see below for a point-by-point response to your comments.

Comment: The authors frequently use the results to make statements about the motor cortical coding of force in general. I am not sure this approach is valid. Normally, motor cortical cells receive sensory feedback even before movement starts. This input may change the cortical coding of force and grasp. In addition, the motor cortical circuitry of individuals with long term tetraplegia may undergo substantial changes which could further complicate the comparison to the intact motor cortical circuitry. The validity of this deduction (from deprived to intact motor cortex) needs to be addressed. Moreover, since in my opinion the main value of the study is in its decoding effort and its future relevance to iBCI.

Response: We agree that it is very important to distinguish between our findings, which were obtained in individuals with tetraplegia, from the motor coding of force and grasp within the intact, able-bodied motor cortex. Previous literature suggests that, despite a lack of somatosensory feedback and the presence of cortical reorganization in individuals with tetraplegia, these individuals exhibit many similarities in motor-related neural representation to able-bodied individuals (Cramer et al, 2005). Therefore, we believe that to a certain extent, the present study does inform our understanding of how motor parameters such as force and grasp may be represented within general motor cortex. However, we also agree that making generalizations about force control in the intact, able-bodied motor cortex based on our results is not valid, as you rightly point out. For this reason, under the “Go-Phase Grasp Representation” heading of the Discussion, we touched on how a lack of somatosensory feedback affects the neural representation of force in our participants with tetraplegia (highlighted in blue). Additionally, we have added the phrase “in individuals with tetraplegia” and other relevant descriptors (highlighted in yellow) throughout the Discussion, in order to more directly distinguish our results and the motor cortical coding of force in general.

Comment: Motor cortical activity was shown to respond to visual cues when they are relevant to the movement. Since the grasp type and force magnitude were signaled by different cues, could the response reflect in part a cue-driven and not motor-related activity? More generally, could these neurons be part of a mirror system?

Response: Yes, as we indicate in the Discussion, we likely recorded from visuomotor neurons, also known as “mirror neurons,” located within the premotor cortex. These neurons may have accounted for the above-chance representation of grasp outside of the active “go” phase of the task. In other words, neural activity within the preparatory and stop phases may have at least partially contained a cue-driven response. However, we believe that any cue-driven effects within the go phase were relatively minor, as we indicate briefly within the updated Methods. A more detailed explanation is included below.

In previous work, we evaluated the effects of visual cues on the neural response to observed, imagined, and attempted forces in individuals with tetraplegia. Specifically, we compared neural data generated while visual cues were included versus omitted. We found that visual cues introduced activity peaks during the preparatory and stop phases of the task, but had relatively little effect on the go-phase neural activity. Furthermore, effects of visual cues tended to diminish when participants attempted (versus observed or imagined) force production. Finally, neural activity generated without visual cues exhibited similar force-related trends as neural activity generated with visual cues. In light of these previous study results, it is likely that the presence of visual cues had a relatively minor influence on the neural response to attempted grasping forces within the current manuscript.

We did not explicitly evaluate the effects of visual cues on grasp representation, e.g., by collecting datasets in which participants attempted different grasp types with versus without visual cues. However, an additional study by Vargas-Irwin et al, 2018 suggests that a core network of neurons may be recruited during both observed and attempted kinematic tasks, and that neurons within this core network exhibit increased firing rates during attempted versus observed actions. Even though kinematic parameters were represented to similar extents during both action observation and attempt, the neural activity patterns were nonetheless different during these cognitive states, which suggests that the neural activity during attempted action encompassed more than just a cue-driven response to kinematic parameters. Furthermore, in a non-human primate study by Milekovic et al, 2015, neural activity generated during executed forces and grasps exhibited similar trends as those produced in the current work; go-phase grasp representation exceeded go-phase force representation, and grasps were represented during the preparatory phase of the task. Crucially, the non-human primates in the Milekovic study did not watch a researcher mime these parameters during experimental trials. Instead, forces and grasps were indicated with various patterns of LED lights, which would have been more likely to elicit cue-driven effects in visual cortex rather than in motor cortex. In light of the Vargas-Irwin and Milekovic studies, we believe that the go-phase neural response to grasp likely outweighed any cue-driven effects.

Comment: I am not sure of the added value of the demixing analysis in this paper, beyond the ANOVA and PCA analyses. A clearer explanation of this value would be helpful.

Response: The use of dPCA presents three distinct advantages, which we have outlined within the updated “Demixed Principal Component Analysis” section of the Methods (highlighted in yellow). We present these three points in further detail here.

First, unlike traditional PCA, dPCA is a supervised dimensionality reduction technique that takes information about task parameters (such as force and grasp) into account. While traditional PCs retain mixed selectivity to both force and grasp, dPCA is able to demix the dependencies of the population activity to these parameters. Therefore, dPCs more clearly highlight population-level neural tuning to individual task parameters and are easier to interpret than traditional PCs.

Second, since dPCA performs an ANOVA-like decomposition of the neural activity into different sources of variance, it allowed us to directly compare ANOVA-derived, single-feature tuning properties to population-level trends. We discuss this comparison within the “Single-Feature Versus Population Interactions between Force and Grasp” section of the Discussion (highlighted in blue).

Third, dPCA enabled us to quantify the degree to which forces and grasps were represented within the neural data in two ways. First, dPCA quantified the amount of neural variance that each parameter explained. Second, we used the dPCA decoding axes to quantify how well our parameters of interest could be offline-decoded from the neural activity. To the second point, dPCA allowed us to offline-decode forces and grasps while still preserving the underlying geometry of the neural data. This is in contrast to traditional offline classification techniques like linear discriminant analysis, support vector machines, and others, which prioritize parameter classification over data reconstruction. Therefore, dPCA enabled us to use a single technique to both elucidate the behavior of the neural data in low-dimensional space in an intuitive manner and quantify the degree to which this information could be utilized within an iBCI system.

Comment: I am not convinced that the results support the additive over the scalar model. It seems to me that the data do not fit either model. Is there a way to quantify the similarity to one model vs. the other? Additionally, I think that it would help to plot the pie charts (Figure 6B) without the CI fraction to compare these percentages better with those shown in Figure 5.

Response: To address your comment, we have performed an additional analysis that quantifies the degree to which our data fit the additive and scalar models, as well as a combined model that incorporates terms from both of these models, using cross-validated ordinary least squares regression. Cross-validation was performed using 100 iterations of a stratified Monte Carlo leave-group-out procedure. The results of the regression analysis, including R2 values for each model and fitted model coefficients, are presented in Figure 5D and a new supplemental Figure 5-2. Our methodology is described in more detail within a new section at the end of the Materials and Methods entitled, “Comparison of Simulated Force Encoding Models.” All associated changes to the main text and figure legends have been highlighted in yellow.

As described within the updated Results section, we found that both the additive and combined models fit the data significantly better than the scalar model (p<=0.001). Furthermore, we found that the scalar term within the combined model was often assigned a much lower weighting coefficient than the additive terms. Finally, the relatively high R2 values (∼0.7) for the additive and combined models suggest that the data do support our proposed models to a large extent - though we acknowledge that a different model than those evaluated may fit the data even better.

Regarding the pie charts in Figures 5 and 5-1, we felt that it was important to include plots with CI-related variance present in order to accurately reflect the structure of our data, as well as the degree to which forces and grasps were represented within this data. However, we also agree that versions of the pie charts that omit CI-related variance would facilitate a comparison to the additive and scalar models presented within Figure 4. Therefore, we have included both versions of these pie charts within an updated Figure 5-1.

Comment: The decoding was done on one session but the session-to-session variability in tuning fraction was very large (as evident from Figure 3). To what extent did the quality of decoding vary across sessions? Given this large variability, can we rely on these signals for long-term decoding as needed for BCI? To what extent is this variability of coding force and grasp types compared to variations in other tested parameters (such as direction of movement) obtained in other studies?

Response: To clarify, the decoding analysis was actually performed on all sessions. Session-averaged results for participant P1 are presented in Figures 6 and 7, with confidence intervals included to indicate the degree of variability in decoding quality across sessions. (Participant P2 only contributed one session, so decoding results for P2 are, by definition, session-specific.) We recognize that Figures 6 and 7 may not indicate inter-session variability as clearly or with as much nuance as much as figures containing session-specific information. Therefore, we have added a new supplemental Figure 6-3, which plots time-varying force decoding performances in participant P1 for each session individually. From this figure, decoding performance for light, medium, and hard forces is relatively consistent across multiple sessions. Similarly, time-varying grasp decoding performances in participant P1 are plotted by individual session in Figure 6-2. These decoding results support the idea that, while tuning fraction is highly variable across sessions, neural population coding as a whole remains stable. This idea is further supported by the results of the dPCA population analysis, which shows fairly consistent relationships between the amount of variance explained by force and grasp. These results show promise that force- and grasp-related signals can be utilized for long-term decoding.

Minor Comment: The Method section describing the extraction of neural features was not very clear to me. I think a better explanation of the steps used to extract these features and their similarity to more commonly used neural signals would be helpful.

Response: We have made slight alterations in word choice to improve clarity. Additionally, we have introduced brief comparisons between our extracted neural features to other common neural signals, including multiunit activity (Stark and Abeles 2007), local field potentials, and EEG signal bands.

Minor Comment: I don’t quite follow the rationale for using session 5 in P1 which contains another condition which was not tested in P2. Why not use session 6 from P1 which has a similar structure as the one used in P2? Wouldn’t this added condition interact with the results obtained for the grasp/force conditions?

Response: We featured population-level results from Session 5 within our main text for two reasons. First, we wished to present the greatest variety of data in as concise a manner as possible within the main text. Session 7 from P2 already contained the neural response to only 4 grasps, which was similar to most of the datasets collected in P1. Therefore, we chose to include Session 5 of P1 to show a greater breadth of neural responses.

Second, we wished to emphasize the ubiquitous nature of force representation across multiple grasp conditions, including one that was very different than the others (elbow extension versus power/pincer grasps). More generally, we felt that including Session 5 within the main text increased the impact and generalizability of our main findings - that force has both grasp-independent and grasp-dependent representation, that grasp-independent representation accounts for more neural variance, and that grasp is represented to a greater degree than force - precisely because these findings held even when the elbow extension condition was present.

To present a complete dataset to readers of this manuscript, we provide population-level results for the other sessions in supplemental figures, including Figures 5-1, 5-2, 6-2, 6-3, 7-1, 7-2, and 7-3. Notably Figure 5-1 has been updated to include the results of the PCA decomposition, and Figures 5-2 and 6-3 are new to this revision of the manuscript. Additionally, we present results averaged over multiple sessions in P1 within Figures 6 and 7, and we include all sessions within Figure 8. In this way, we attempted to present the entire dataset as concisely as possible, while still highlighting the influence of the elbow extension condition during Session 5.

We have included a brief rationale, highlighted in yellow, for including Session 5 within the main text at the beginning of the Results section.

Minor Comment: The CI component behaves differently in P1 vs. P2. In particular, for P2 the CI explains 80% of the variance (as opposed to 50% in P1). Is there an intuitive explanation for this difference? For example, a different location of arrays, number of task-related channels in the arrays, etc.

Response: While it is difficult to definitively state why these differences occur, multiple factors likely contributed. First, as you point out, array placements were slightly different across participants, which could have led to the sampling of different populations of neurons and thus different profiles of force, grasp, interacting, and condition-independent representations.

Additionally, as indicated in Figure 5-1, the degree to which CI (and grasp) were represented within the neural data varied widely between sessions, even in the same participant (P1). Multiple possibilities could account for this inter-session variability, including electrode micromotion, added versus dropped units detected by individual channels within the microelectrode arrays, and non-stationarities within the data. Despite this variability in CI and grasp representation, grasps were decoded with fairly consistent accuracy across sessions.

Minor Comment: In my mind, one of the most interesting results is the earlier coding of grasp type vs. force levels (Fig. 6). I was therefore a bit disappointed to learn that this was probably an outcome of the task design. Was there any attempt to reverse the arrangement of trials in blocks, where successive trials all had the same force level but a different grasp type?

Response: Unfortunately, we did not reverse the arrangement of trials, as we discovered the effects of our task design well after data collection was complete. However, it is possible (and would be consistent with other studies - Milekovic et al, 2015) that the earlier coding of grasp versus force is a true effect of the neural coding of these parameters. In Milekovic’s non-human primate study, grasp and force representation exhibited similar temporal effects as those presented in the current manuscript, even though force and grasp trials were intermixed. We hope to investigate this phenomenon further in future works.

## References

[B1] Ajiboye AB, Willett FR, Young DR, Memberg WD, Murphy BA, Miller JP, Walter BL, Sweet JA, Hoyen HA, Keith MW, Peckham PH, Simeral JD, Donoghue JP, Hochberg LR, Kirsch RF (2017) Restoration of reaching and grasping movements through brain-controlled muscle stimulation in a person with tetraplegia: a proof-of-concept demonstration. Lancet 389:1821–1830. 10.1016/S0140-6736(17)30601-3 28363483 PMC5516547

[B2] Ashe J (1997) Force and the motor cortex. Behav Brain Res 87:255–269. 10.1016/s0166-4328(97)00752-3 9331494

[B3] Benjamini Y, Hochberg Y (1995) Controlling the false discovery rate - a practical and powerful approach to multiple testing. J R Stat Soc Series B Stat Methodol 57:289–300. 10.1111/j.2517-6161.1995.tb02031.x

[B4] Bleichner MG, Jansma JM, Sellmeijer J, Raemaekers M, Ramsey NF (2014) Give me a sign: decoding complex coordinated hand movements using high-field fMRI. Brain Topogr 27:248–257. 10.1007/s10548-013-0322-x 24122368

[B5] Bleichner MG, Freudenburg ZV, Jansma JM, Aarnoutse EJ, Vansteensel MJ, Ramsey NF (2016) Give me a sign: decoding four complex hand gestures based on high-density ECoG. Brain Struct Funct 221:203–216. 10.1007/s00429-014-0902-x 25273279 PMC4720726

[B6] Boudreau MJ, Smith AM (2001) Activity in rostral motor cortex in response to predictable force-pulse perturbations in a precision grip task. J Neurophysiol 86:1079–1085. 10.1152/jn.2001.86.3.1079 11535658

[B7] Bouton CE, Shaikhouni A, Annetta NV, Bockbrader MA, Friedenberg DA, Nielson DM, Sharma G, Sederberg PB, Glenn BC, Mysiw WJ, Morgan AG, Deogaonkar M, Rezai AR (2016) Restoring cortical control of functional movement in a human with quadriplegia. Nature 533:247–250. 10.1038/nature17435 27074513

[B8] Branco MP, Freudenburg ZV, Aarnoutse EJ, Bleichner MG, Vansteensel MJ, Ramsey NF (2017) Decoding hand gestures from primary somatosensory cortex using high-density ECoG. Neuroimage 147:130–142. 10.1016/j.neuroimage.2016.12.004 27926827 PMC5322832

[B9] Branco MP, de Boer LM, Ramsey NF, Vansteensel MJ (2019) Encoding of kinetic and kinematic movement parameters in the sensorimotor cortex: a brain-computer interface perspective. Eur J Neurosci 50:2755–2772. 10.1111/ejn.14342 30633413 PMC6625947

[B10] Carmena JM, Lebedev MA, Crist RE, O'Doherty JE, Santucci DM, Dimitrov DF, Patil PG, Henriquez CS, Nicolelis MA (2003) Learning to control a brain-machine interface for reaching and grasping by primates. PLoS Biol 1:E42. 10.1371/journal.pbio.0000042 14624244 PMC261882

[B11] Carpaneto J, Umiltà MA, Fogassi L, Murata A, Gallese V, Micera S, Raos V (2011) Decoding the activity of grasping neurons recorded from the ventral premotor area F5 of the macaque monkey. Neuroscience 188:80–94. 10.1016/j.neuroscience.2011.04.062 21575688

[B12] Casadio M, Pressman A, Mussa-Ivaldi FA (2015) Learning to push and learning to move: the adaptive control of contact forces. Front Comput Neurosci 9:118. 10.3389/fncom.2015.00118 26594163 PMC4635217

[B13] Chen X, He C, Peng H (2014) Removal of muscle artifacts from single-channel EEG based on ensemble empirical mode decomposition and multiset canonical correlation analysis. J Appl Math 2014:1–10. 10.1155/2014/261347

[B14] Cheney PD, Fetz EE (1980) Functional classes of primate corticomotoneuronal cells and their relation to active force. J Neurophysiol 44:773–791. 10.1152/jn.1980.44.4.773 6253605

[B15] Chestek CA, Gilja V, Blabe CH, Foster BL, Shenoy KV, Parvizi J, Henderson JM (2013) Hand posture classification using electrocorticography signals in the gamma band over human sensorimotor brain areas. J Neural Eng 10:026002. 10.1088/1741-2560/10/2/026002 23369953 PMC3670711

[B16] Chib VS, Krutky MA, Lynch KM, Mussa-Ivaldi FA (2009) The separate neural control of hand movements and contact forces. J Neurosci 29:3939–3947. 10.1523/JNEUROSCI.5856-08.2009 19321790 PMC3462071

[B17] Christie BP, Tat DM, Irwin ZT, Gilja V, Nuyujukian P, Foster JD, Ryu SI, Shenoy KV, Thompson DE, Chestek CA (2015) Comparison of spike sorting and thresholding of voltage waveforms for intracortical brain-machine interface performance. J Neural Eng 12:016009. 10.1088/1741-2560/12/1/016009 25504690 PMC4332592

[B18] Collinger JL, Wodlinger B, Downey JE, Wang W, Tyler-Kabara EC, Weber DJ, McMorland AJ, Velliste M, Boninger ML, Schwartz AB (2013) High-performance neuroprosthetic control by an individual with tetraplegia. Lancet 381:557–564. 10.1016/S0140-6736(12)61816-9 23253623 PMC3641862

[B19] Cramer SC, Mark A, Barquist K, Nhan H, Stegbauer KC, Price R, Bell K, Odderson IR, Esselman P, Maravilla KR (2002) Motor cortex activation is preserved in patients with chronic hemiplegic stroke. Ann Neurol 52:607–616. 10.1002/ana.10351 12402258

[B20] Cramer SC, Lastra L, Lacourse MG, Cohen MJ (2005) Brain motor system function after chronic, complete spinal cord injury. Brain 128:2941–2950. 10.1093/brain/awh648 16246866

[B21] Degenhart AD, Collinger JL, Vinjamuri R, Kelly JW, Tyler-Kabara EC, Wang W (2011) Classification of hand posture from electrocorticographic signals recorded during varying force conditions. Conf Proc IEEE Eng Med Biol Soc 2011:5782–5785. 10.1109/IEMBS.2011.6091431 22255654

[B22] Downey JE, Weiss JM, Flesher SN, Thumser ZC, Marasco PD, Boninger ML, Gaunt RA, Collinger JL (2018) Implicit grasp force representation in human motor cortical recordings. Front Neurosci 12:801. 10.3389/fnins.2018.00801 30429772 PMC6220062

[B23] Ebner TJ, Hendrix CM, Pasalar S (2009) Past, present, and emerging principles in the neural encoding of movement. Adv Exp Med Biol 629:127–137. 10.1007/978-0-387-77064-2_7 19227498 PMC5070373

[B24] Ethier C, Oby ER, Bauman MJ, Miller LE (2012) Restoration of grasp following paralysis through brain-controlled stimulation of muscles. Nature 485:368–371. 10.1038/nature10987 22522928 PMC3358575

[B25] Evarts EV (1968) Relation of pyramidal tract activity to force exerted during voluntary movement. J Neurophysiol 31:14–27. 10.1152/jn.1968.31.1.14 4966614

[B26] Evarts EV (1969) Activity of pyramidal tract neurons during postural fixation. J Neurophysiol 32:375–385. 10.1152/jn.1969.32.3.375 4977837

[B27] Evarts EV, Fromm C, Kröller J, Jennings VA (1983) Motor Cortex control of finely graded forces. J Neurophysiol 49:1199–1215. 10.1152/jn.1983.49.5.1199 6864246

[B28] Fetz EE, Cheney PD (1980) Postspike facilitation of forelimb muscle activity by primate corticomotoneuronal cells. J Neurophysiol 44:751–772. 10.1152/jn.1980.44.4.751 6253604

[B29] Filimon F, Nelson JD, Hagler DJ, Sereno MI (2007) Human cortical representations for reaching: mirror neurons for execution, observation, and imagery. Neuroimage 37:1315–1328. 10.1016/j.neuroimage.2007.06.008 17689268 PMC2045689

[B30] Flesher SN, Collinger JL, Foldes ST, Weiss JM, Downey JE, Tyler-Kabara EC, Bensmaia SJ, Schwartz AB, Boninger ML, Gaunt RA (2016) Intracortical microstimulation of human somatosensory cortex. Sci Transl Med 8:361ra141. 10.1126/scitranslmed.aaf8083 27738096

[B31] Flint RD, Ethier C, Oby ER, Miller LE, Slutzky MW (2012) Local field potentials allow accurate decoding of muscle activity. J Neurophysiol 108:18–24. 10.1152/jn.00832.2011 22496527 PMC3434606

[B32] Flint RD, Wang PT, Wright ZA, King CE, Krucoff MO, Schuele SU, Rosenow JM, Hsu FP, Liu CY, Lin JJ, Sazgar M, Millett DE, Shaw SJ, Nenadic Z, Do AH, Slutzky MW (2014) Extracting kinetic information from human motor cortical signals. Neuroimage 101:695–703. 10.1016/j.neuroimage.2014.07.049 25094020 PMC12967291

[B33] Flint RD, Rosenow JM, Tate MC, Slutzky MW (2017) Continuous decoding of human grasp kinematics using epidural and subdural signals. J Neural Eng 14:016005. 10.1088/1741-2560/14/1/016005 27900947 PMC5528155

[B34] Georgopoulos AP, Kalaska JF, Caminiti R, Massey JT (1982) On the relations between the direction of two-dimensional arm movements and cell discharge in primate motor cortex. J Neurosci 2:1527–1537. 7143039 10.1523/JNEUROSCI.02-11-01527.1982 PMC6564361

[B35] Georgopoulos AP, Kalaska JF, Caminiti R, Massey JT (1983) Interruption of motor cortical discharge subserving aimed arm movements. Exp Brain Res 49:327–340. 10.1007/BF00238775 6641831

[B36] Georgopoulos AP, Schwartz AB, Kettner RE (1986) Neuronal population coding of movement direction. Science 233:1416–1419. 10.1126/science.3749885 3749885

[B37] Georgopoulos AP, Ashe J, Smyrnis N, Taira M (1992) The motor cortex and the coding of force. Science 256:1692–1695. 10.1126/science.256.5064.1692 1609282

[B38] Hao Y, Zhang Q, Controzzi M, Cipriani C, Li Y, Li J, Zhang S, Wang Y, Chen W, Chiara Carrozza M, Zheng X (2014) Distinct neural patterns enable grasp types decoding in monkey dorsal premotor cortex. J Neural Eng 11:066011. 10.1088/1741-2560/11/6/066011 25380169

[B39] Hendrix CM, Mason CR, Ebner TJ (2009) Signaling of grasp dimension and grasp force in dorsal premotor cortex and primary motor cortex neurons during reach to grasp in the monkey. J Neurophysiol 102:132–145. 10.1152/jn.00016.2009 19403752 PMC2712255

[B40] Hepp-Reymond M, Kirkpatrick-Tanner M, Gabernet L, Qi HX, Weber B (1999) Context-dependent force coding in motor and premotor cortical areas. Exp Brain Res 128:123–133. 10.1007/s002210050827 10473750

[B41] Hermes D, Vansteensel MJ, Albers AM, Bleichner MG, Benedictus MR, Mendez Orellana C, Aarnoutse EJ, Ramsey NF (2011) Functional MRI-based identification of brain areas involved in motor imagery for implantable brain-computer interfaces. J Neural Eng 8:025007. 10.1088/1741-2560/8/2/025007 21436535

[B42] Hochberg LR, Serruya MD, Friehs GM, Mukand JA, Saleh M, Caplan AH, Branner A, Chen D, Penn RD, Donoghue JP (2006) Neuronal ensemble control of prosthetic devices by a human with tetraplegia. Nature 442:164–171. 10.1038/nature04970 16838014

[B43] Hochberg LR, Bacher D, Jarosiewicz B, Masse NY, Simeral JD, Vogel J, Haddadin S, Liu J, Cash SS, van der Smagt P, Donoghue JP (2012) Reach and grasp by people with tetraplegia using a neurally controlled robotic arm. Nature 485:372–375. 10.1038/nature11076 22596161 PMC3640850

[B44] Humphrey DR (1970) A chronically implantable multiple micro-electrode system with independent control of electrode positions. Electroencephalogr Clin Neurophysiol 29:616–620. 10.1016/0013-4694(70)90105-7 4098585

[B45] Intveld RW, Dann B, Michaels JA, Scherberger H (2018) Neural coding of intended and executed grasp force in macaque areas AIP, F5, and M1. Sci Rep 8:17985. 10.1038/s41598-018-35488-z 30573765 PMC6301980

[B46] Juric D (2020) MultiClass LDA. Matlab Central File Exchange.

[B47] Kakei S, Hoffman DS, Strick PL (1999) Muscle and movement representations in the primary motor cortex. Science 285:2136–2139. 10.1126/science.285.5436.2136 10497133

[B48] Kalaska JF (2009) From intention to action: motor cortex and the control of reaching movements. Adv Exp Med Biol 629:139–178. 10.1007/978-0-387-77064-2_8 19227499

[B49] Kalaska JF, Hyde ML (1985) Area 4 and area 5: differences between the load direction-dependent discharge variability of cells during active postural fixation. Exp Brain Res 59:197–202. 10.1007/BF00237679 3926528

[B50] Kalaska JF, Cohen DA, Hyde ML, Prud'homme M (1989) A comparison of movement direction-related versus load direction-related activity in primate motor cortex, using a two-dimensional reaching task. J Neurosci 9:2080–2102. 10.1523/JNEUROSCI.09-06-02080.1989 2723767 PMC6569743

[B51] Kim SP, Simeral JD, Hochberg LR, Donoghue JP, Black MJ (2008) Neural control of computer cursor velocity by decoding motor cortical spiking activity in humans with tetraplegia. J Neural Eng 5:455–476. 10.1088/1741-2560/5/4/010 19015583 PMC2911243

[B52] Kim SP, Simeral JD, Hochberg LR, Donoghue JP, Friehs GM, Black MJ (2011) Point-and-click cursor control with an intracortical neural interface system by humans with tetraplegia. IEEE Trans Neural Syst Rehabil Eng 19:193–203. 10.1109/TNSRE.2011.2107750 21278024 PMC3294291

[B53] Klaes C, Kellis S, Aflalo T, Lee B, Pejsa K, Shanfield K, Hayes-Jackson S, Aisen M, Heck C, Liu C, Andersen RA (2015) Hand Shape Representations in the Human Posterior Parietal Cortex. J Neurosci 35:15466–15476. 10.1523/JNEUROSCI.2747-15.2015 26586832 PMC4649012

[B54] Kobak D, Brendel W, Constantinidis C, Feierstein CE, Kepecs A, Mainen ZF, Qi XL, Romo R, Uchida N, Machens CK (2016) Demixed principal component analysis of neural population data. Elife 5:e10989. 10.7554/eLife.10989 27067378 PMC4887222

[B55] Kübler A, Nijboer F, Mellinger J, Vaughan TM, Pawelzik H, Schalk G, McFarland DJ, Birbaumer N, Wolpaw JR (2005) Patients with ALS can use sensorimotor rhythms to operate a brain-computer interface. Neurology 64:1775–1777. 10.1212/01.WNL.0000158616.43002.6D 15911809

[B56] Leo A, Handjaras G, Bianchi M, Marino H, Gabiccini M, Guidi A, Scilingo EP, Pietrini P, Bicchi A, Santello M, Ricciardi E (2016) A synergy-based hand control is encoded in human motor cortical areas. Elife 5:e13420. 10.7554/eLife.13420 26880543 PMC4786436

[B57] Leuthardt EC, Schalk G, Wolpaw JR, Ojemann JG, Moran DW (2004) A brain-computer interface using electrocorticographic signals in humans. J Neural Eng 1:63–71. 10.1088/1741-2560/1/2/001 15876624

[B58] Mason CR, Theverapperuma LS, Hendrix CM, Ebner TJ (2004) Monkey hand postural synergies during reach-to-grasp in the absence of vision of the hand and object. J Neurophysiol 91:2826–2837. 10.1152/jn.00653.2003 14762155

[B59] Mason CR, Hendrix CM, Ebner TJ (2006) Purkinje cells signal hand shape and grasp force during reach-to-grasp in the monkey. J Neurophysiol 95:144–158. 10.1152/jn.00492.2005 16162833

[B60] Milekovic T, Truccolo W, Grün S, Riehle A, Brochier T (2015) Local field potentials in primate motor cortex encode grasp kinetic parameters. Neuroimage 114:338–355. 10.1016/j.neuroimage.2015.04.008 25869861 PMC4562281

[B61] Mizuguchi N, Nakamura M, Kanosue K (2017) Task-dependent engagements of the primary visual cortex during kinesthetic and visual motor imagery. Neurosci Lett 636:108–112. 10.1016/j.neulet.2016.10.064 27826015

[B62] Moritz CT, Perlmutter SI, Fetz EE (2008) Direct control of paralysed muscles by cortical neurons. Nature 456:639–642. 10.1038/nature07418 18923392 PMC3159518

[B63] Morrow MM, Miller LE (2003) Prediction of muscle activity by populations of sequentially recorded primary motor cortex neurons. J Neurophysiol 89:2279–2288. 10.1152/jn.00632.2002 12612022 PMC2586069

[B64] Murphy BA, Miller JP, Gunalan K, Ajiboye AB (2016) Contributions of Subsurface Cortical Modulations to Discrimination of Executed and Imagined Grasp Forces through Stereoelectroencephalography. PLoS One 11:e0150359. 10.1371/journal.pone.0150359 26963246 PMC4786254

[B65] Nuyujukian P, Albites Sanabria J, Saab J, Pandarinath C, Jarosiewicz B, Blabe CH, Franco B, Mernoff ST, Eskandar EN, Simeral JD, Hochberg LR, Shenoy KV, Henderson JM (2018) Cortical control of a tablet computer by people with paralysis. PLoS One 13:e0204566. 10.1371/journal.pone.0204566 30462658 PMC6248919

[B66] Oby ER, Ethier C, Bauman MJ, Perreault EJ, Ko JH, Miller LE (2010) Prediction of muscle activity from cortical signals to restore hand grasp in subjects with spinal cord injury. In: Statistical signal processing for neuroscience and neurotechnology, pp 369–406. San Diego: Elsevier Inc.

[B67] Paek AY, Gailey A, Parikh P, Santello M, Contreras-Vidal J (2015) Predicting hand forces from scalp electroencephalography during isometric force production and object grasping. Conf Proc IEEE Eng Med Biol Soc 2015:7570–7573. 10.1109/EMBC.2015.7320144 26738044

[B68] Picard N, Smith AM (1992) Primary motor cortical responses to perturbations of prehension in the monkey. J Neurophysiol 68:1882–1894. 10.1152/jn.1992.68.5.1882 1479451

[B69] Pistohl T, Schulze-Bonhage A, Aertsen A, Mehring C, Ball T (2012) Decoding natural grasp types from human ECoG. Neuroimage 59:248–260. 10.1016/j.neuroimage.2011.06.084 21763434

[B70] Pohlmeyer EA, Solla SA, Perreault EJ, Miller LE (2007) Prediction of upper limb muscle activity from motor cortical discharge during reaching. J Neural Eng 4:369–379. 10.1088/1741-2560/4/4/003 18057504 PMC2586074

[B71] Pohlmeyer EA, Oby ER, Perreault EJ, Solla SA, Kilgore KL, Kirsch RF, Miller LE (2009) Toward the restoration of hand use to a paralyzed monkey: brain-controlled functional electrical stimulation of forearm muscles. PLoS One 4:e5924. 10.1371/journal.pone.0005924 19526055 PMC2691481

[B72] Rastogi A, Vargas-Irwin CE, Willett FR, Abreu J, Crowder DC, Murphy BA, Memberg WD, Miller JP, Sweet JA, Walter BL, Cash SS, Rezaii PG, Franco B, Saab J, Stavisky SD, Shenoy KV, Henderson JM, Hochberg LR, Kirsch RF, Ajiboye AB (2020) Neural representation of observed, imagined, and attempted grasping force in motor cortex of individuals with chronic tetraplegia. Sci Rep 10:1429. 10.1038/s41598-020-58097-1 31996696 PMC6989675

[B73] Rathelot JA, Strick PL (2009) Subdivisions of primary motor cortex based on cortico-motoneuronal cells. Proc Natl Acad Sci USA 106:918–923. 10.1073/pnas.0808362106 19139417 PMC2621250

[B74] Sburlea AI, Müller-Putz GR (2018) Exploring representations of human grasping in neural, muscle and kinematic signals. Sci Rep 8:16669. 10.1038/s41598-018-35018-x 30420724 PMC6232146

[B75] Schaffelhofer S, Agudelo-Toro A, Scherberger H (2015) Decoding a wide range of hand configurations from macaque motor, premotor, and parietal cortices. J Neurosci 35:1068–1081. 10.1523/JNEUROSCI.3594-14.2015 25609623 PMC6605542

[B76] Schalk G, Miller KJ, Anderson NR, Wilson JA, Smyth MD, Ojemann JG, Moran DW, Wolpaw JR, Leuthardt EC (2008) Two-dimensional movement control using electrocorticographic signals in humans. J Neural Eng 5:75–84. 10.1088/1741-2560/5/1/008 18310813 PMC2744037

[B77] Schiefer MA, Graczyk EL, Sidik SM, Tan DW, Tyler DJ (2018) Artificial tactile and proprioceptive feedback improves performance and confidence on object identification tasks. PLoS One 13:e0207659. 10.1371/journal.pone.0207659 30517154 PMC6281191

[B78] Schwarz A, Ofner P, Pereira J, Sburlea AI, Müller-Putz GR (2018) Decoding natural reach-and-grasp actions from human EEG. J Neural Eng 15:e016005. 10.1088/1741-2552/aa8911 28853420

[B79] Sergio LE, Kalaska JF (1998) Changes in the temporal pattern of primary motor cortex activity in a directional isometric force versus limb movement task. J Neurophysiol 80:1577–1583. 10.1152/jn.1998.80.3.1577 9744964

[B80] Sergio LE, Kalaska JF (2003) Systematic changes in motor cortex cell activity with arm posture during directional isometric force generation. J Neurophysiol 89:212–228. 10.1152/jn.00016.2002 12522173

[B81] Sergio LE, Hamel-Pâquet C, Kalaska JF (2005) Motor cortex neural correlates of output kinematics and kinetics during isometric-force and arm-reaching tasks. J Neurophysiol 94:2353–2378. 10.1152/jn.00989.2004 15888522

[B82] Simeral JD, Kim SP, Black MJ, Donoghue JP, Hochberg LR (2011) Neural control of cursor trajectory and click by a human with tetraplegia 1000 days after implant of an intracortical microelectrode array. J Neural Eng 8:e025027. 10.1088/1741-2560/8/2/025027 21436513 PMC3715131

[B83] Smith AM, Hepp-Reymond MC, Wyss UR (1975) Relation of activity in precentral cortical neurons to force and rate of force change during isometric contractions of finger muscles. Exp Brain Res 23:315–332. 10.1007/BF00239743 810360

[B84] Solstrand Dahlberg L, Becerra L, Borsook D, Linnman C (2018) Brain changes after spinal cord injury, a quantitative meta-analysis and review. Neurosci Biobehav Rev 90:272–293. 10.1016/j.neubiorev.2018.04.018 29702136

[B85] Stark E, Abeles M (2007) Predicting movement from multiunit activity. J Neurosci 27:8387–8394. 10.1523/JNEUROSCI.1321-07.2007 17670985 PMC6673077

[B86] Stark E, Asher I, Abeles M (2007) Encoding of reach and grasp by single neurons in premotor cortex is independent of recording site. J Neurophysiol 97:3351–3364. 10.1152/jn.01328.2006 17360824

[B87] Stevens JA (2005) Interference effects demonstrate distinct roles for visual and motor imagery during the mental representation of human action. Cognition 95:329–350. 10.1016/j.cognition.2004.02.008 15788162

[B88] Tabot GA, Kim SS, Winberry JE, Bensmaia SJ (2015) Restoring tactile and proprioceptive sensation through a brain interface. Neurobiol Dis 83:191–198. 10.1016/j.nbd.2014.08.029 25201560 PMC4362964

[B89] Taira M, Boline J, Smyrnis N, Georgopoulos AP, Ashe J (1996) On the relations between single cell activity in the motor cortex and the direction and magnitude of three-dimensional static isometric force. Exp Brain Res 109:367–376. 10.1007/BF00229620 8817266

[B90] Tan DW, Schiefer MA, Keith MW, Anderson JR, Tyler J, Tyler DJ (2014) A neural interface provides long-term stable natural touch perception. Sci Transl Med 6:257ra138. 10.1126/scitranslmed.3008669 25298320 PMC5517305

[B91] Thach WT (1978) Correlation of neural discharge with pattern and force of muscular activity, joint position, and direction of intended next movement in motor cortex and cerebellum. J Neurophysiol 41:654–676. 10.1152/jn.1978.41.3.654 96223

[B92] Townsend BR, Subasi E, Scherberger H (2011) Grasp movement decoding from premotor and parietal cortex. J Neurosci 31:14386–14398. 10.1523/JNEUROSCI.2451-11.2011 21976524 PMC6623645

[B93] Trautmann EM, Stavisky SD, Lahiri S, Ames KC, Kaufman MT, O'Shea DJ, Vyas S, Sun X, Ryu SI, Ganguli S, Shenoy KV (2019) Accurate estimation of neural population dynamics without spike sorting. Neuron 103:292–308.e4. 10.1016/j.neuron.2019.05.003 31171448 PMC7002296

[B94] Vargas-Irwin CE, Shakhnarovich G, Yadollahpour P, Mislow JM, Black MJ, Donoghue JP (2010) Decoding complete reach and grasp actions from local primary motor cortex populations. J Neurosci 30:9659–9669. 10.1523/JNEUROSCI.5443-09.2010 20660249 PMC2921895

[B95] Vargas-Irwin CE, Feldman JM, King B, Simeral JD, Sorice BL, Oakley EM, Cash SS, Eskandar EN, Friehs GM, Hochberg LR, Donoghue JP (2018) Watch, imagine, attempt: motor cortex single-unit activity reveals context-dependent movement encoding in humans with tetraplegia. Front Hum Neurosci 12:450. 10.3389/fnhum.2018.00450 30524258 PMC6262367

[B96] Wannier TM, Maier MA, Hepp-Reymond MC (1991) Contrasting properties of monkey somatosensory and motor cortex neurons activated during the control of force in precision grip. J Neurophysiol 65:572–589. 10.1152/jn.1991.65.3.572 2051196

[B97] Westling G, Johansson RS (1984) Factors influencing the force control during precision grip. Exp Brain Res 53:277–284. 10.1007/BF00238156 6705863

[B98] Wilcox RR (2017) Introdction to robust estimation and hypothesis testing, Ed 3. Cambridge: Academic Press.

[B99] Willett FR, Deo DR, Avansino DT, Rezaii P, Hochberg LR, Henderson JM, Shenoy KV (2020) Hand knob area of premotor cortex represents the whole body in a compositional way. Cell 181:396–409.e26. 10.1016/j.cell.2020.02.043 32220308 PMC7166199

[B100] Wodlinger B, Downey JE, Tyler-Kabara EC, Schwartz AB, Boninger ML, Collinger JL (2015) Ten-dimensional anthropomorphic arm control in a human brain-machine interface: difficulties, solutions, and limitations. J Neural Eng 12:016011. 10.1088/1741-2560/12/1/016011 25514320

[B101] Wolpaw JR, Birbaumer N, McFarland DJ, Pfurtscheller G, Vaughan TM (2002) Brain-computer interfaces for communication and control. Clin Neurophysiol 113:767–791. 10.1016/s1388-2457(02)00057-3 12048038

[B102] Yousry TA, Schmid UD, Alkadhi H, Schmidt D, Peraud A, Buettner A, Winkler P (1997) Localization of the motor hand area to a knob on the precentral gyrus. A new landmark. Brain 120:141–157. 10.1093/brain/120.1.141 9055804

