# Supplementary figures and images for: The Neural Representation of Force across Grasp Types in Motor Cortex of Humans with Tetraplegia
Source: eNeuro. 2021 Feb 23;8(1):ENEURO.0231-20.2020. doi: 10.1523/ENEURO.0231-20.2020 (PMC7920535; doi:10.1523/ENEURO.0231-20.2020)

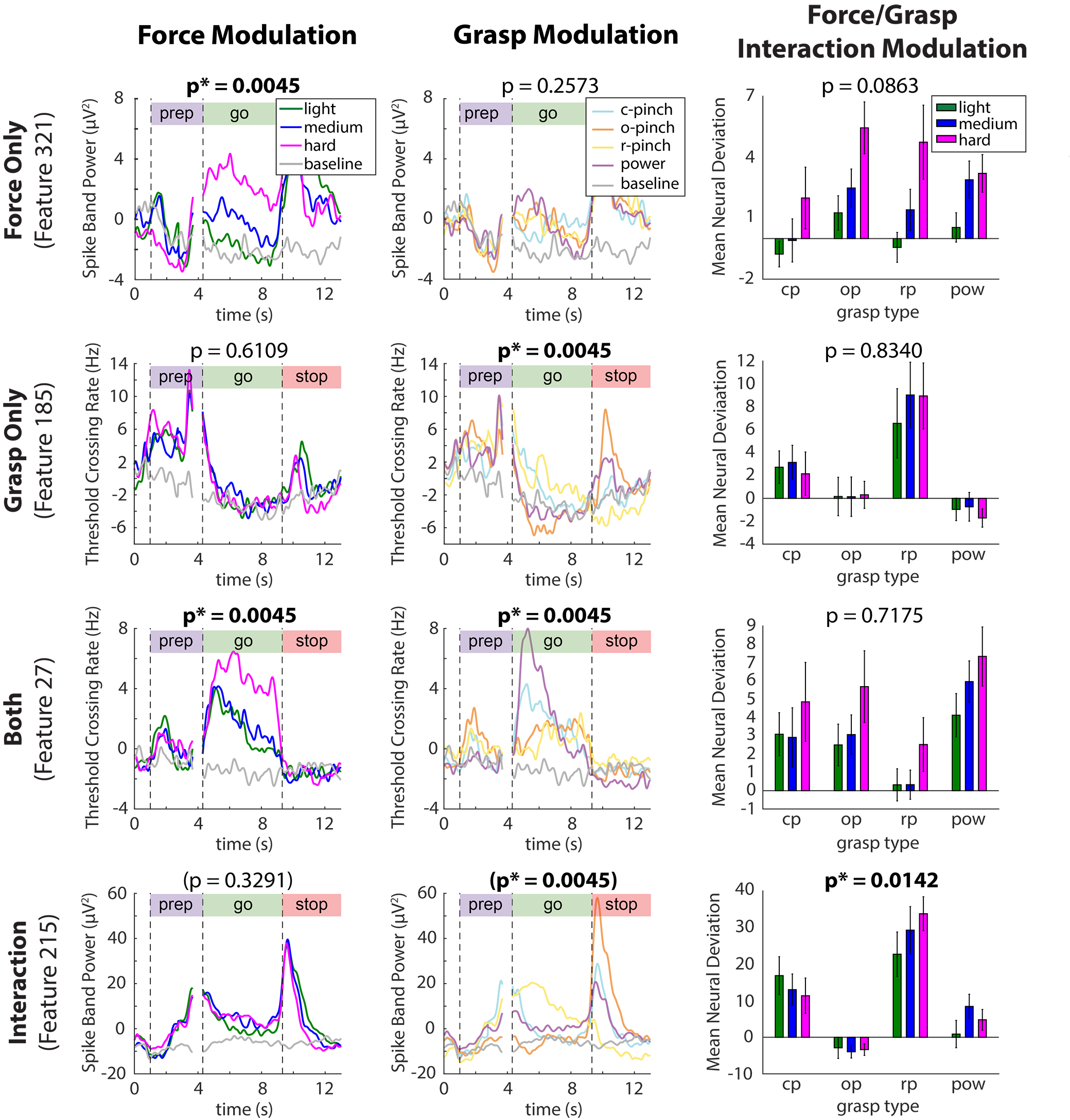

Supplement: Extended Data Figure 2-1 — Exemplary TC and SBP features tuned to task parameters of interest in participant T5, presented as in Figure 2. Note the presence of sharp activity peaks during the prep and stop phases of the trial, which were due to the presence of visual cues (Rastogi et al, 2020). Download Figure 2-1, TIF file. [file enu-eN-NWR-0231-20-s02.tif]

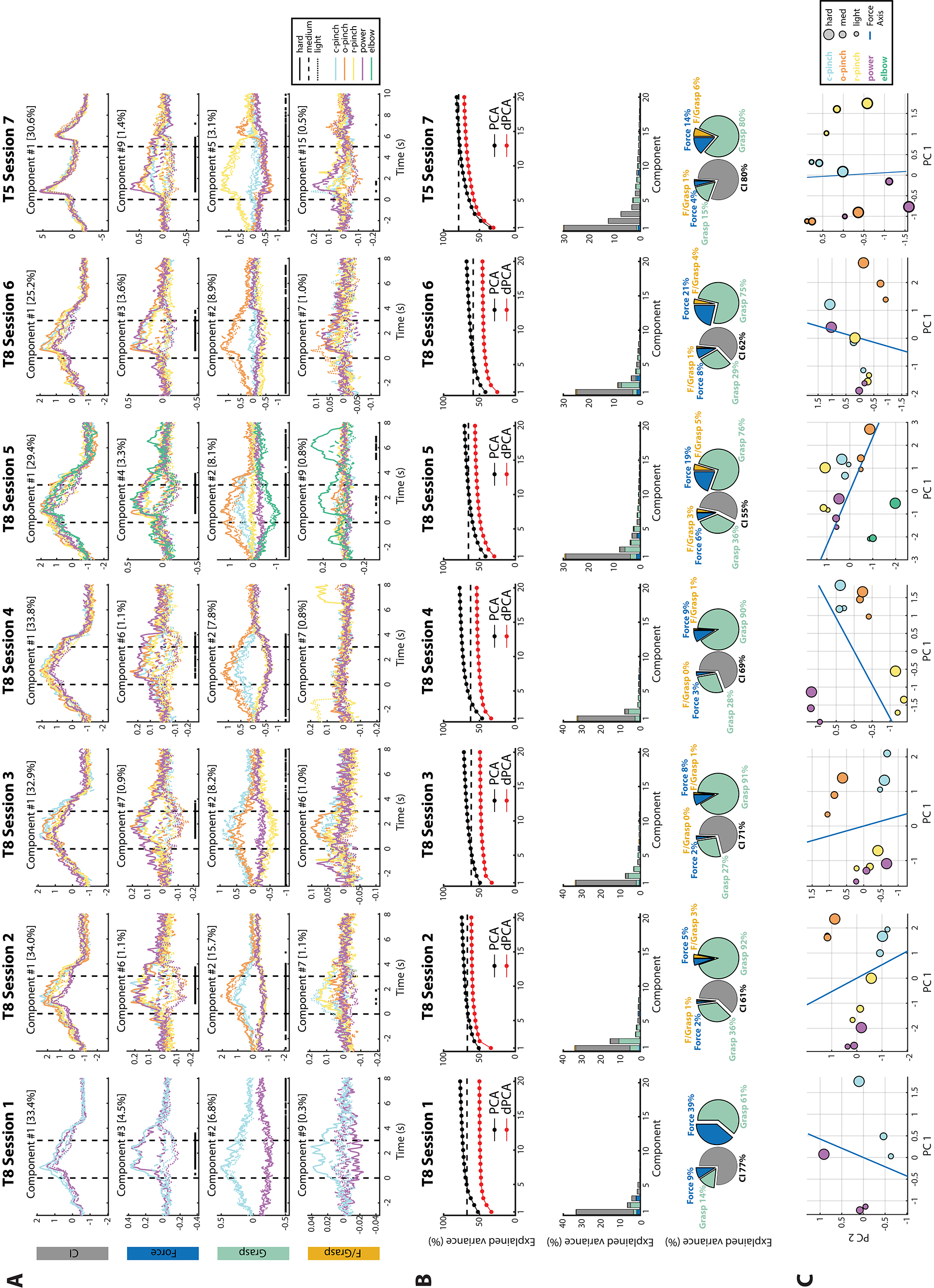

Supplement: Extended Data Figure 5-1 — Neural population-level activity patterns for all sessions, presented as in Figure 5A–C. A, dPCs isolated from all individual sessions of neural data. B, Summary of variances accounted for by the top 20 dPCs from each exemplary session. Pie charts indicate the percentage of total signal variance accounted for by each marginalization. Total signal variance was computed with (left) and without (right) the condition-independent portion of the signal, as a basis of comparison to Figure 4 of the main text. C, Go-phase activity within two-dimensional PCA space. This figure shows dPCs, variances, and PCA plots for all recorded sessions. Corresponding encoding model performances for all recorded sessions appear in Extended Data Figure 5-2. Download Figure 5-1, TIF file. [file enu-eN-NWR-0231-20-s03.tif]

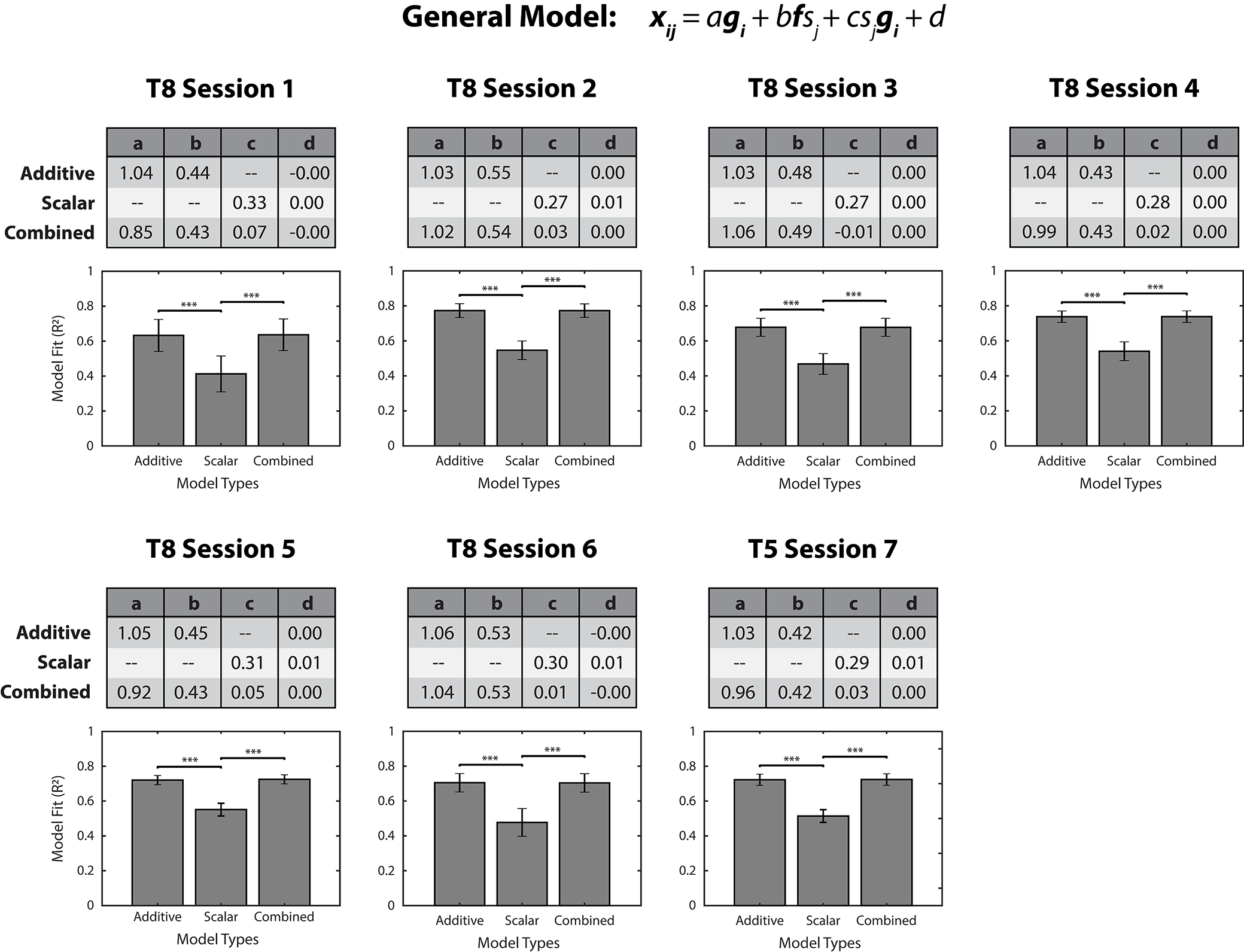

Supplement: Extended Data Figure 5-2 — Encoding model performances, presented as in Figure 5D. Download Figure 5-2, TIF file. [file enu-eN-NWR-0231-20-s04.tif]

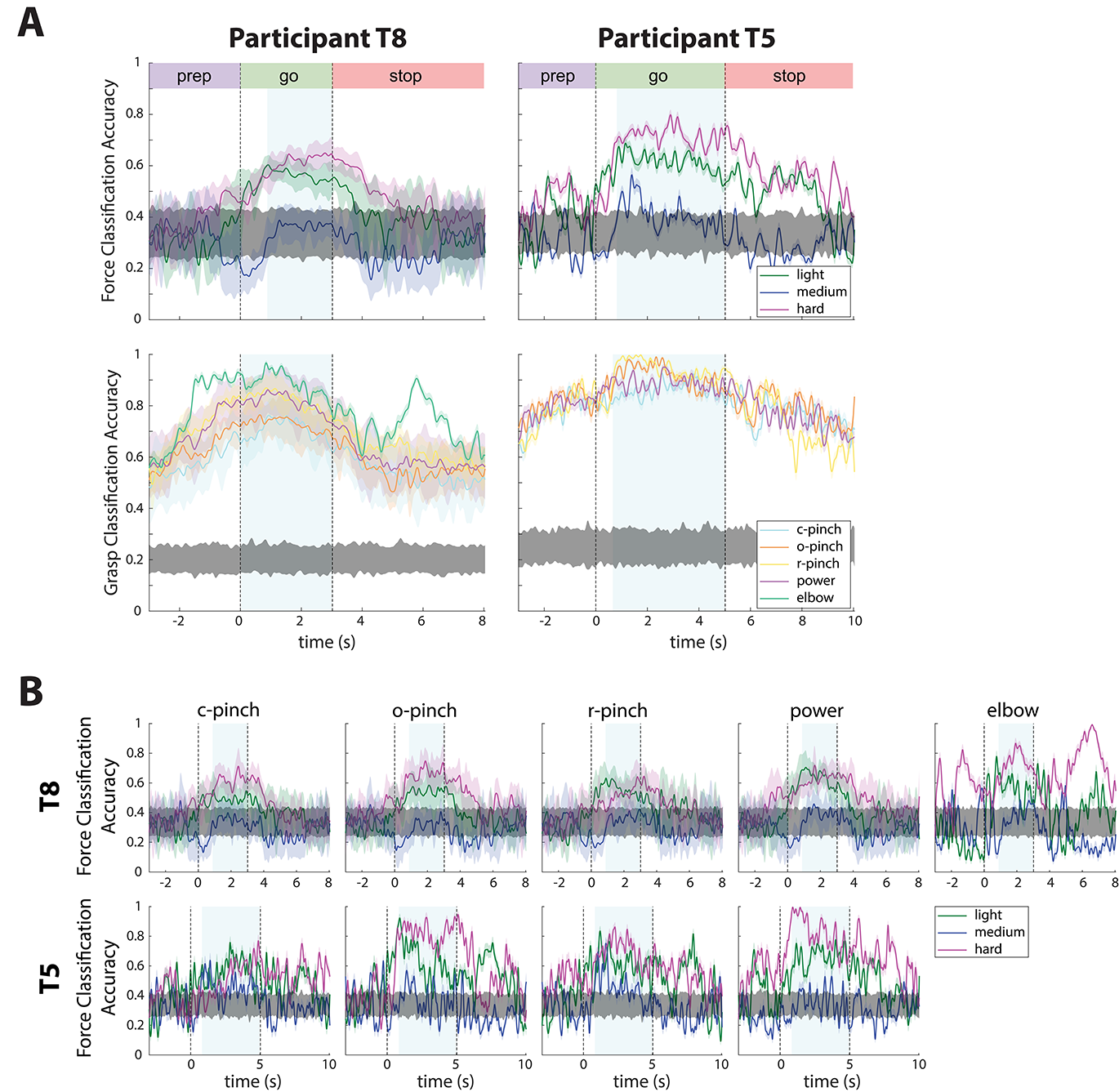

Supplement: Extended Data Figure 6-1 — Time-dependent classification accuracies for individual force levels and grasp types. A, Time-dependent classification accuracies for force (row 1) and grasp (row 2), separated by force class and grasp class, respectively. Data traces were smoothed with a 100-ms boxcar filter to aid in in visualization. Shaded areas surrounding each data trace indicate the SD across 240 session-runs during most trials in participant T8, 40 session-runs during elbow extension trials in participant T8, and 40-session runs in participant T5. Gray shaded regions indicate the upper and lower bounds of chance performance over S × 100 shuffles of trial data, where S is the number of sessions per participant. Blue shaded regions indicate the time points used to compute go-phase confusion matrices. B, Time-dependent force classification accuracies during individual grasps in participants T8 (row 1) and T5 (row 2). Blue shaded regions indicate the time points used to compute go-phase confusion matrices. Decoding performances were averaged over S × 40 session runs, where S is the number of sessions per participant. Download Figure 6-1, TIF file. [file enu-eN-NWR-0231-20-s05.tif]

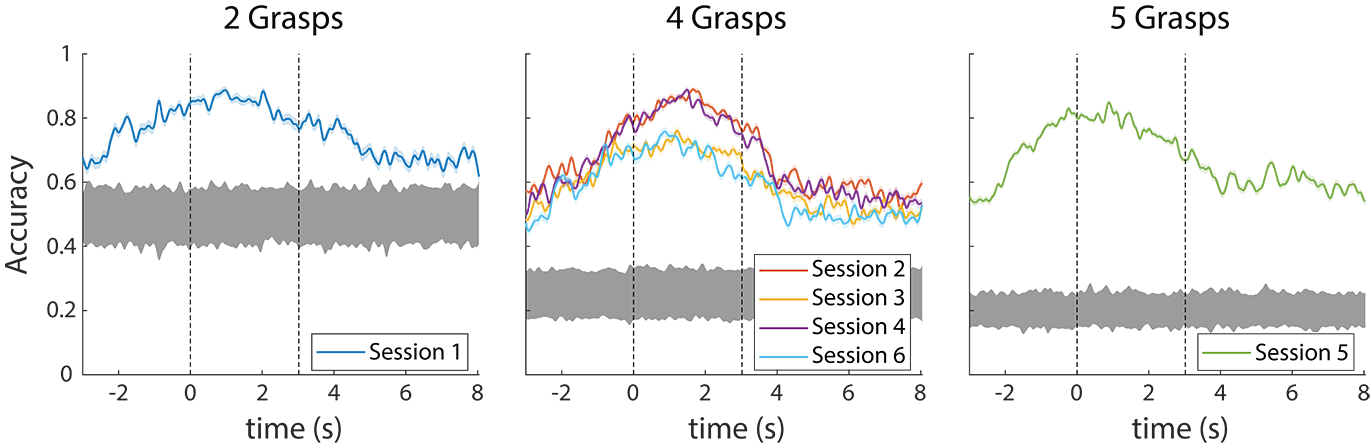

Supplement: Extended Data Figure 6-2 — Time-dependent grasp classification accuracies by number of grasps attempted per session in participant T8. Data traces were smoothed with a 100-ms boxcar filter to aid in in visualization. Shaded areas surrounding each data trace indicate the SD across 40 runs during each session in participant T8. Gray shaded regions indicate the upper and lower bounds of chance performance over 100 shuffles of trial data per session. Intended grasp is classified above chance performance at all trial time points, regardless of the number of grasps to be decoded. Download Figure 6-2, TIF file. [file enu-eN-NWR-0231-20-s06.tif]

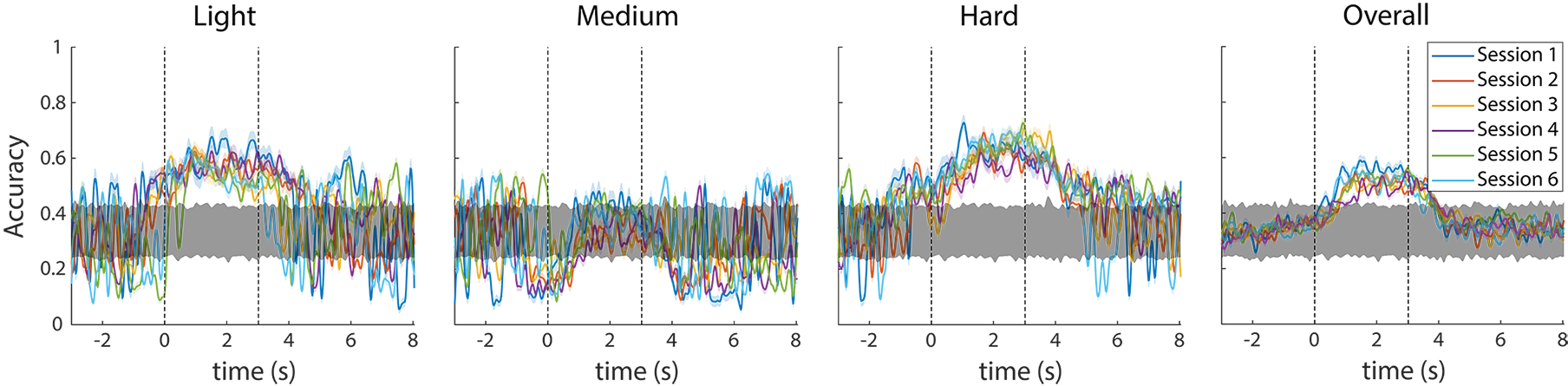

Supplement: Extended Data Figure 6-3 — Time-dependent force classification accuracies by force level, per session, in participant T8. Data traces were smoothed with a 100-ms boxcar filter to aid in in visualization. Shaded areas surrounding each data trace indicate the SD across 40 runs during each session in participant T8. Gray shaded regions indicate the upper and lower bounds of chance performance over 100 shuffles of trial data per session. Intended grasp is classified above chance performance at all trial time points, regardless of the number of grasps to be decoded. Download Figure 6-3, TIF file. [file enu-eN-NWR-0231-20-s07.tif]

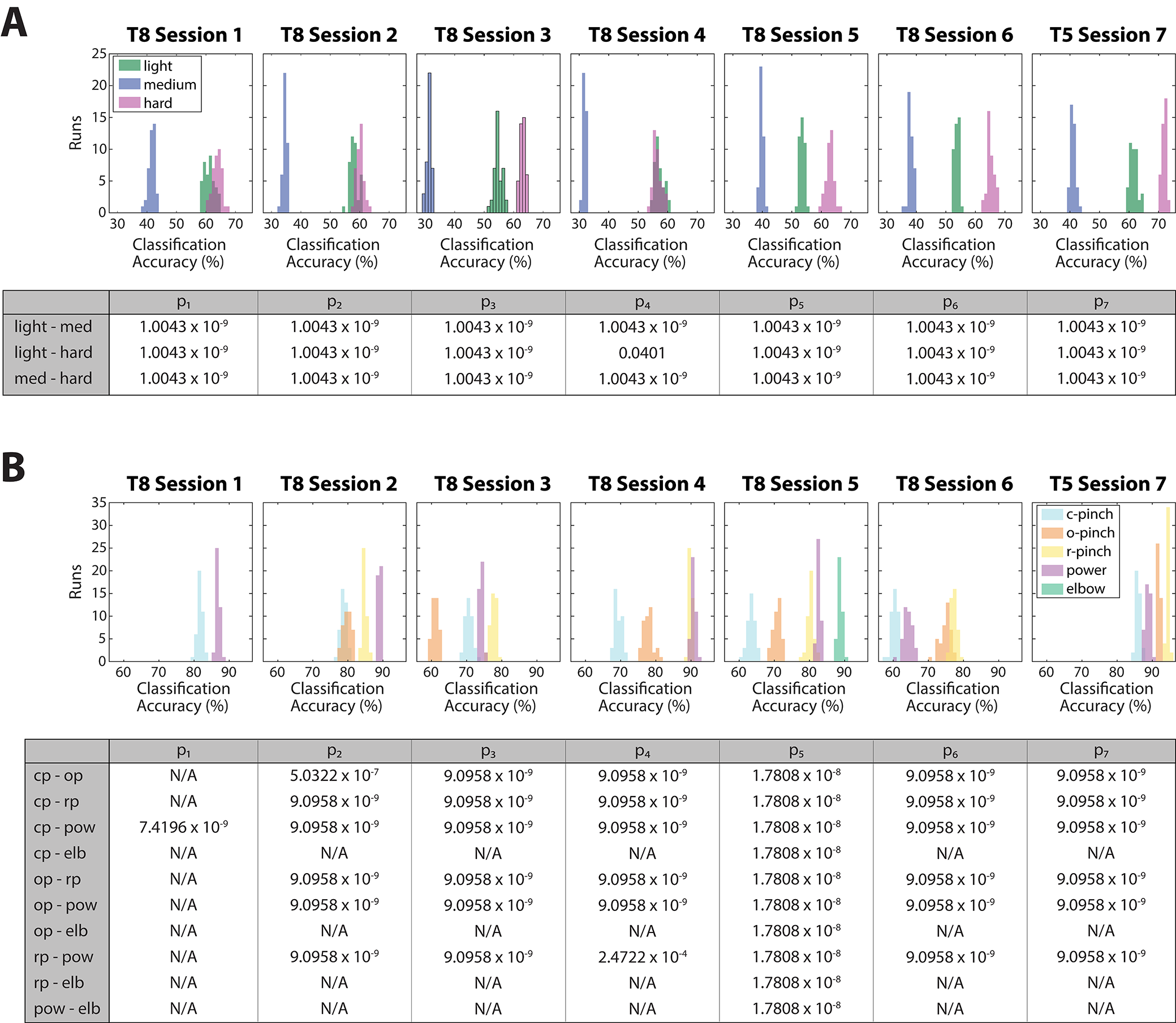

Supplement: Extended Data Figure 7-1 — Statistics for go-phase force and grasp classifications accuracies. A, Force classification accuracy histograms (row 1) and corrected p values (row 2). Hard and light forces are classified significantly more accurately than medium forces across all sessions (p < 0.05). B, Grasp classification accuracy histograms (row 1) and corrected p values (row 2). Decoding performance differed significantly between grasps across all sessions. Download Figure 7-1, TIF file. [file enu-eN-NWR-0231-20-s08.tif]

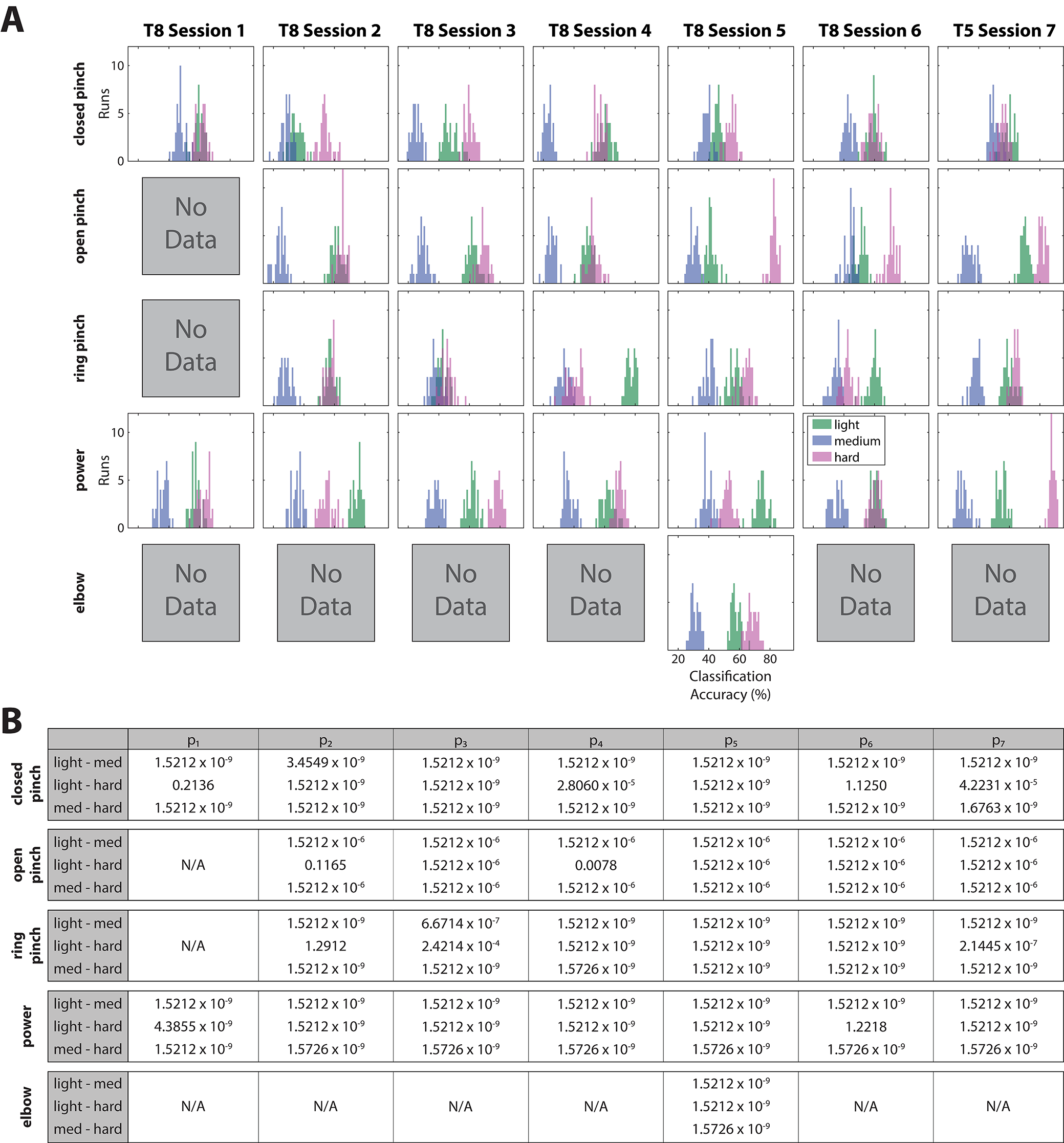

Supplement: Extended Data Figure 7-2 — Statistics for go-phase force classification accuracies within individual grasp types. A one-way ANOVA was implemented on force classification accuracies achieved during different grasp types. A, Force classification accuracy histograms. B, p values between force pairs, corrected for multiple comparisons across grasps and sessions using the Benjamini–Hochberg procedure. Within each grasp, hard and light forces were classified more accurately than medium forces across all sessions (p < 0.05). Download Figure 7-2, TIF file. [file enu-eN-NWR-0231-20-s09.tif]

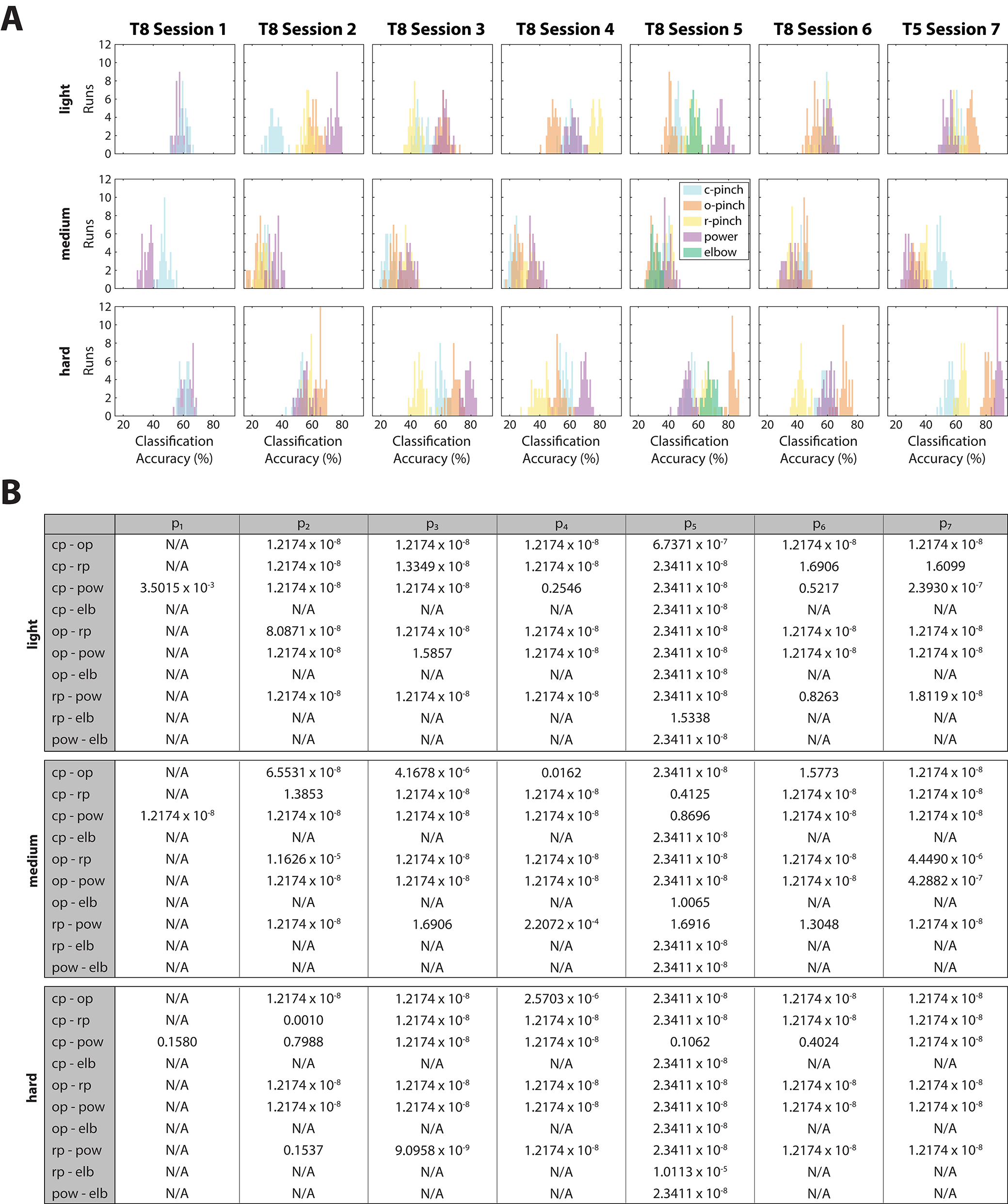

Supplement: Extended Data Figure 7-3 — Statistics for go-phase force classification accuracies within individual force levels. A one-way ANOVA was implemented on the force classification accuracies achieved during different grasp types. A, Force classification accuracy histograms, color-coded by the grasp type used to produce each force level. B, p values between pairs of grasps used to produce each individual force level, corrected for multiple comparisons across forces and sessions using the Benjamini–Hochberg procedure. The decoding performance for each discrete force level was significantly different across grasps (p < 0.05), indicating that grasp type affected force decoding performance. Download Figure 7-3, TIF file. [file enu-eN-NWR-0231-20-s10.tif]
